# Supplementary figures and images for: Peroxisome-derived ether lipids regulate lysosomal exocytosis
Source: EMBO J. 2026 May 2;45(11):3699–730. doi: 10.1038/s44318-026-00791-3 (PMC13226661; doi:10.1038/s44318-026-00791-3)

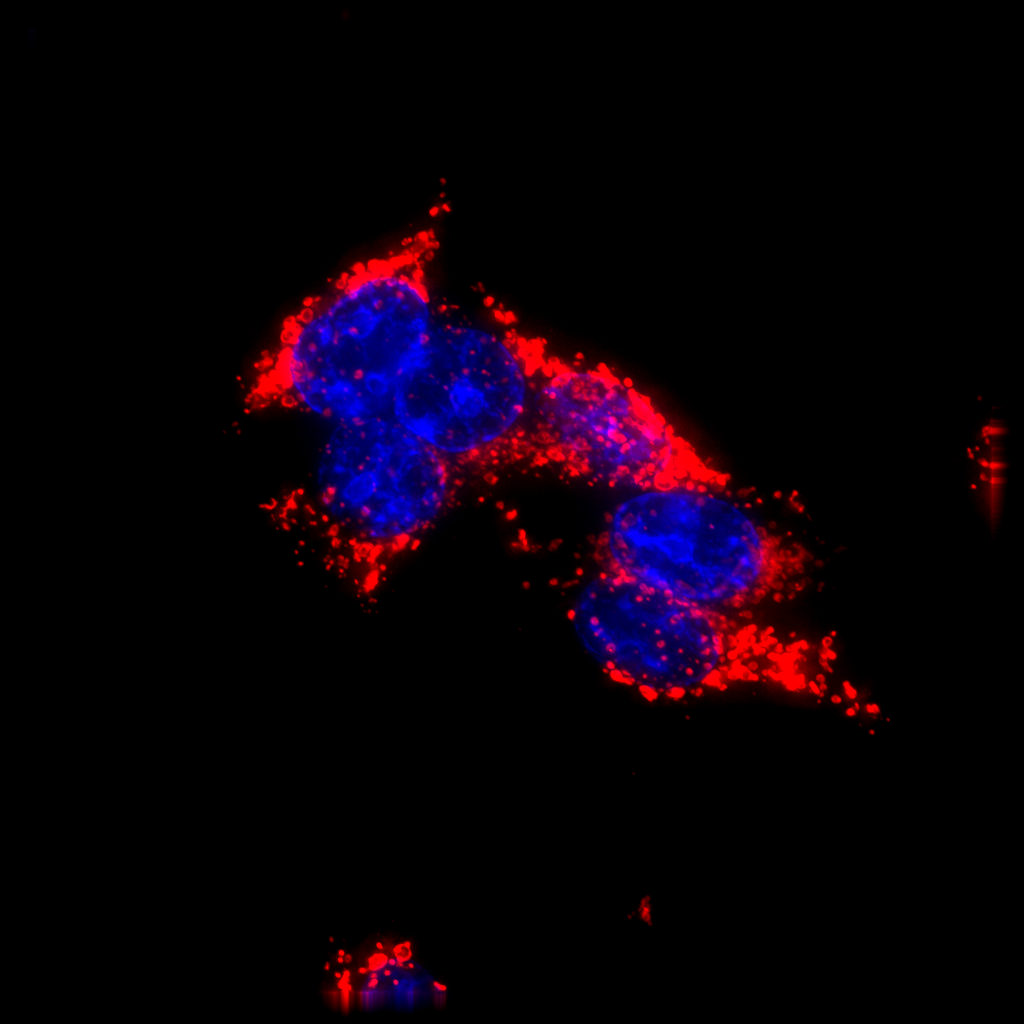

Supplement: Supplementary file 6 — Source data Fig. 1 [file 44318_2026_791_MOESM6_ESM.zip › Figure 1/1A/Figure1A_GNPTABKO.tif]

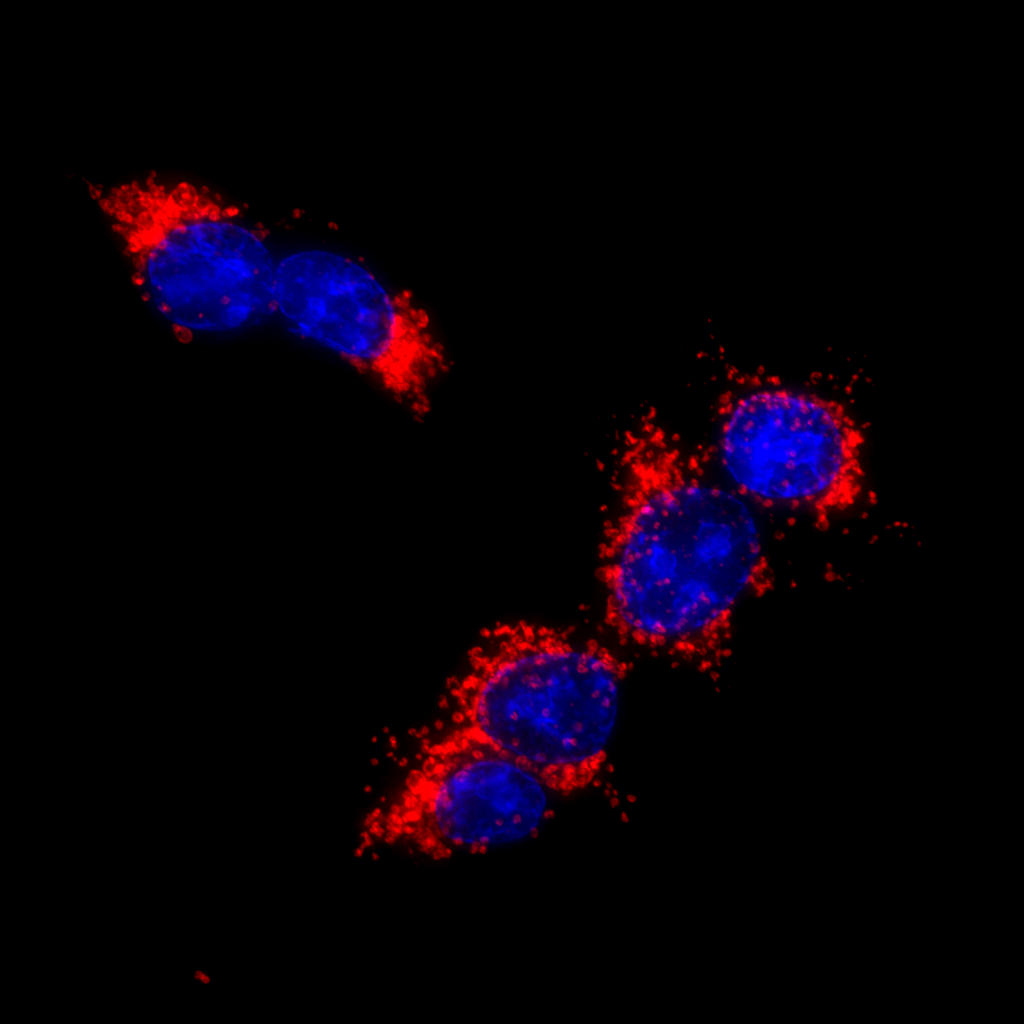

Supplement: Supplementary file 6 — Source data Fig. 1 [file 44318_2026_791_MOESM6_ESM.zip › Figure 1/1A/Figure1A_LYSETKO.tif]

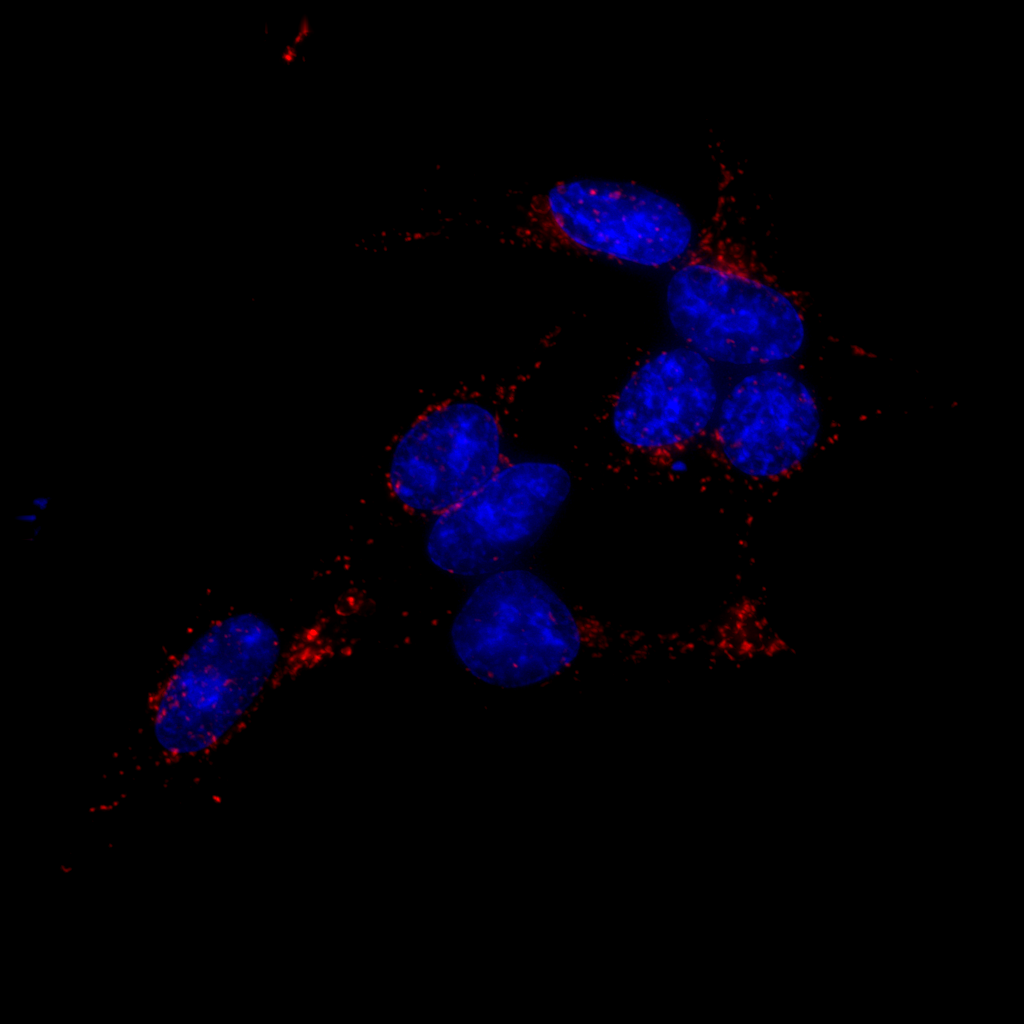

Supplement: Supplementary file 6 — Source data Fig. 1 [file 44318_2026_791_MOESM6_ESM.zip › Figure 1/1A/Figure1A_WT.tif]

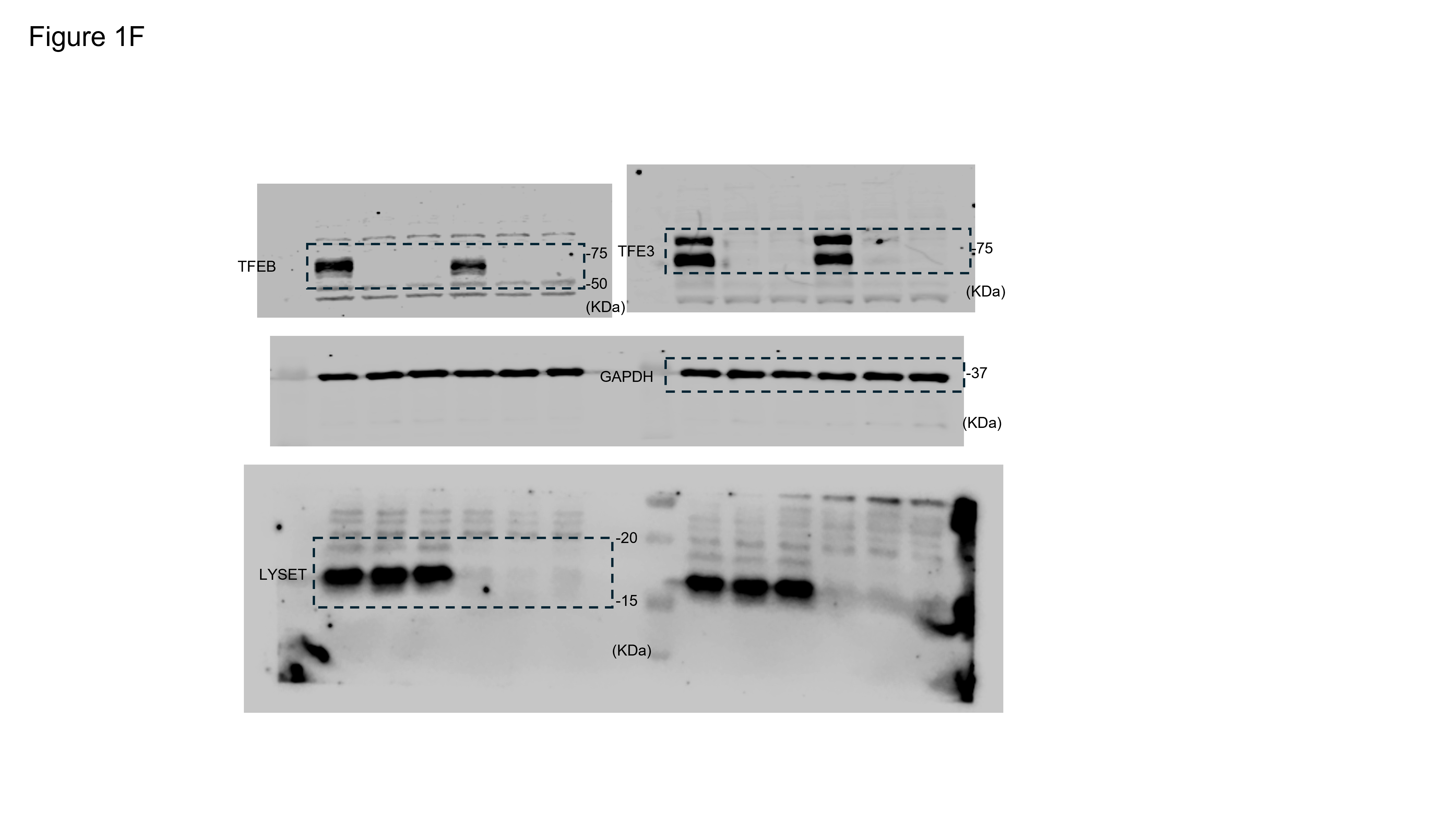

Supplement: Supplementary file 6 — Source data Fig. 1 [file 44318_2026_791_MOESM6_ESM.zip › Figure 1/1F/Figure1F_western.tif]

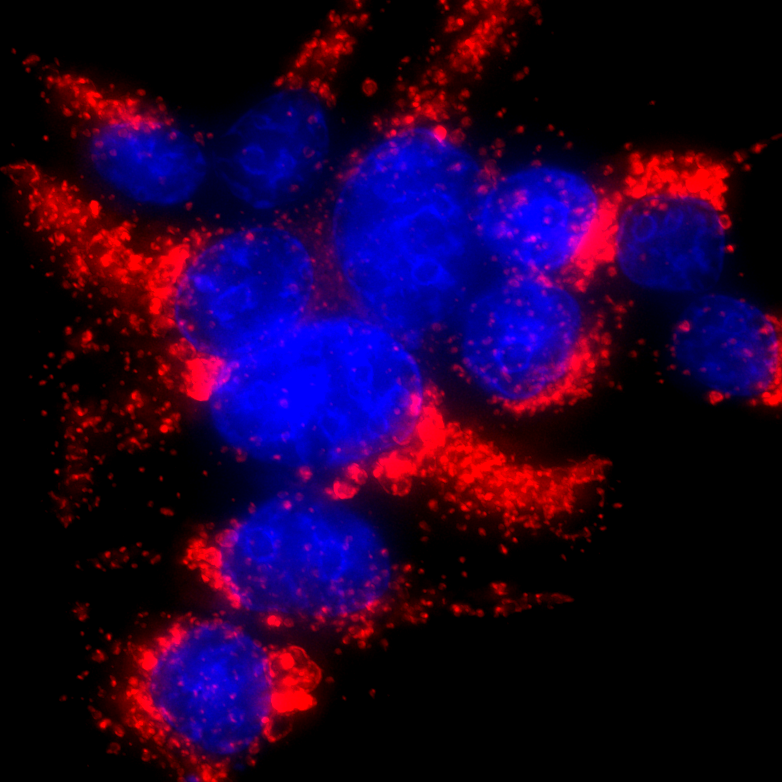

Supplement: Supplementary file 6 — Source data Fig. 1 [file 44318_2026_791_MOESM6_ESM.zip › Figure 1/1I-J/Figure1I_sgLYSET.tif]

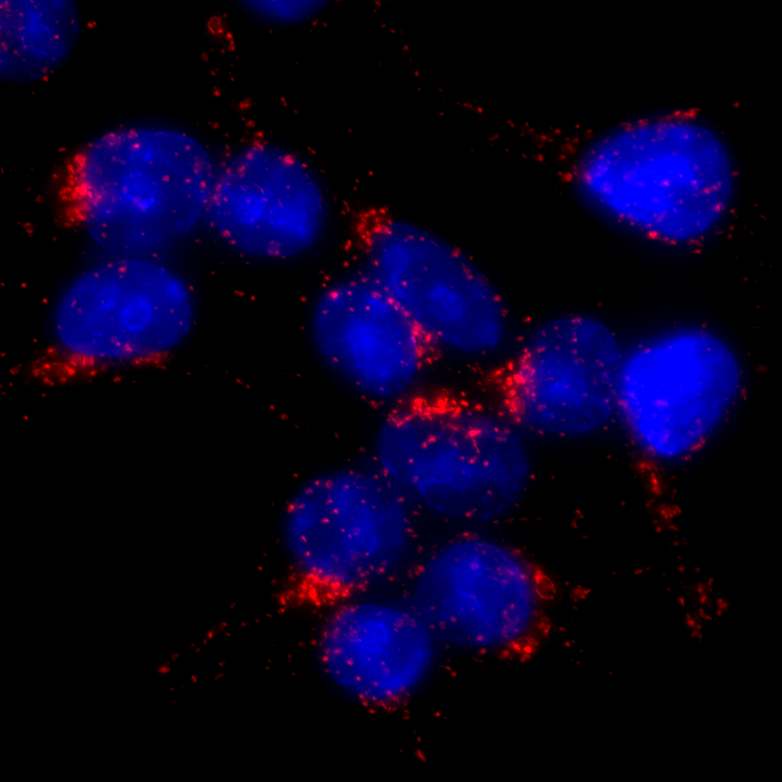

Supplement: Supplementary file 6 — Source data Fig. 1 [file 44318_2026_791_MOESM6_ESM.zip › Figure 1/1I-J/Figure1I_TFEBTFE3DKO59.tif]

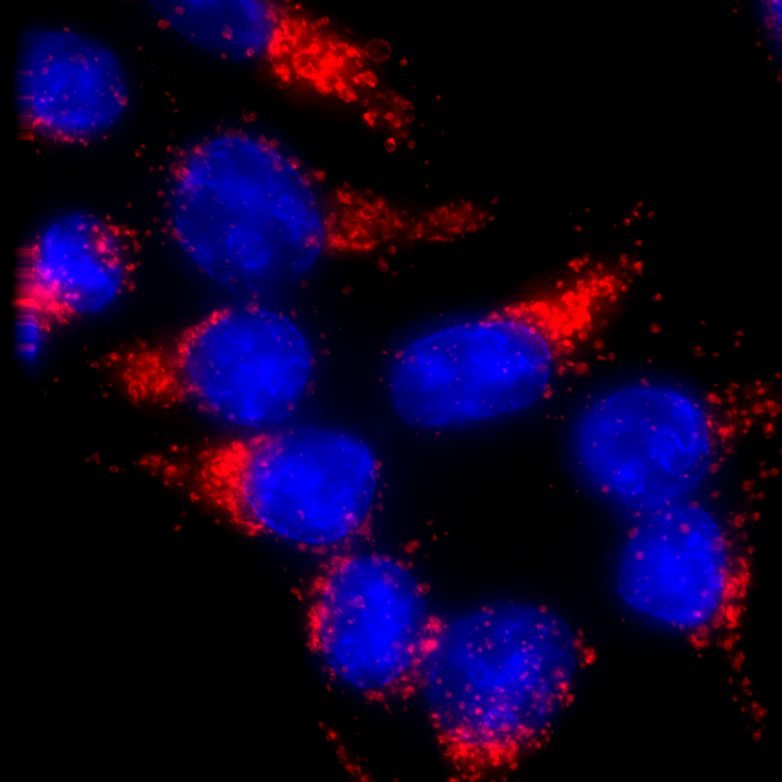

Supplement: Supplementary file 6 — Source data Fig. 1 [file 44318_2026_791_MOESM6_ESM.zip › Figure 1/1I-J/Figure1I_TFEBTFE3DKO59sgLYSET.tif]

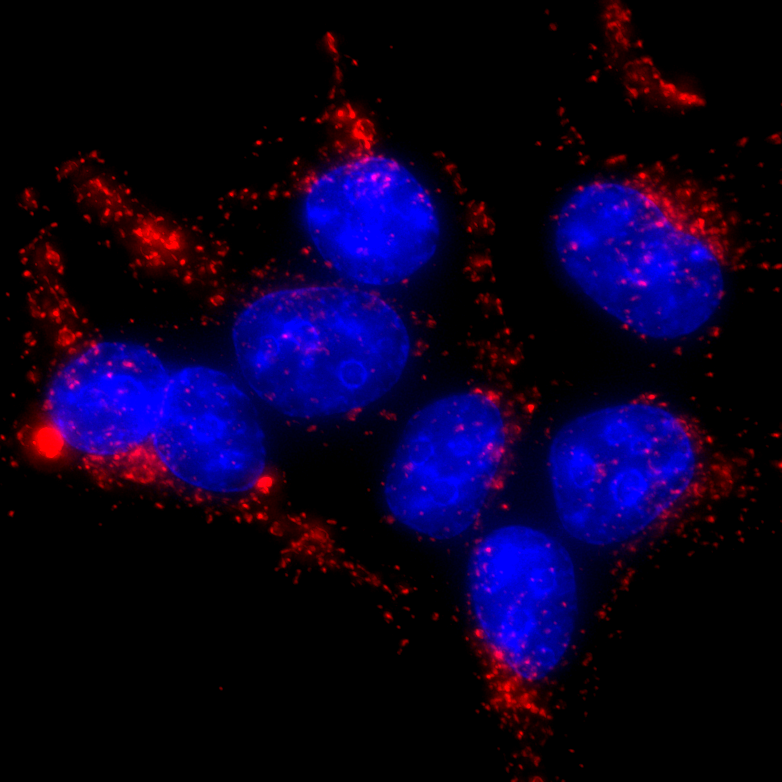

Supplement: Supplementary file 6 — Source data Fig. 1 [file 44318_2026_791_MOESM6_ESM.zip › Figure 1/1I-J/Figure1I_WT.tif]

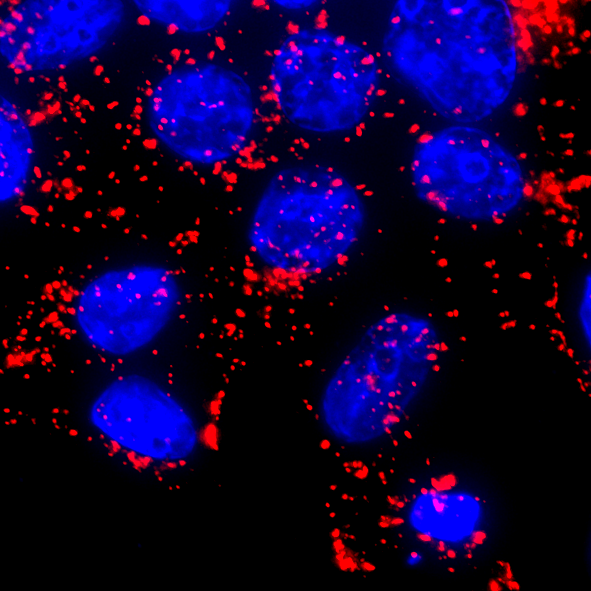

Supplement: Supplementary file 7 — Source data Fig. 2 [file 44318_2026_791_MOESM7_ESM.zip › Figure 2/2C/Figure2C_After_3rd_round_sorting.tif]

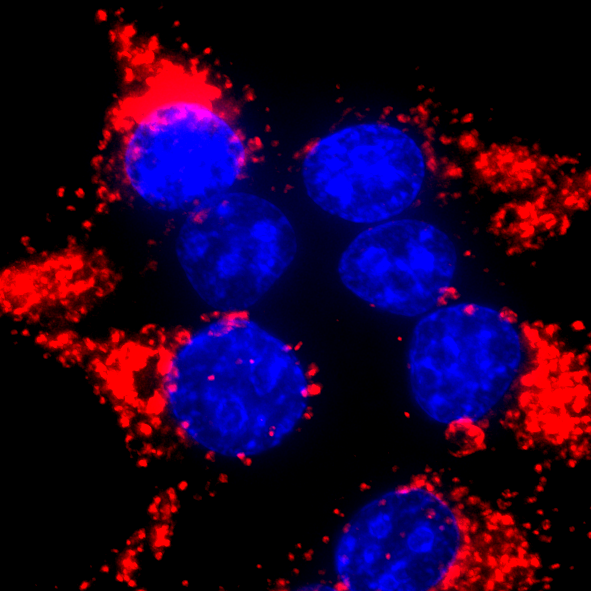

Supplement: Supplementary file 7 — Source data Fig. 2 [file 44318_2026_791_MOESM7_ESM.zip › Figure 2/2C/Figure2C_LYSETKO.tif]

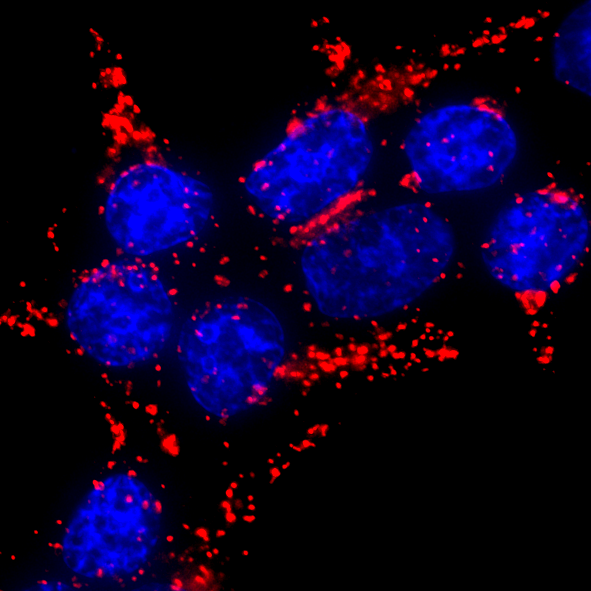

Supplement: Supplementary file 7 — Source data Fig. 2 [file 44318_2026_791_MOESM7_ESM.zip › Figure 2/2C/Figure2C_WT.tif]

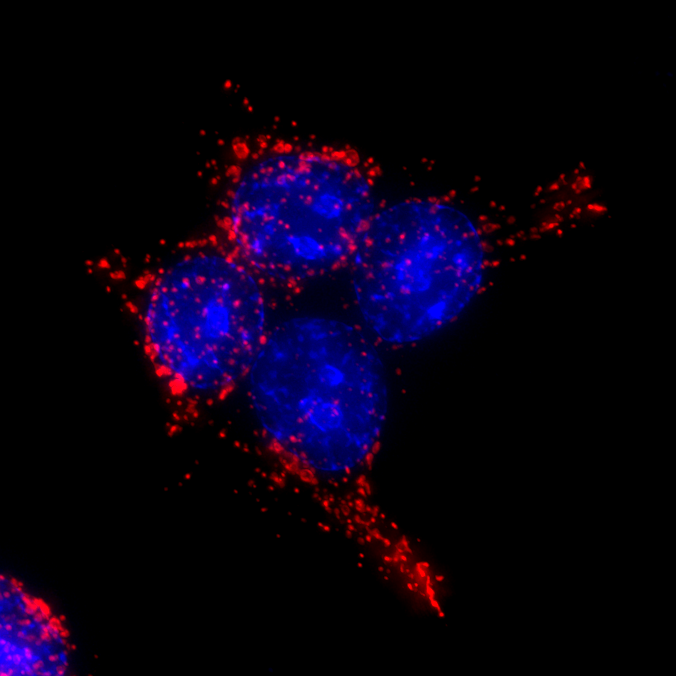

Supplement: Supplementary file 8 — Source data Fig. 3 [file 44318_2026_791_MOESM8_ESM.zip › Figure 3/3A-B/Figure3A_LYSETFAR1DKO.tif]

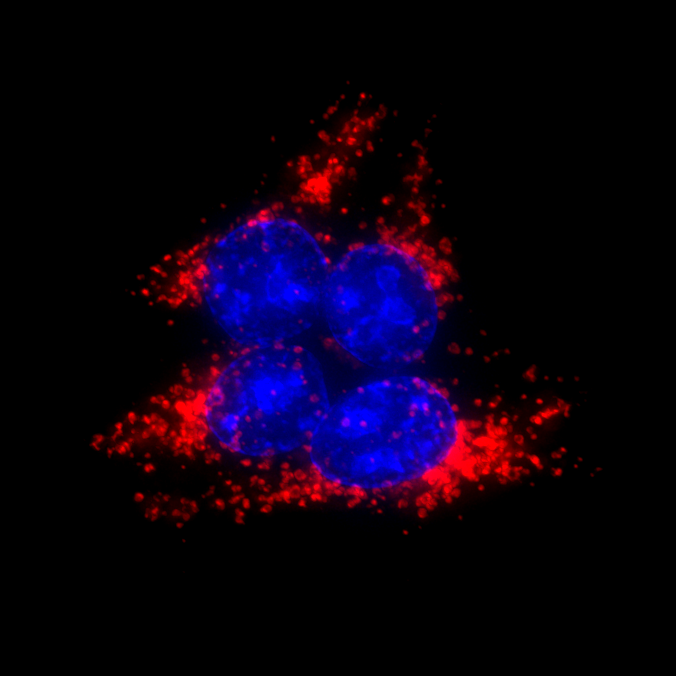

Supplement: Supplementary file 8 — Source data Fig. 3 [file 44318_2026_791_MOESM8_ESM.zip › Figure 3/3A-B/Figure3A_LYSETFAR1DKO_3FLAGFAR1.tif]

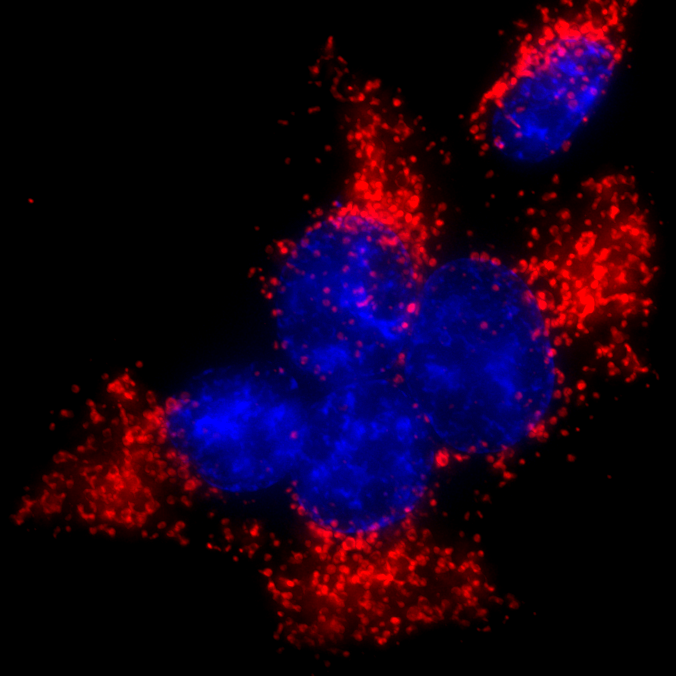

Supplement: Supplementary file 8 — Source data Fig. 3 [file 44318_2026_791_MOESM8_ESM.zip › Figure 3/3A-B/Figure3A_LYSETKO.tif]

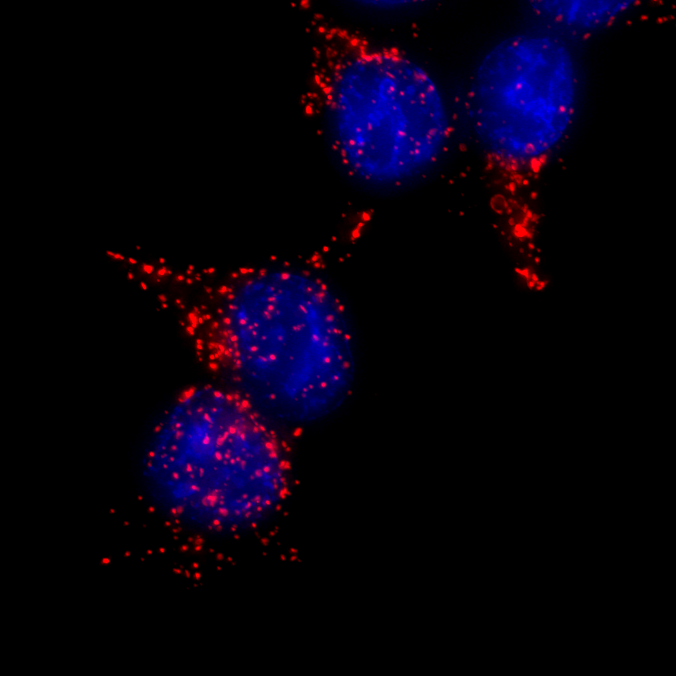

Supplement: Supplementary file 8 — Source data Fig. 3 [file 44318_2026_791_MOESM8_ESM.zip › Figure 3/3A-B/Figure3A_WT.tif]

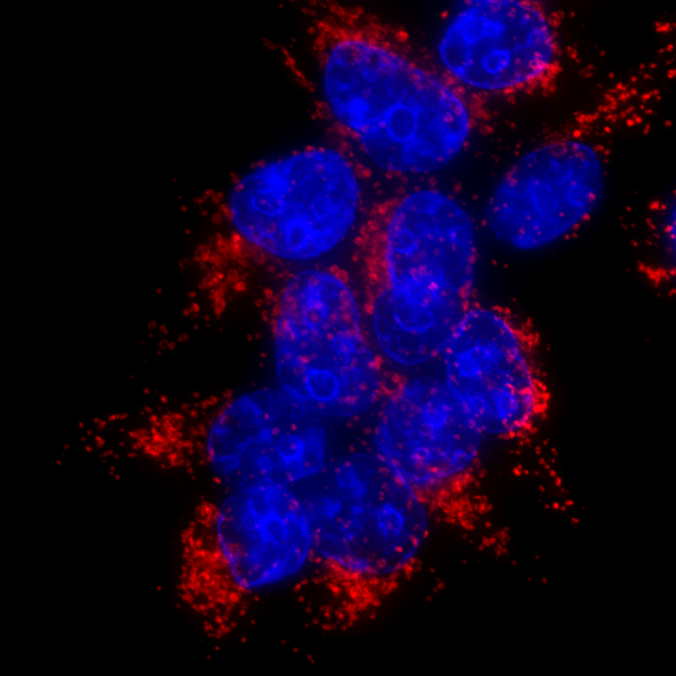

Supplement: Supplementary file 8 — Source data Fig. 3 [file 44318_2026_791_MOESM8_ESM.zip › Figure 3/3C-D/Figure3C_GNPATBFAR1DKO_3FLAGFAR1.tif]

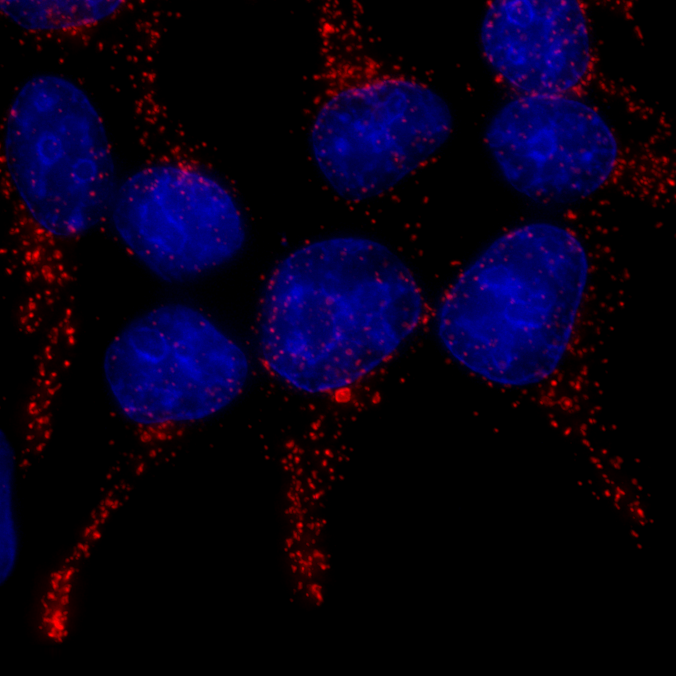

Supplement: Supplementary file 8 — Source data Fig. 3 [file 44318_2026_791_MOESM8_ESM.zip › Figure 3/3C-D/Figure3C_GNPTABFAR1DKO.tif]

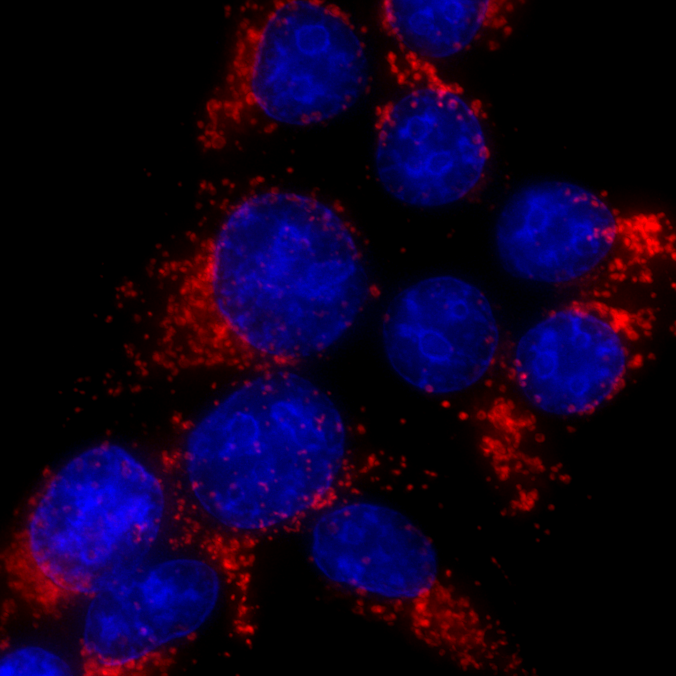

Supplement: Supplementary file 8 — Source data Fig. 3 [file 44318_2026_791_MOESM8_ESM.zip › Figure 3/3C-D/Figure3C_GNPTABKO.tif]

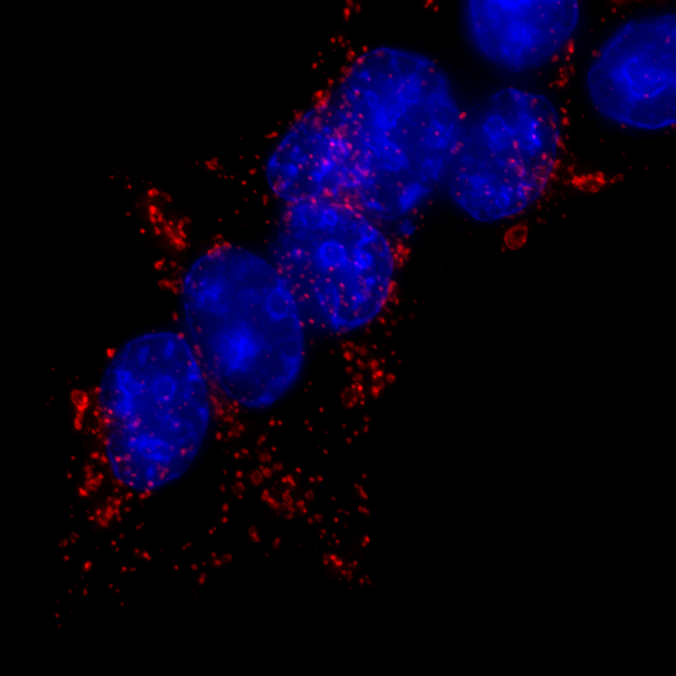

Supplement: Supplementary file 8 — Source data Fig. 3 [file 44318_2026_791_MOESM8_ESM.zip › Figure 3/3C-D/Figure3C_WT.tif]

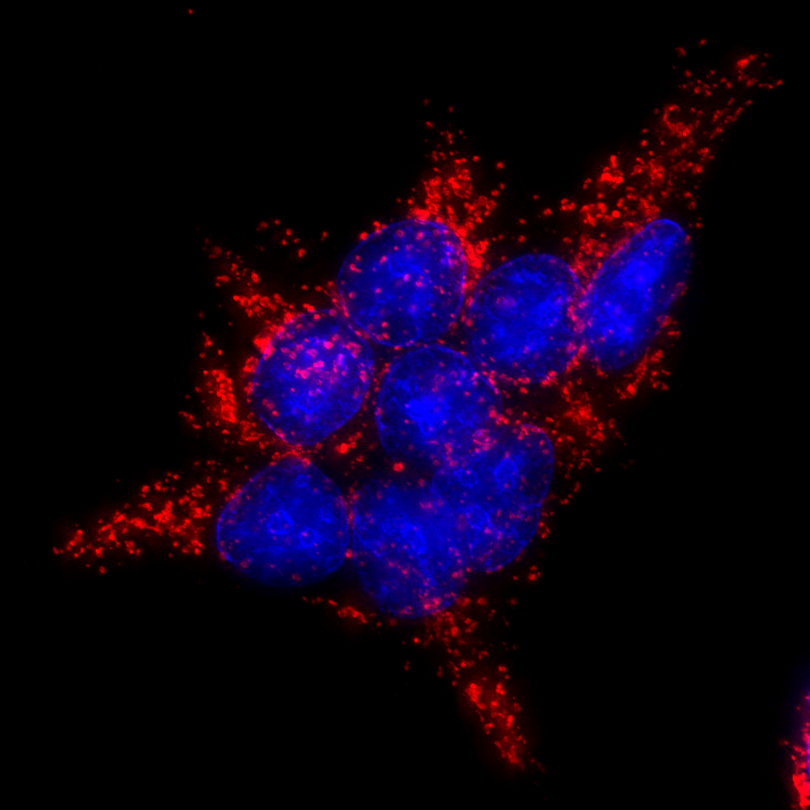

Supplement: Supplementary file 8 — Source data Fig. 3 [file 44318_2026_791_MOESM8_ESM.zip › Figure 3/3E-F/Figure3E_LYSETAGPSDKO.tif]

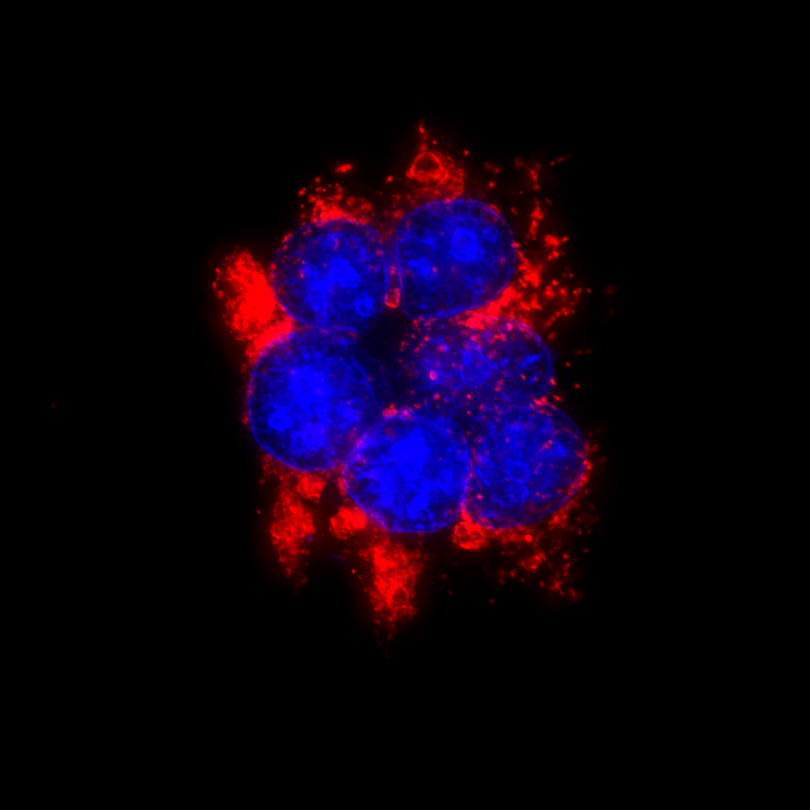

Supplement: Supplementary file 8 — Source data Fig. 3 [file 44318_2026_791_MOESM8_ESM.zip › Figure 3/3E-F/Figure3E_LYSETAGPSDKO_AGPS.tif]

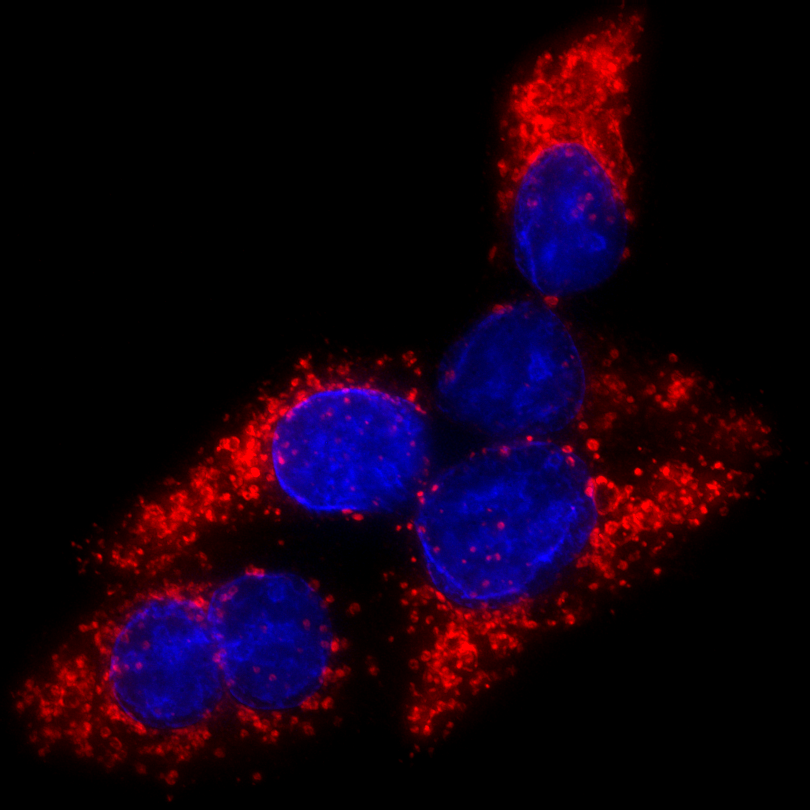

Supplement: Supplementary file 8 — Source data Fig. 3 [file 44318_2026_791_MOESM8_ESM.zip › Figure 3/3E-F/Figure3E_LYSETKO.tif]

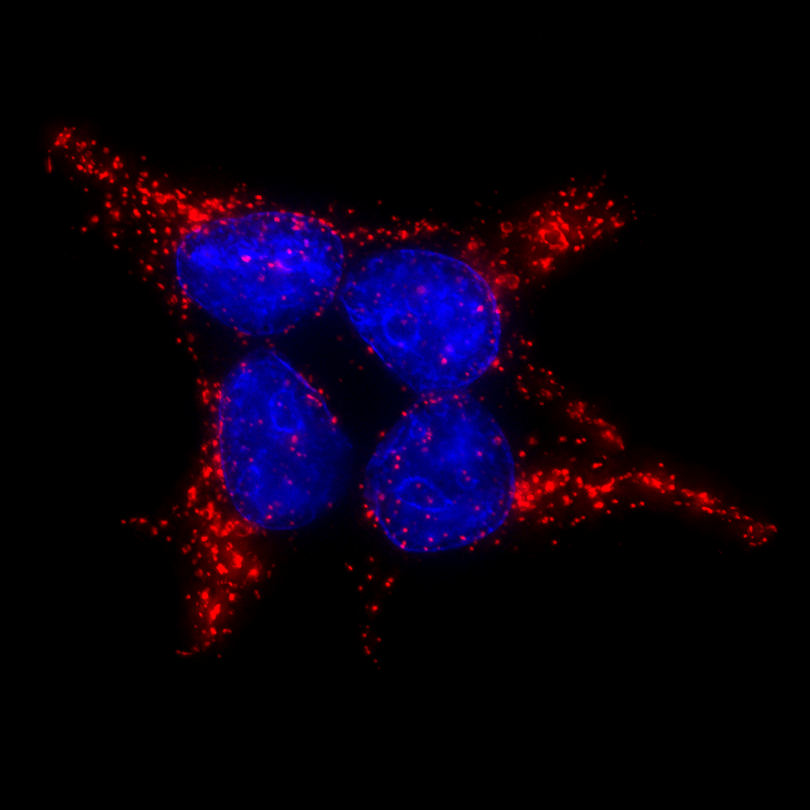

Supplement: Supplementary file 8 — Source data Fig. 3 [file 44318_2026_791_MOESM8_ESM.zip › Figure 3/3E-F/Figure3E_WT.tif]

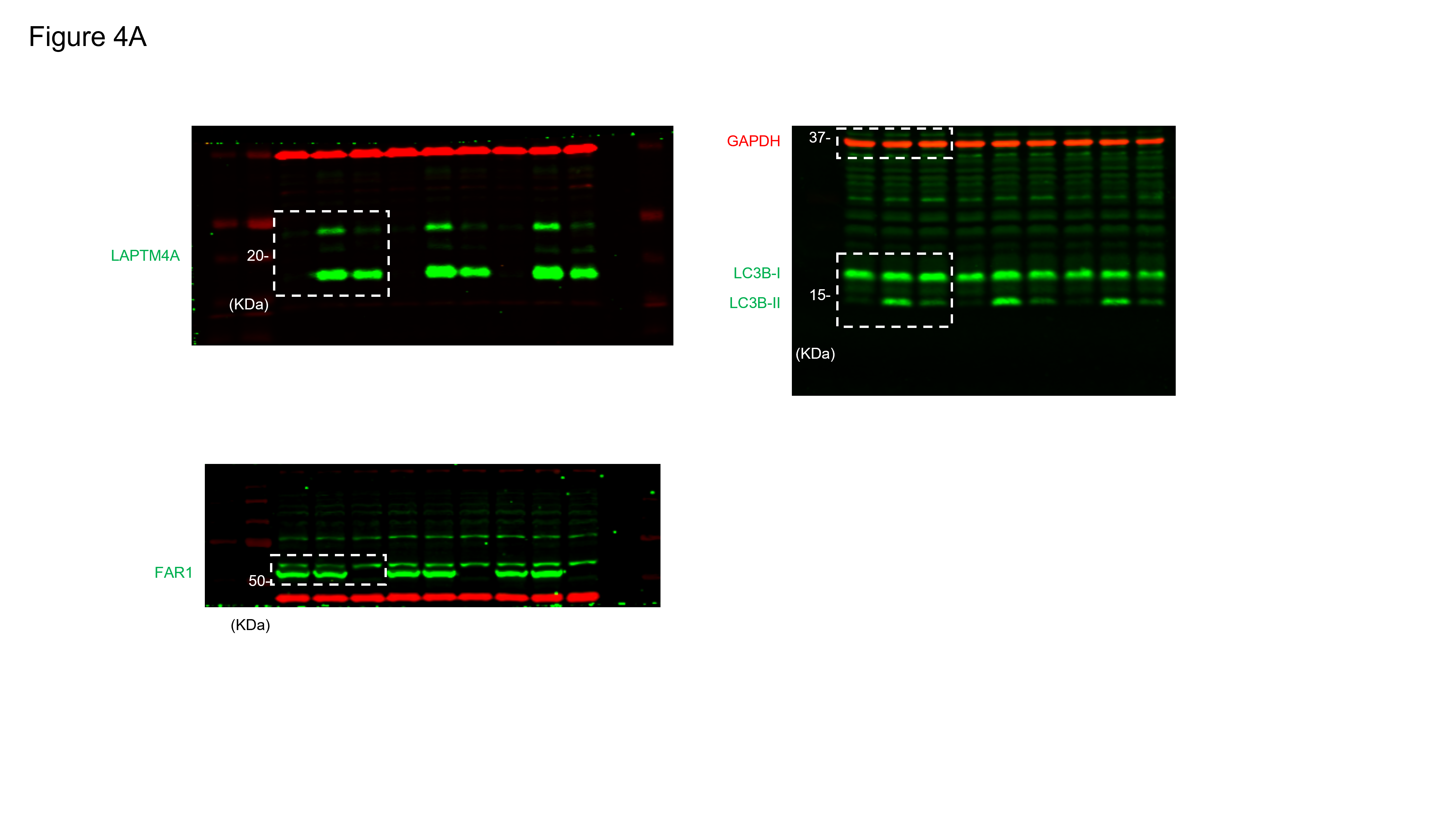

Supplement: Supplementary file 9 — Source data Fig. 4 [file 44318_2026_791_MOESM9_ESM.zip › Figure 4/4A-B/Figure4A_western.tif]

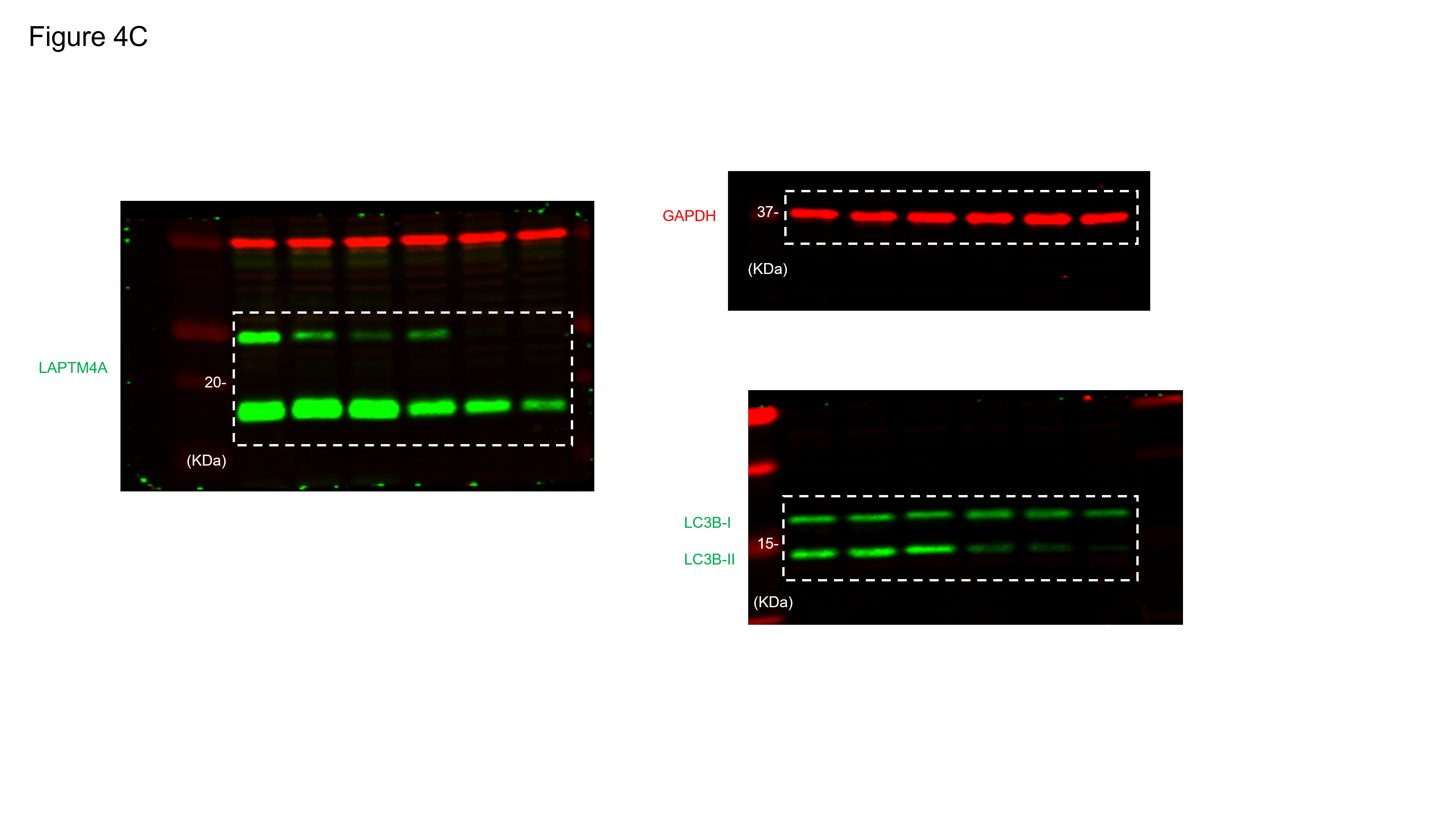

Supplement: Supplementary file 9 — Source data Fig. 4 [file 44318_2026_791_MOESM9_ESM.zip › Figure 4/4C-D/Figure4C_western.tif]

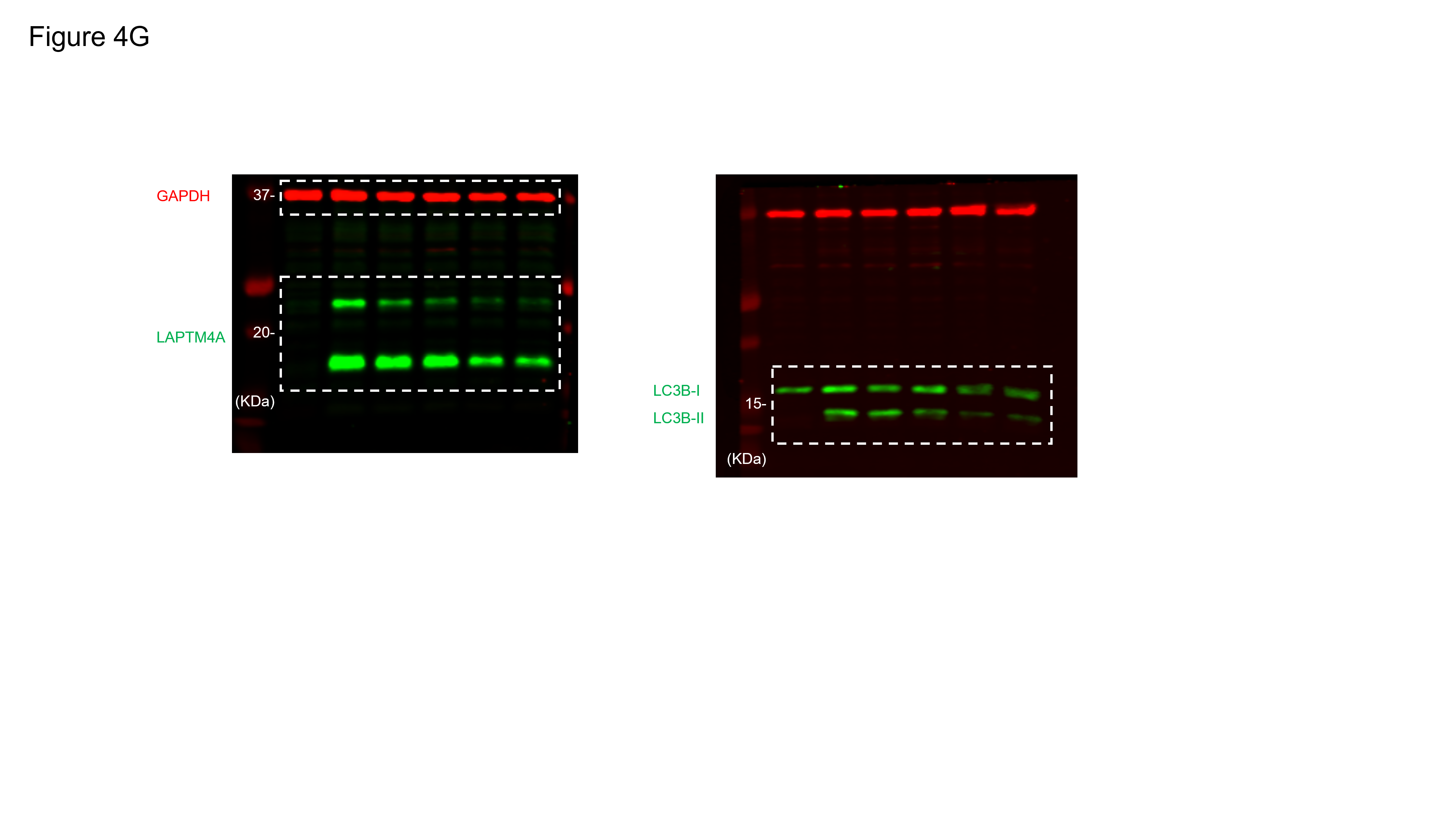

Supplement: Supplementary file 9 — Source data Fig. 4 [file 44318_2026_791_MOESM9_ESM.zip › Figure 4/4G-H/Figure4G_western.tif]

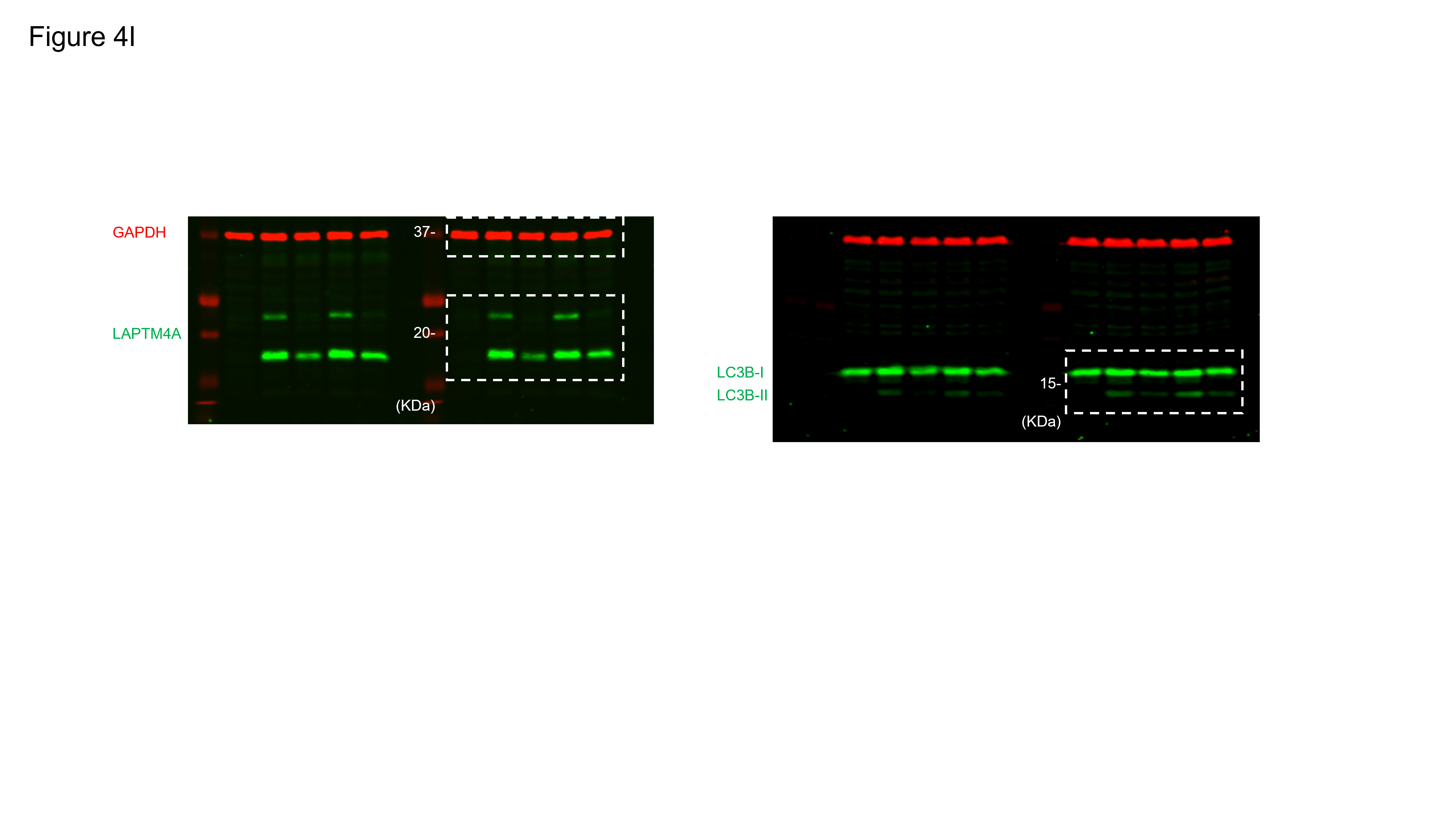

Supplement: Supplementary file 9 — Source data Fig. 4 [file 44318_2026_791_MOESM9_ESM.zip › Figure 4/4I-J/Figure4I_western.tif]

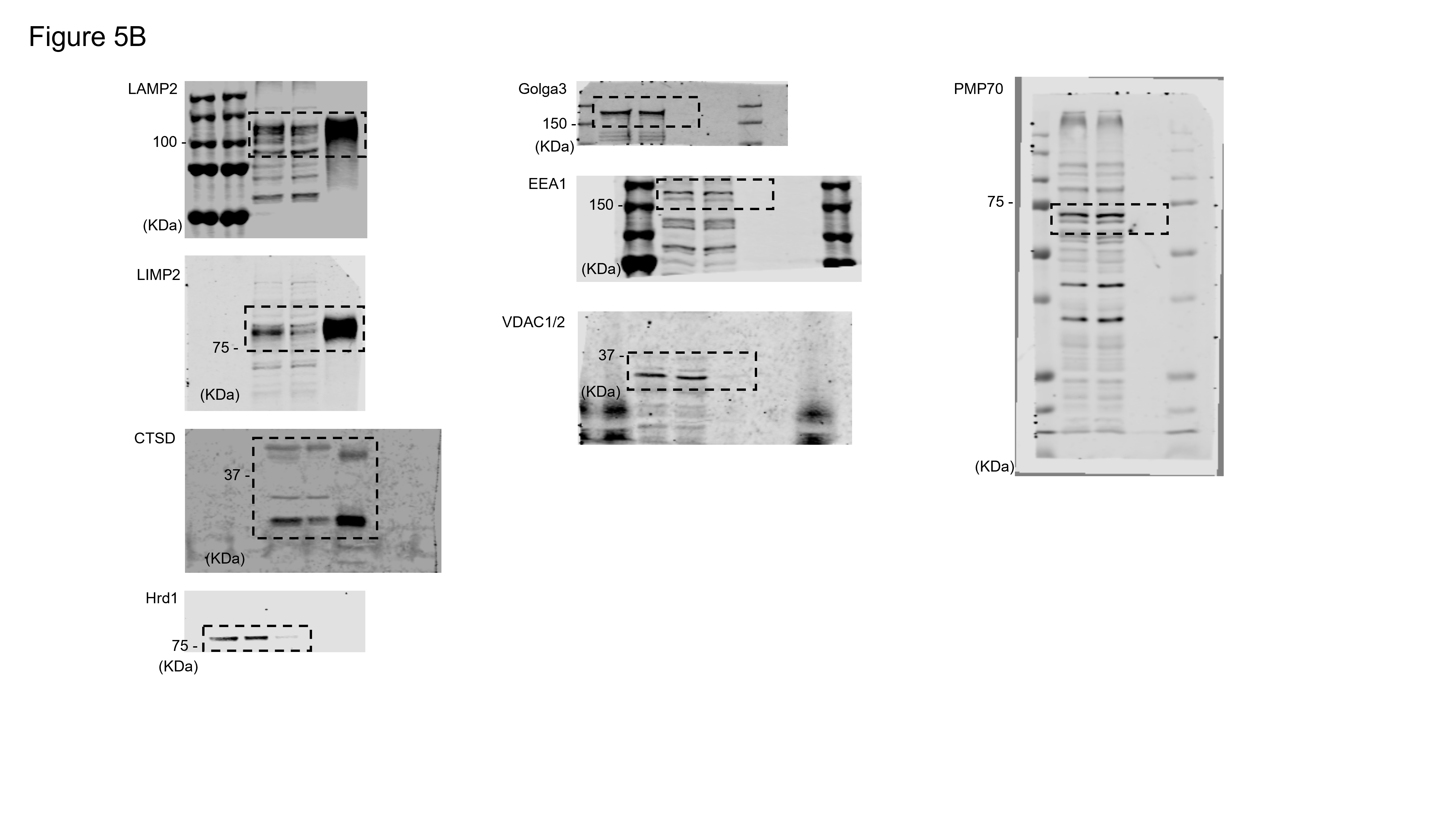

Supplement: Supplementary file 10 — Source data Fig. 5 [file 44318_2026_791_MOESM10_ESM.zip › Figure 5/5B/Figure5B_western.tif]

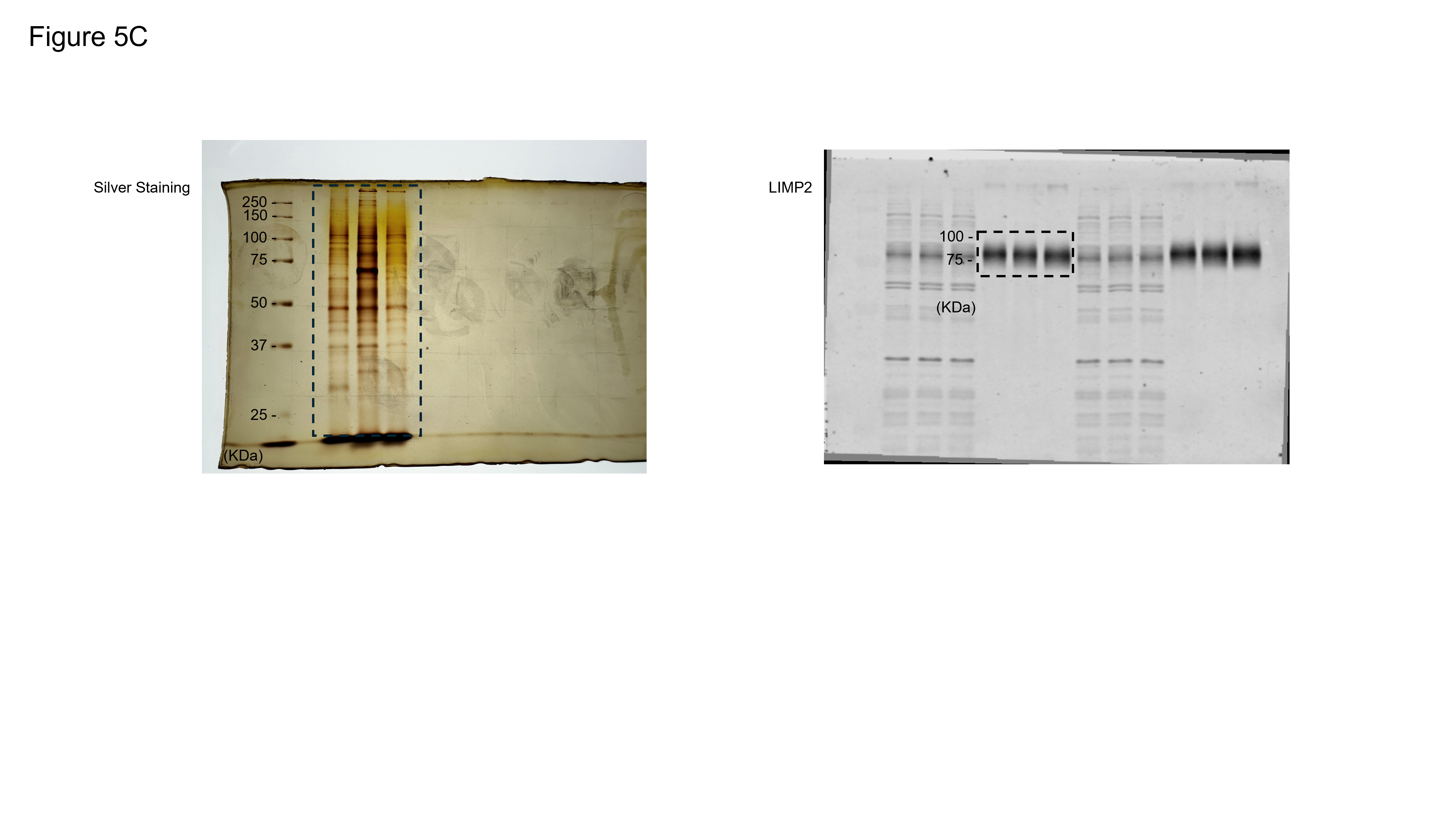

Supplement: Supplementary file 10 — Source data Fig. 5 [file 44318_2026_791_MOESM10_ESM.zip › Figure 5/5C/Figure5C_silverstaining_and_western.tif]

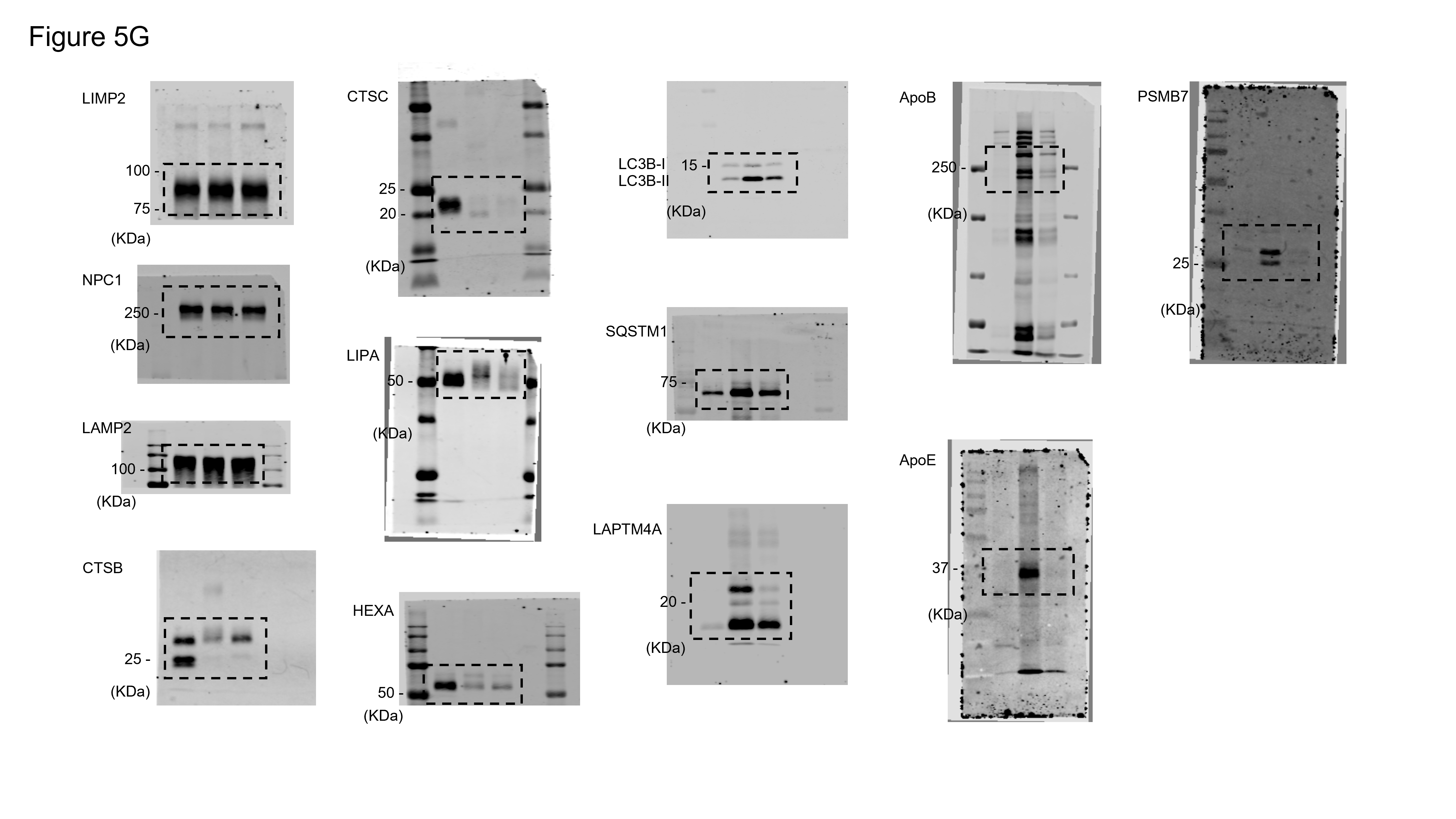

Supplement: Supplementary file 10 — Source data Fig. 5 [file 44318_2026_791_MOESM10_ESM.zip › Figure 5/5G/Figure5G_western.tif]

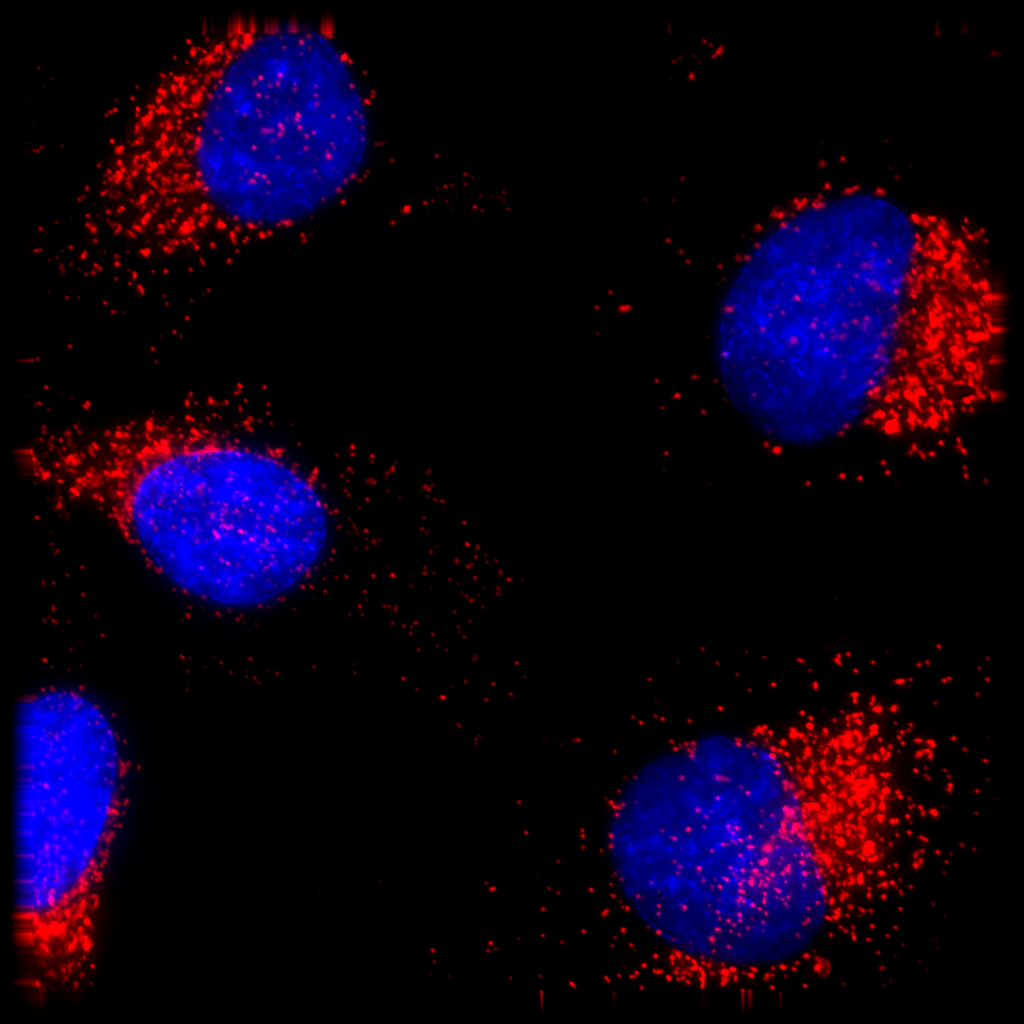

Supplement: Supplementary file 11 — Source data Fig. 6 [file 44318_2026_791_MOESM11_ESM.zip › Figure 6/6G-H/Figure6G_25uMHDG_LAMP2.tif]

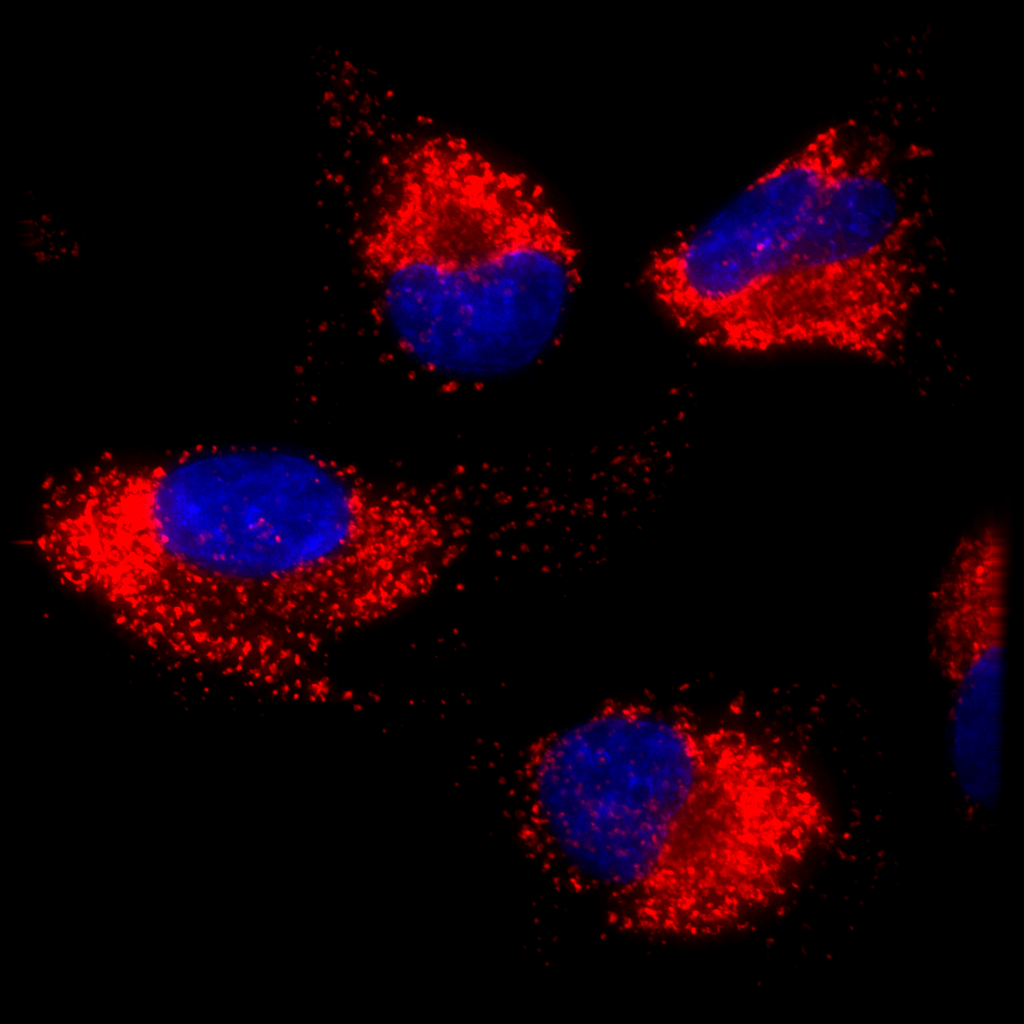

Supplement: Supplementary file 11 — Source data Fig. 6 [file 44318_2026_791_MOESM11_ESM.zip › Figure 6/6G-H/Figure6G_50uMHDG_LAMP2.tif]

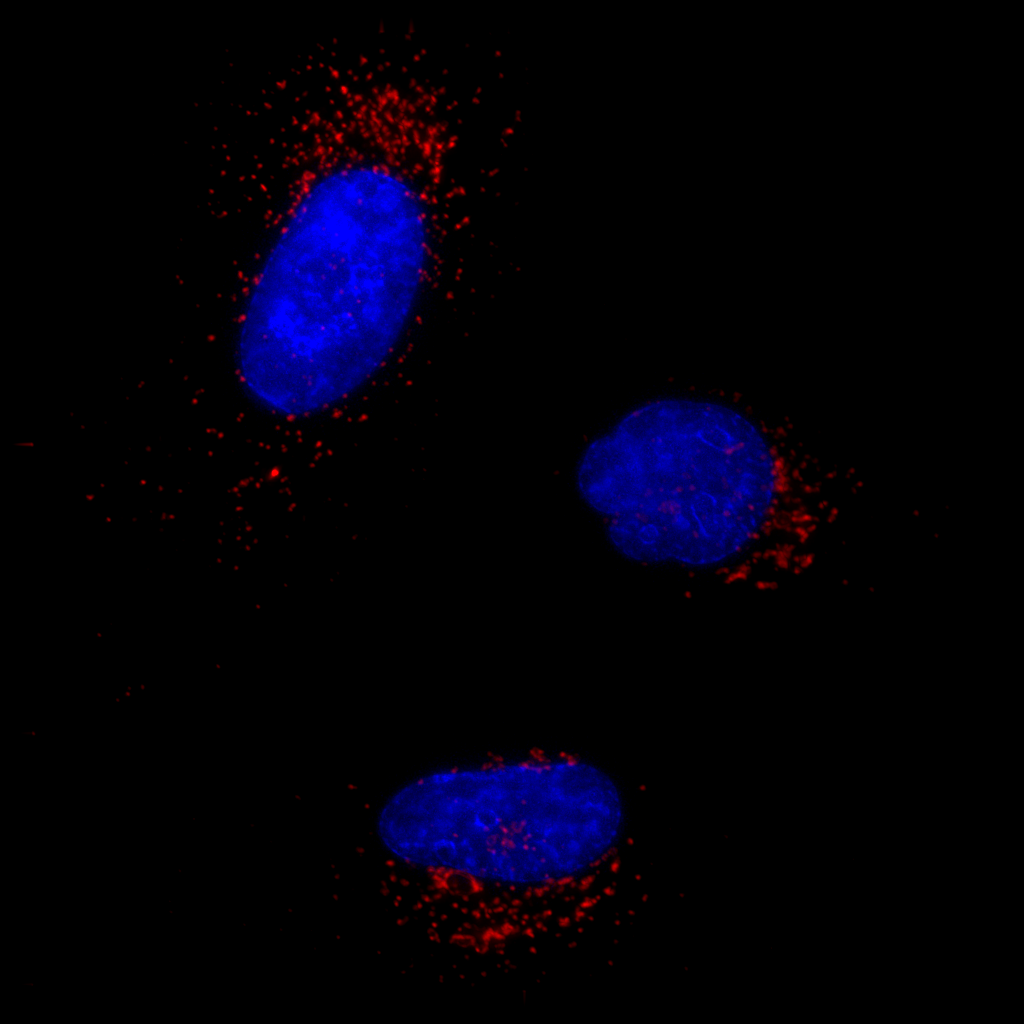

Supplement: Supplementary file 11 — Source data Fig. 6 [file 44318_2026_791_MOESM11_ESM.zip › Figure 6/6G-H/Figure6G_EtOH_LAMP2.tif]

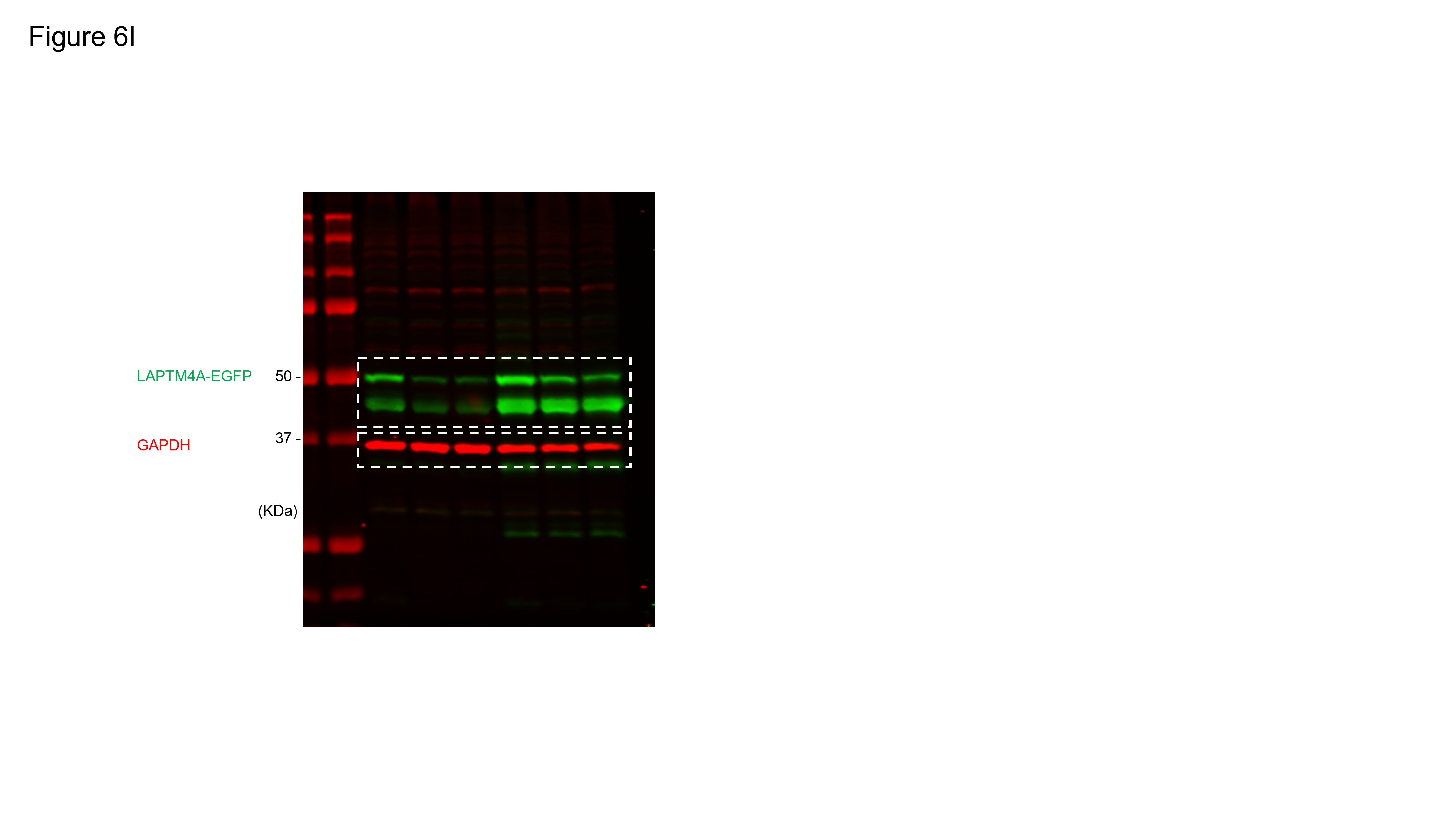

Supplement: Supplementary file 11 — Source data Fig. 6 [file 44318_2026_791_MOESM11_ESM.zip › Figure 6/6I/Figure6I_western.tif]

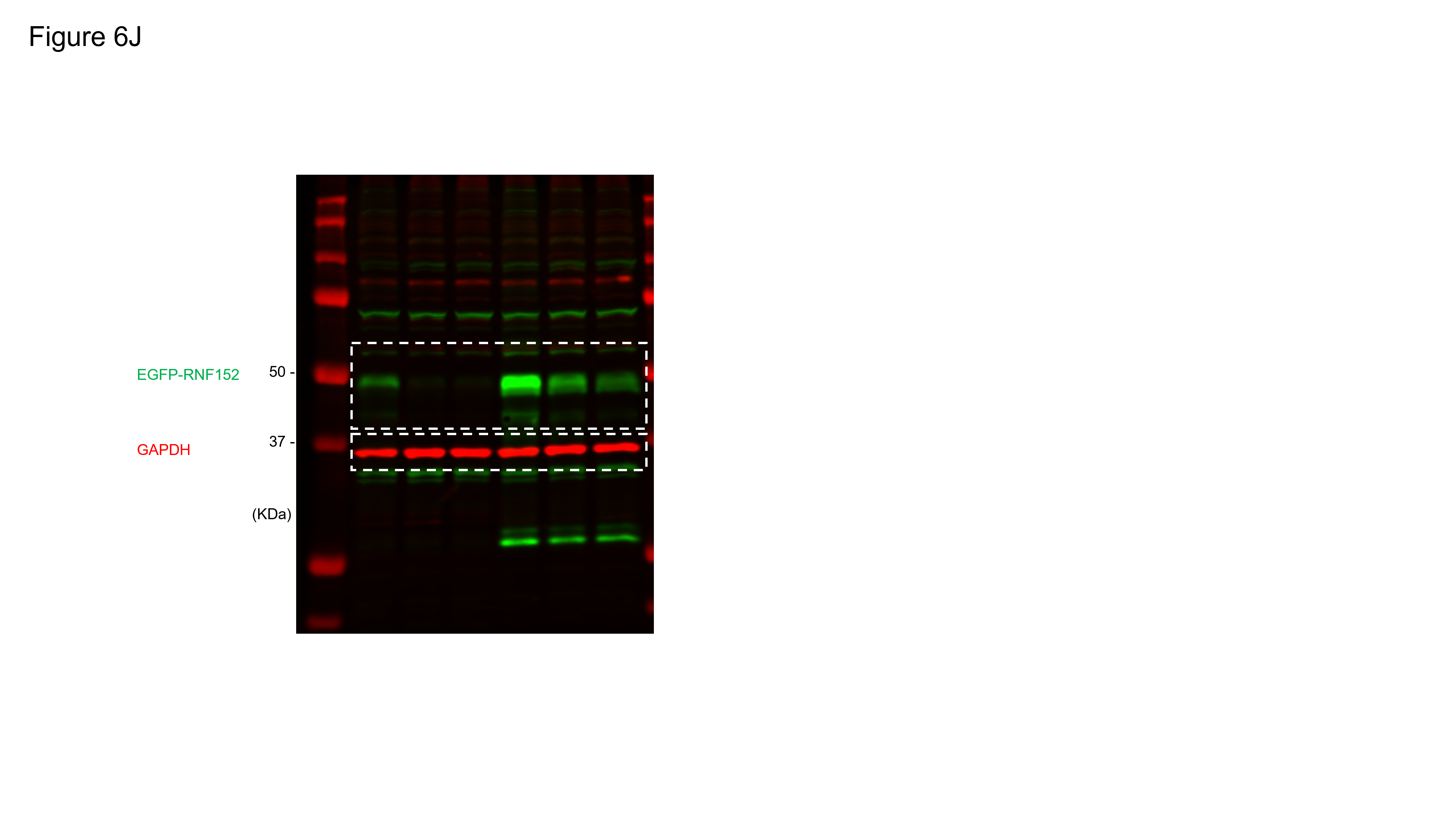

Supplement: Supplementary file 11 — Source data Fig. 6 [file 44318_2026_791_MOESM11_ESM.zip › Figure 6/6J/Figure6J_western.tif]

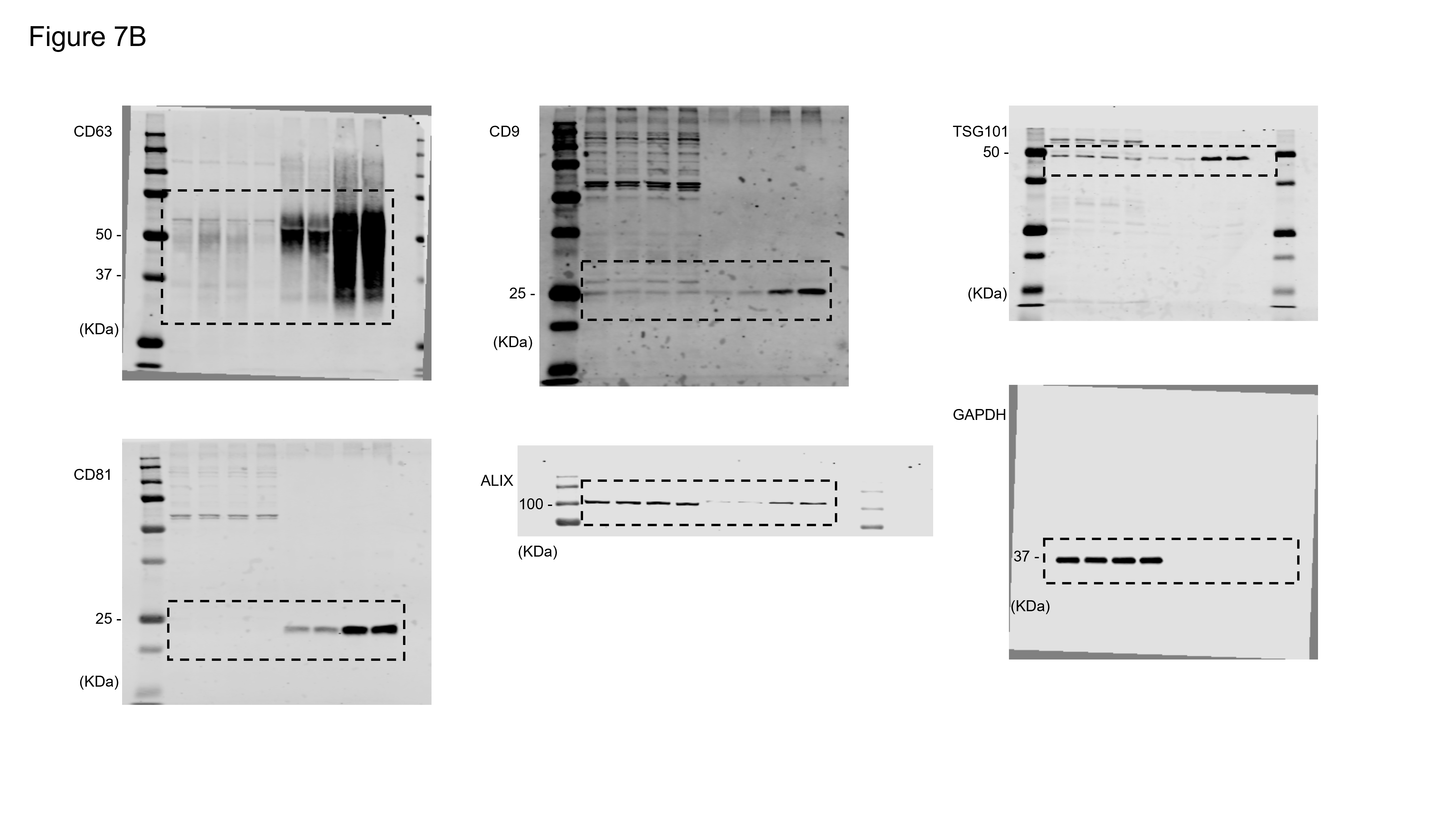

Supplement: Supplementary file 12 — Source data Fig. 7 [file 44318_2026_791_MOESM12_ESM.zip › Figure 7/7B/Figure7B_western.tif]

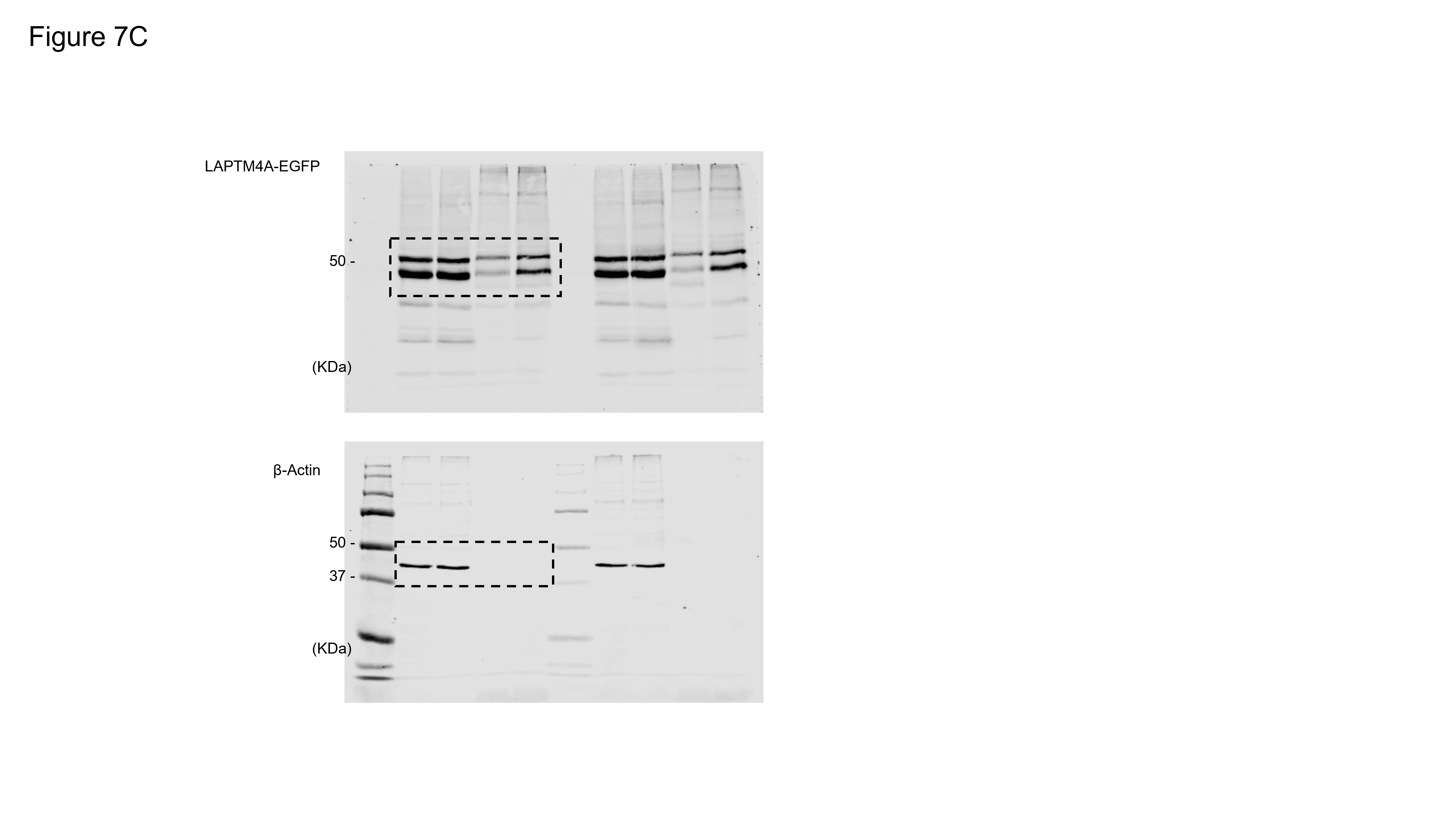

Supplement: Supplementary file 12 — Source data Fig. 7 [file 44318_2026_791_MOESM12_ESM.zip › Figure 7/7C/Figure7C_western.tif]

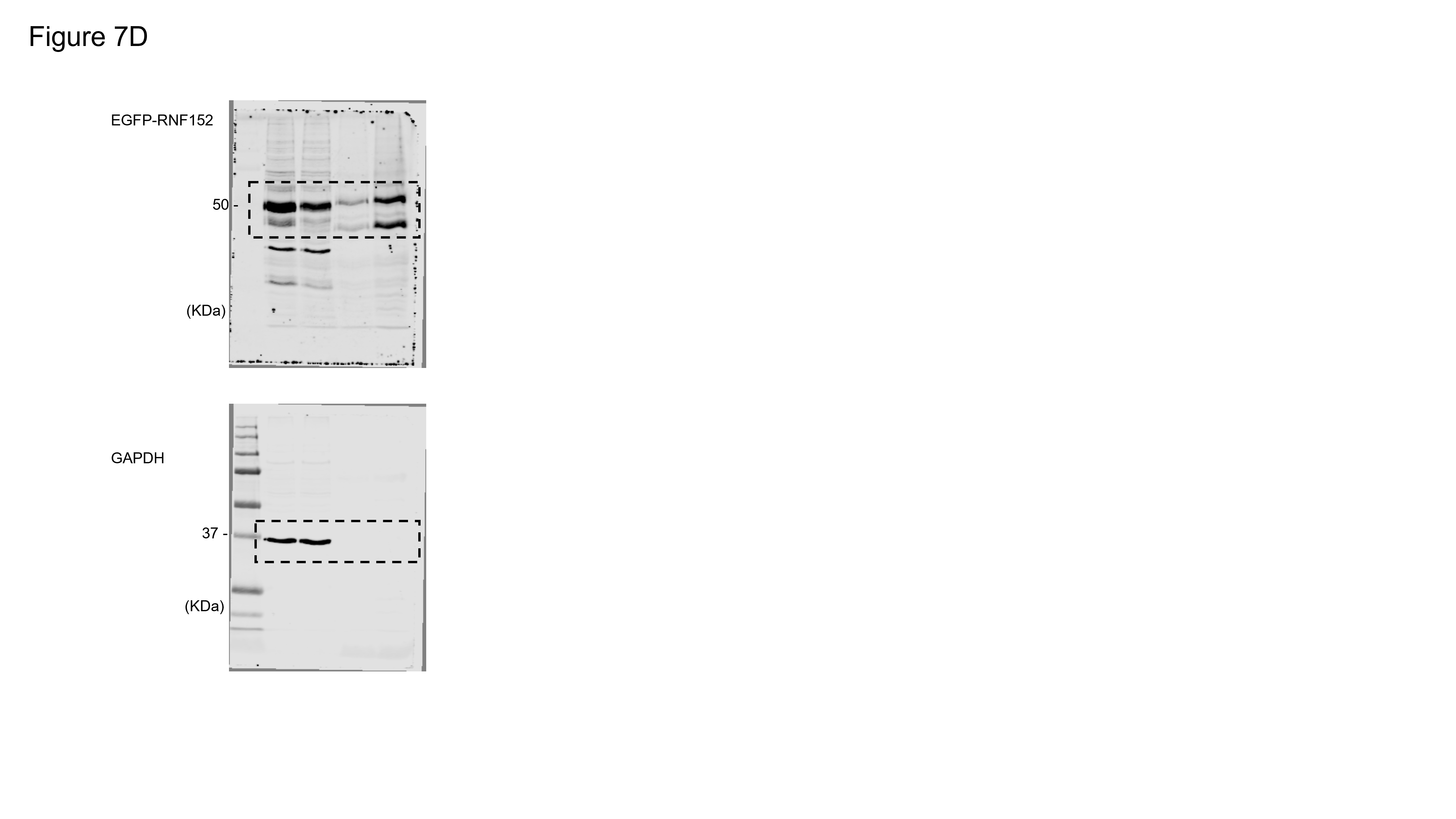

Supplement: Supplementary file 12 — Source data Fig. 7 [file 44318_2026_791_MOESM12_ESM.zip › Figure 7/7D/Figure7D_western.tif]

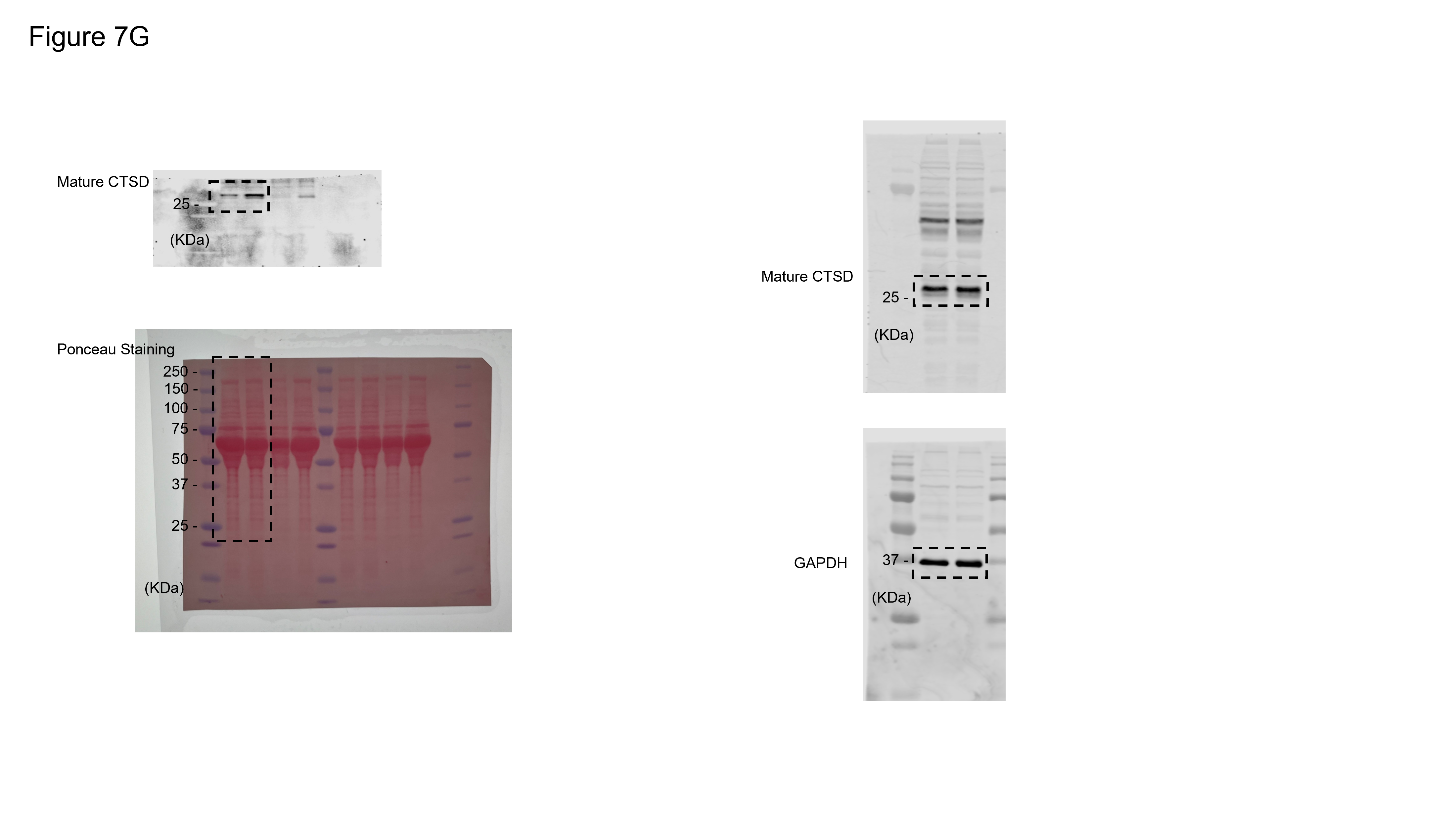

Supplement: Supplementary file 12 — Source data Fig. 7 [file 44318_2026_791_MOESM12_ESM.zip › Figure 7/7G-H/Figure7G_western.tif]

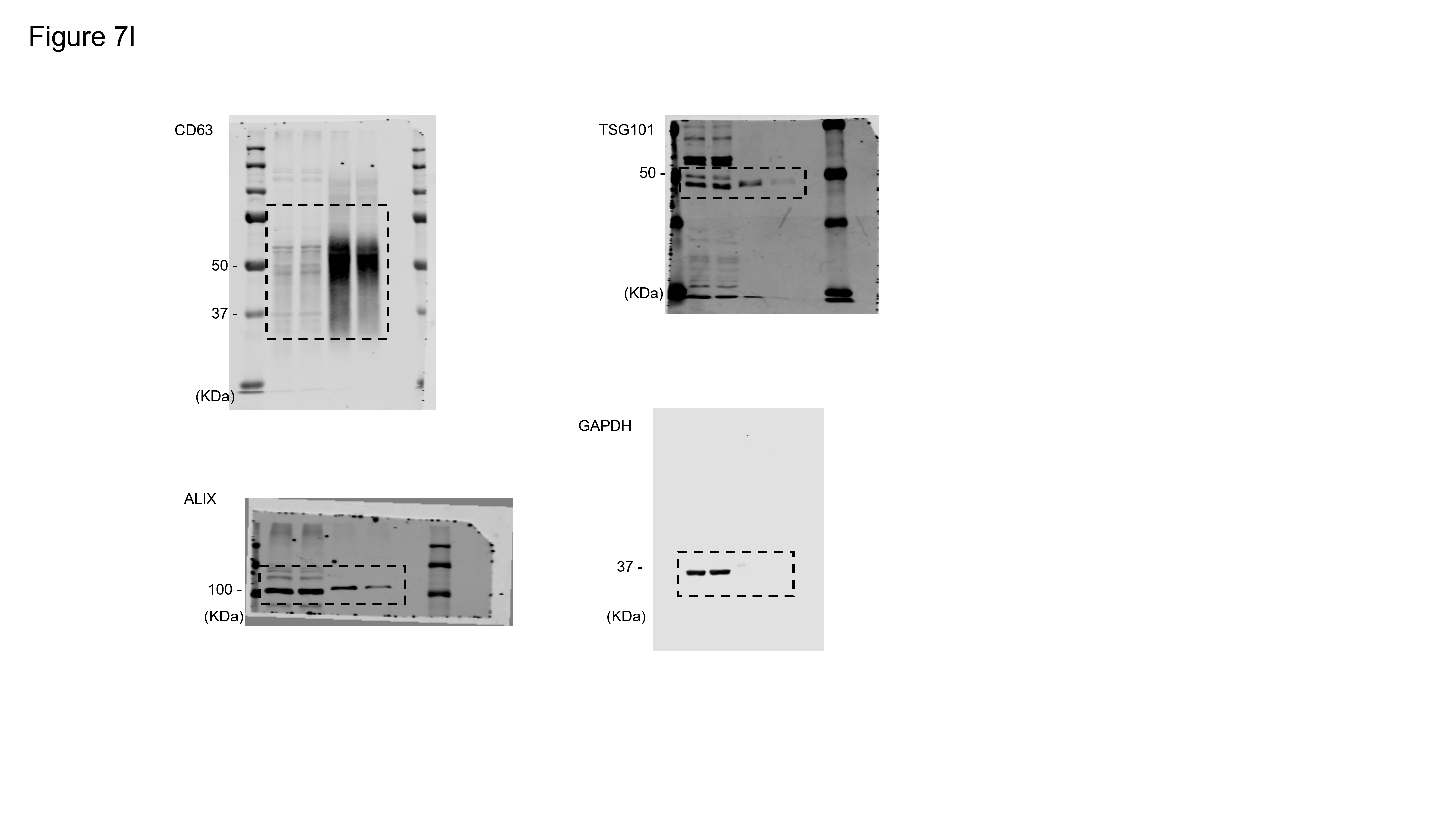

Supplement: Supplementary file 12 — Source data Fig. 7 [file 44318_2026_791_MOESM12_ESM.zip › Figure 7/7I/Figure7I_western.tif]

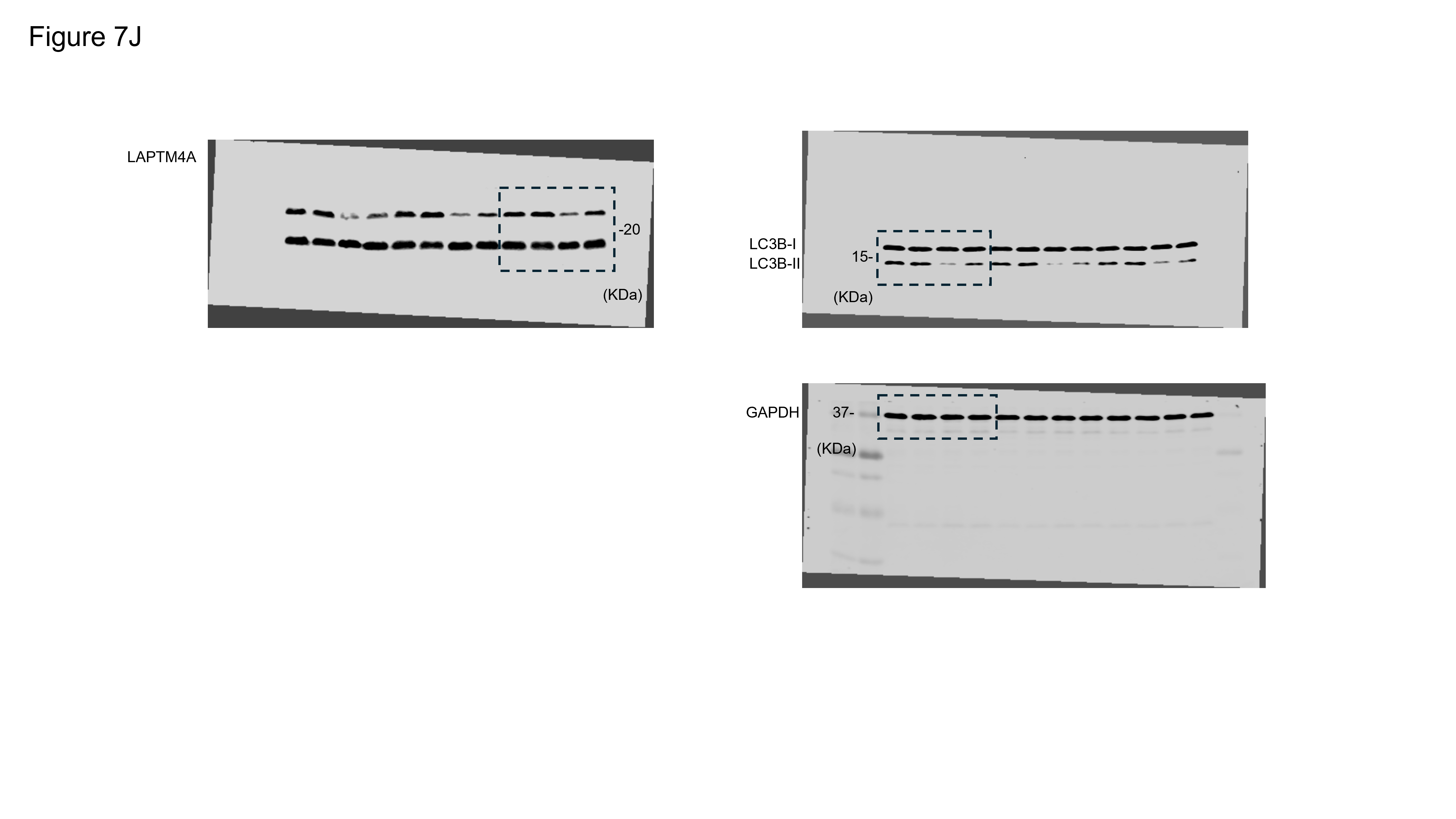

Supplement: Supplementary file 12 — Source data Fig. 7 [file 44318_2026_791_MOESM12_ESM.zip › Figure 7/7J-L/Figure7J_western.tif]

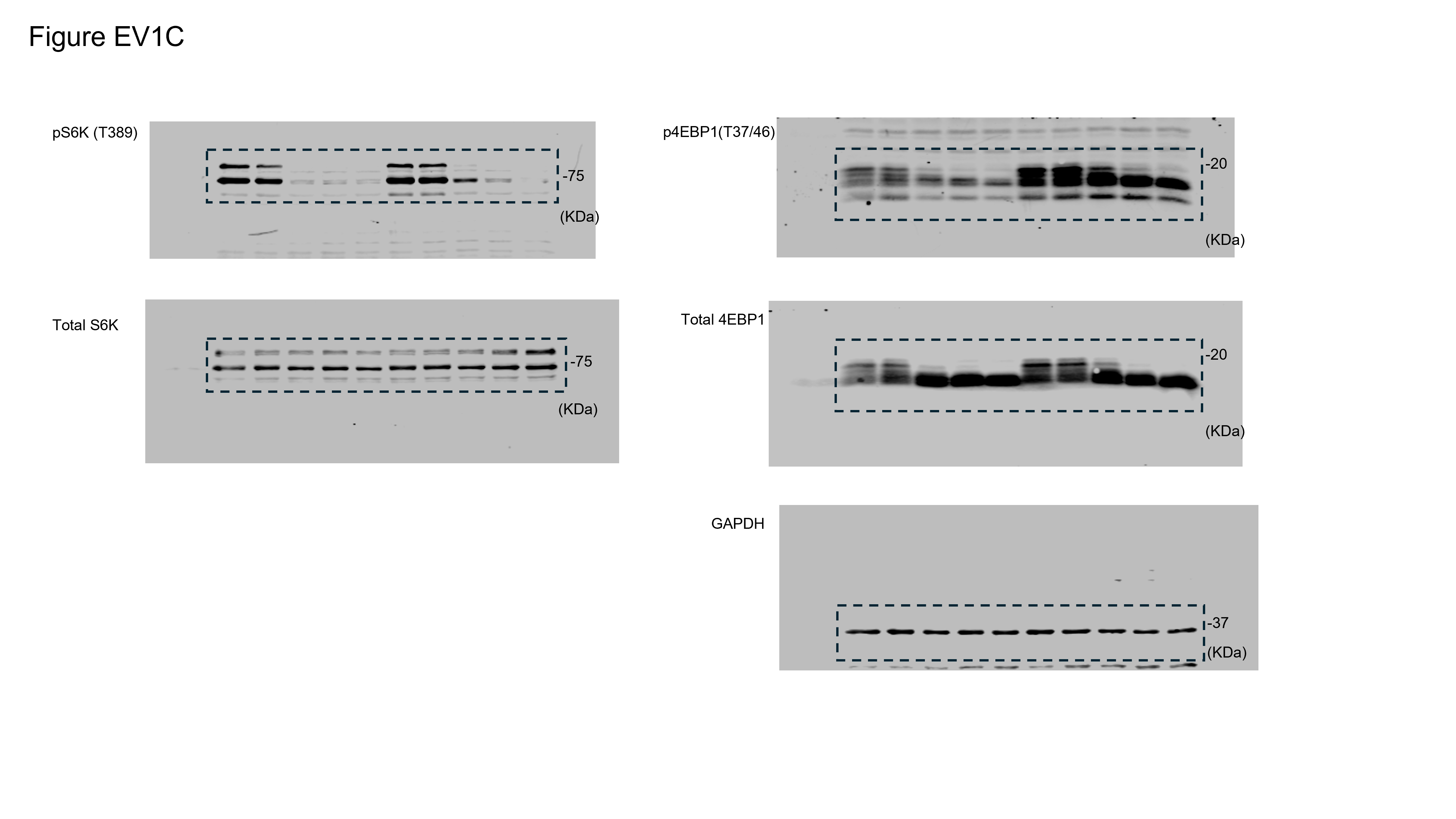

Supplement: Supplementary file 13 — Figure EV1 Source Data [file 44318_2026_791_MOESM13_ESM.zip › Figure EV1/EV1C-D/FigureEV1C_western.tif]

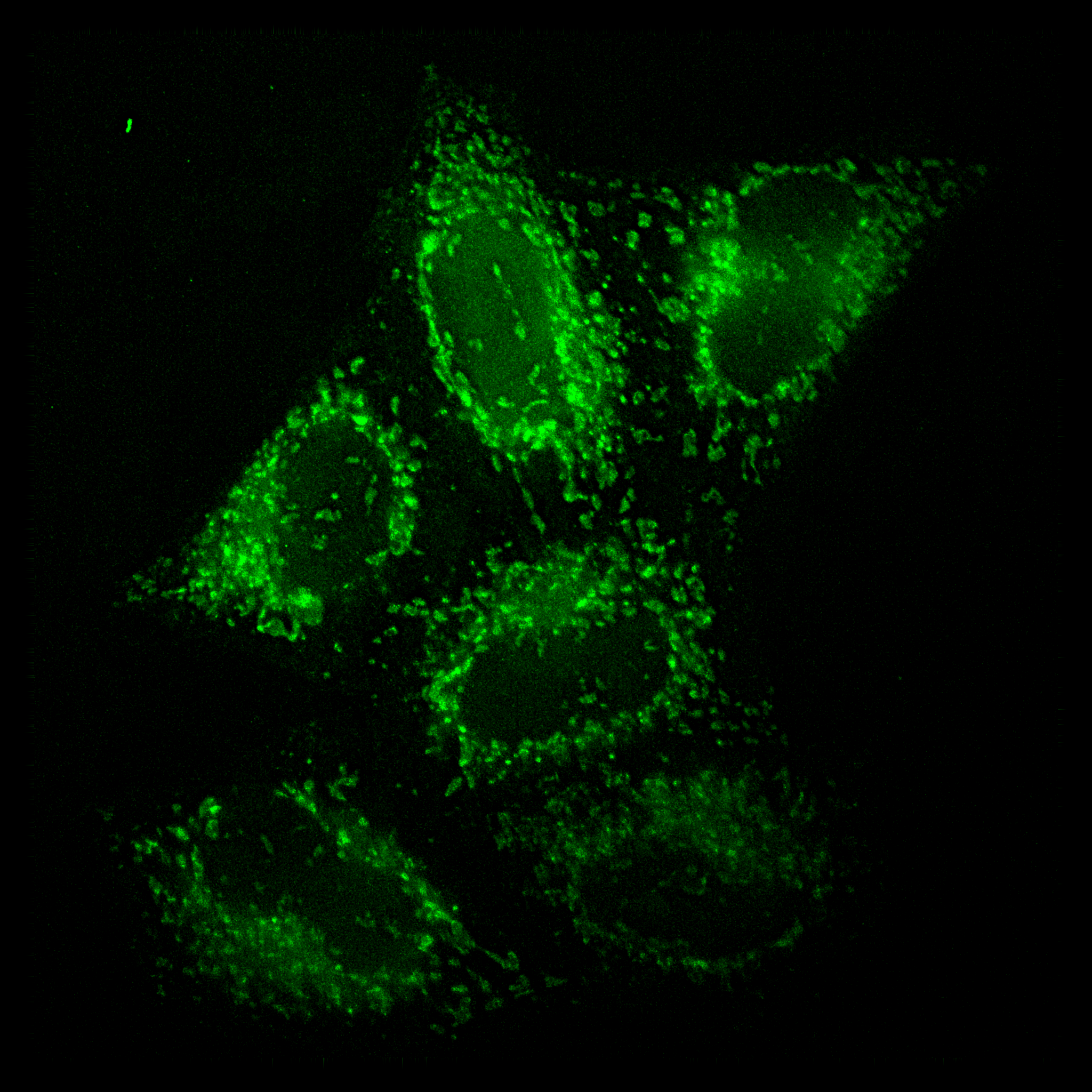

Supplement: Supplementary file 13 — Figure EV1 Source Data [file 44318_2026_791_MOESM13_ESM.zip › Figure EV1/EV1A/FigureEV1A_Hela_TFEB_GFP_sgLYSET_DMSO.tif]

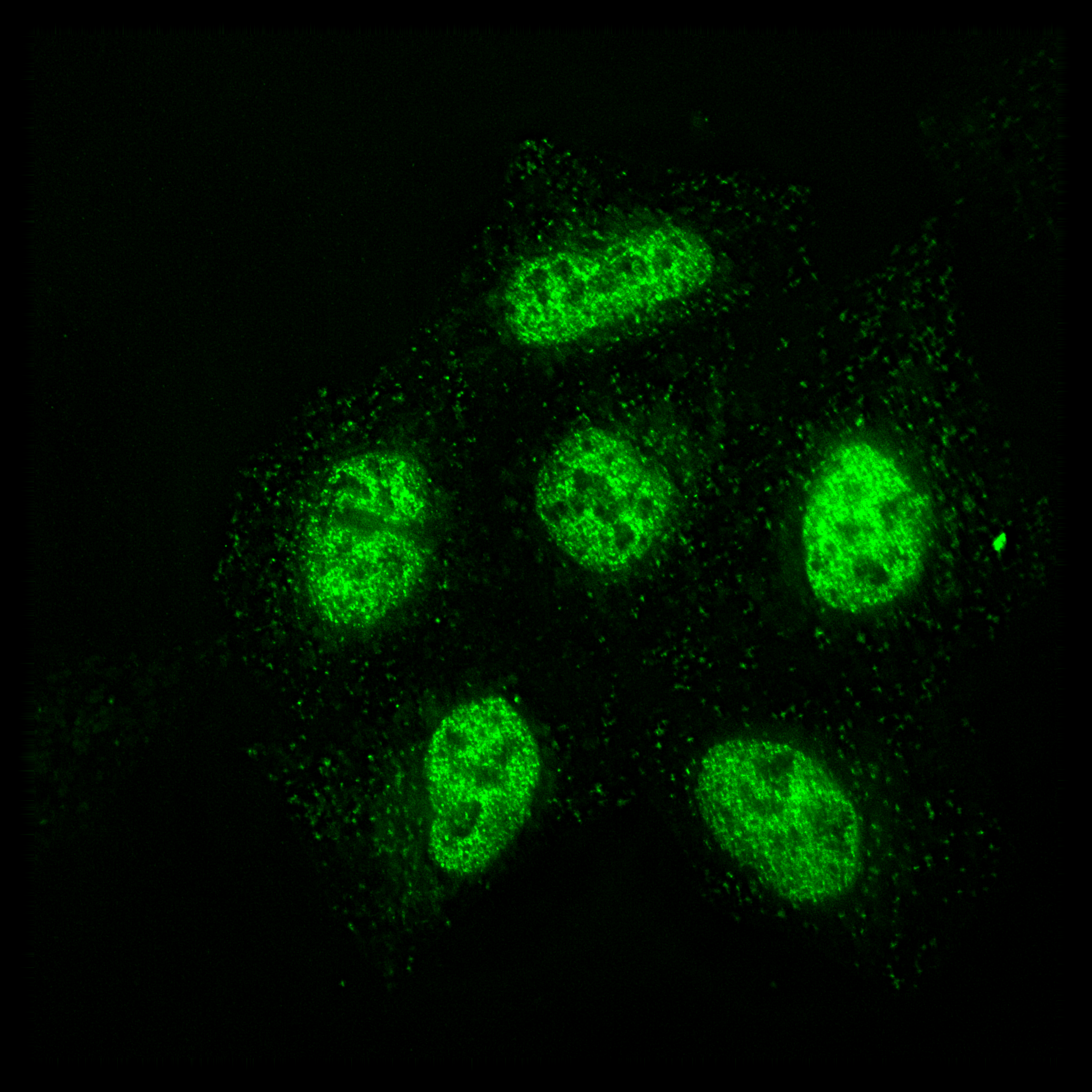

Supplement: Supplementary file 13 — Figure EV1 Source Data [file 44318_2026_791_MOESM13_ESM.zip › Figure EV1/EV1A/FigureEV1A_Hela_TFEB_GFP_Torin1.tif]

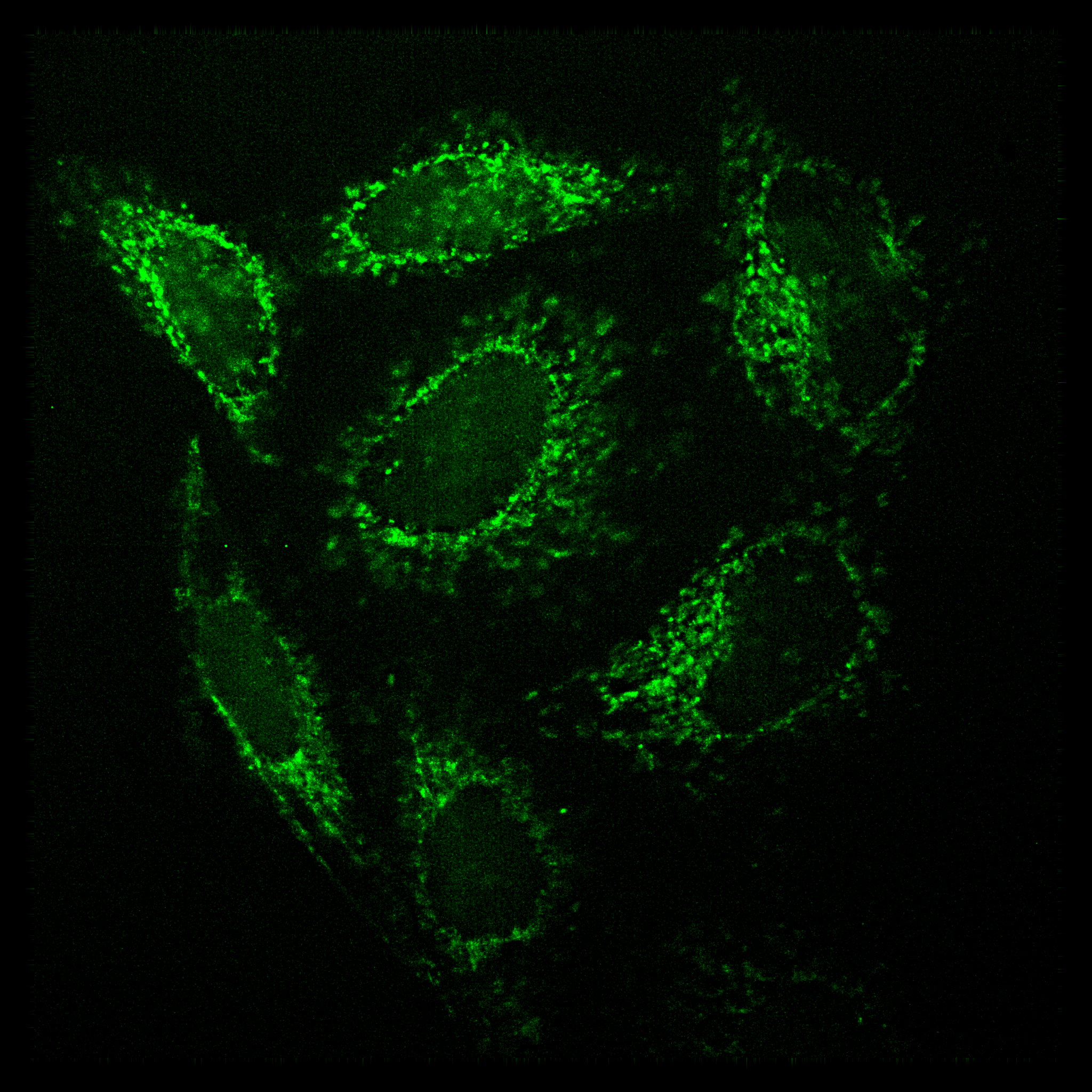

Supplement: Supplementary file 13 — Figure EV1 Source Data [file 44318_2026_791_MOESM13_ESM.zip › Figure EV1/EV1A/FigureEV1A_Hela_TFEB_GFP_DMSO.tif]

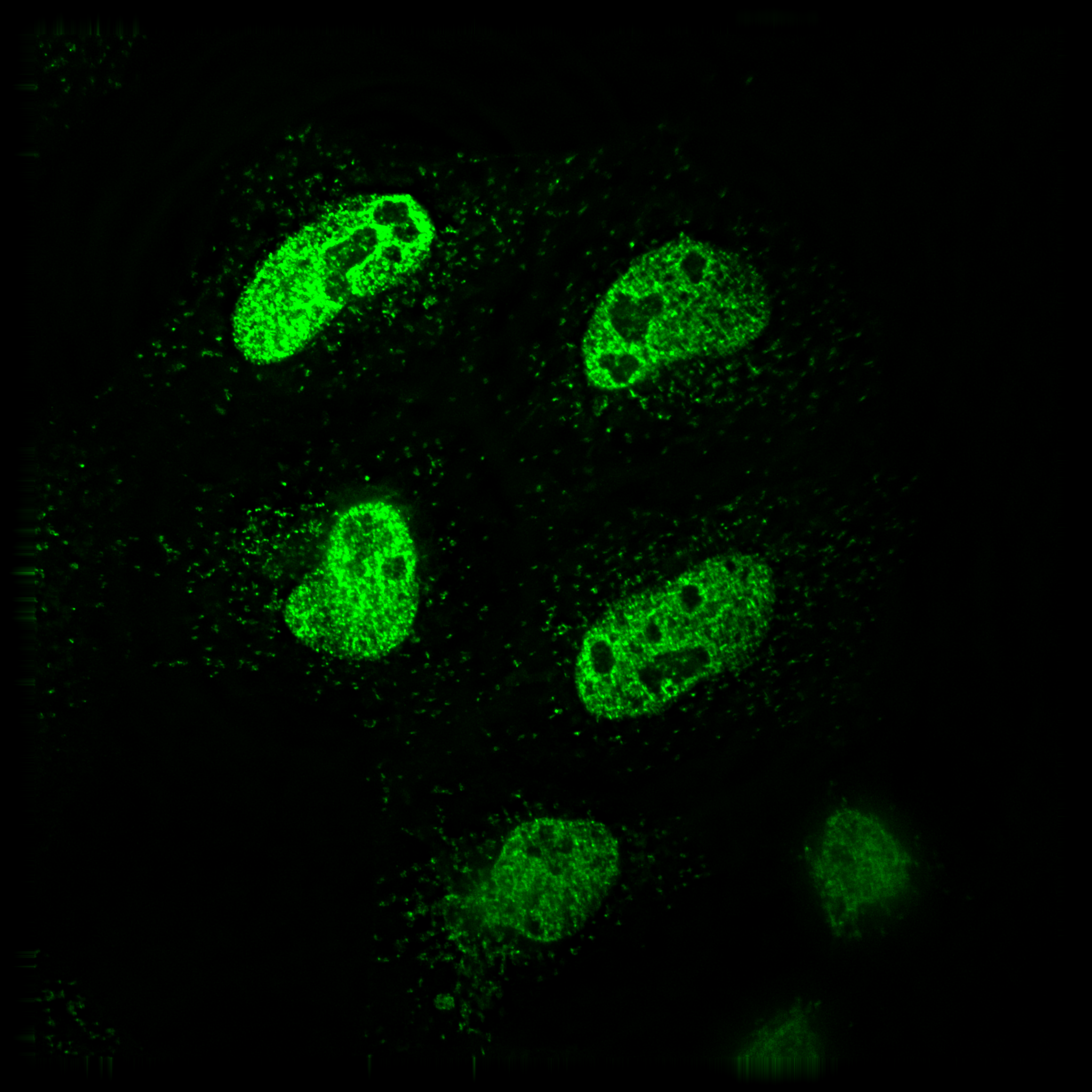

Supplement: Supplementary file 13 — Figure EV1 Source Data [file 44318_2026_791_MOESM13_ESM.zip › Figure EV1/EV1A/FigureEV1A_Hela_TFEB_GFP_sgLYSET_Torin1.tif]

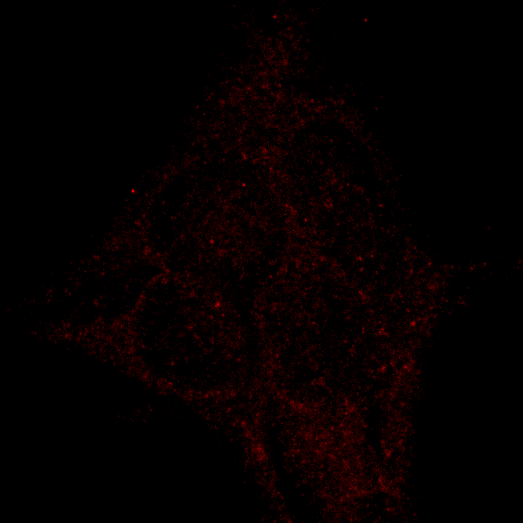

Supplement: Supplementary file 14 — Figure EV3 Source Data [file 44318_2026_791_MOESM14_ESM.zip › Figure EV3/EV3B/FigureEV3B_sgPEX19_FAR1.tif]

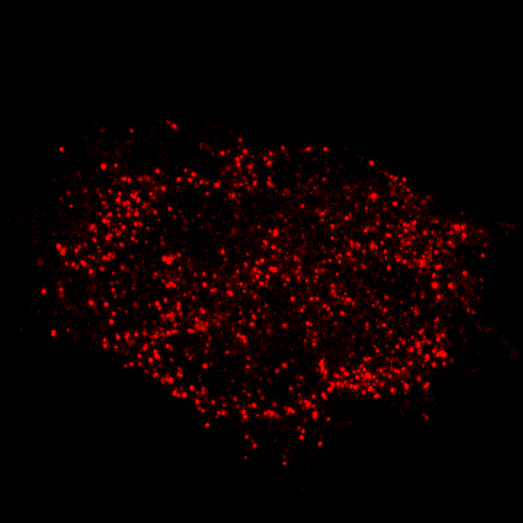

Supplement: Supplementary file 14 — Figure EV3 Source Data [file 44318_2026_791_MOESM14_ESM.zip › Figure EV3/EV3B/FigureEV3B_FAR1.tif]

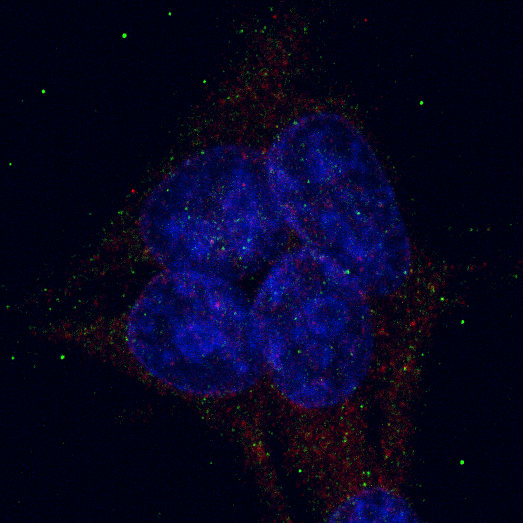

Supplement: Supplementary file 14 — Figure EV3 Source Data [file 44318_2026_791_MOESM14_ESM.zip › Figure EV3/EV3B/FigureEV3B_sgPEX19_Merge.tif]

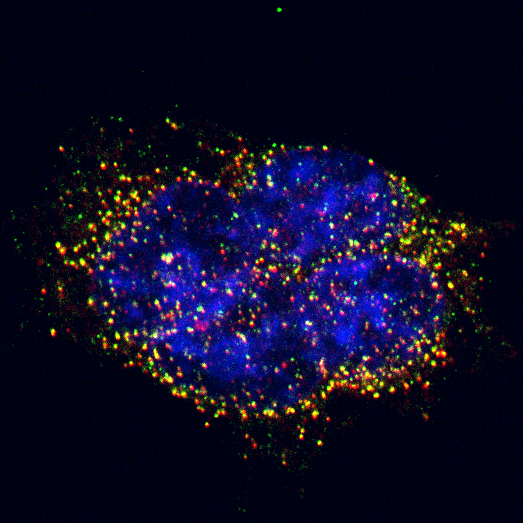

Supplement: Supplementary file 14 — Figure EV3 Source Data [file 44318_2026_791_MOESM14_ESM.zip › Figure EV3/EV3B/FigureEV3B_Merge.tif]

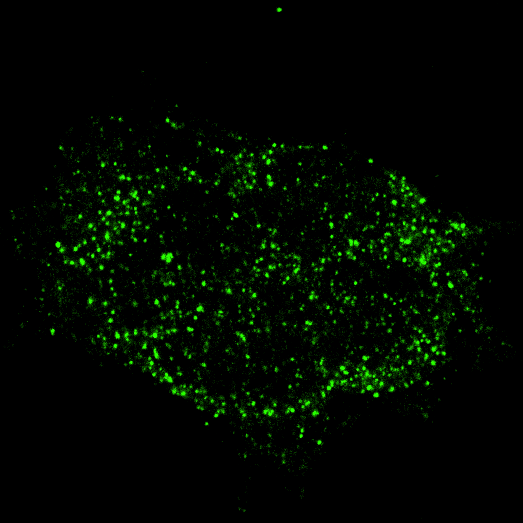

Supplement: Supplementary file 14 — Figure EV3 Source Data [file 44318_2026_791_MOESM14_ESM.zip › Figure EV3/EV3B/FigureEV3B_PMP70.tif]

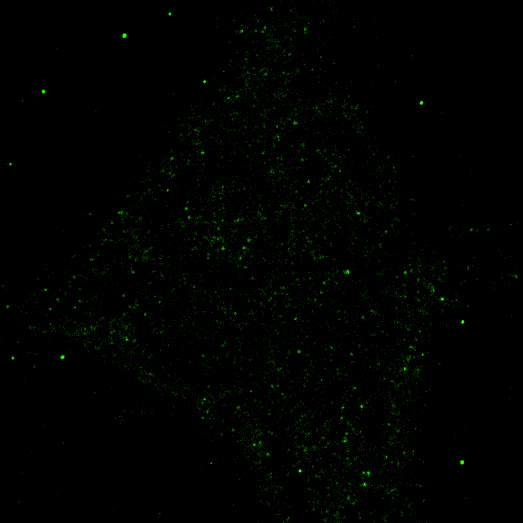

Supplement: Supplementary file 14 — Figure EV3 Source Data [file 44318_2026_791_MOESM14_ESM.zip › Figure EV3/EV3B/FigureEV3B_sgPEX19_PMP70.tif]

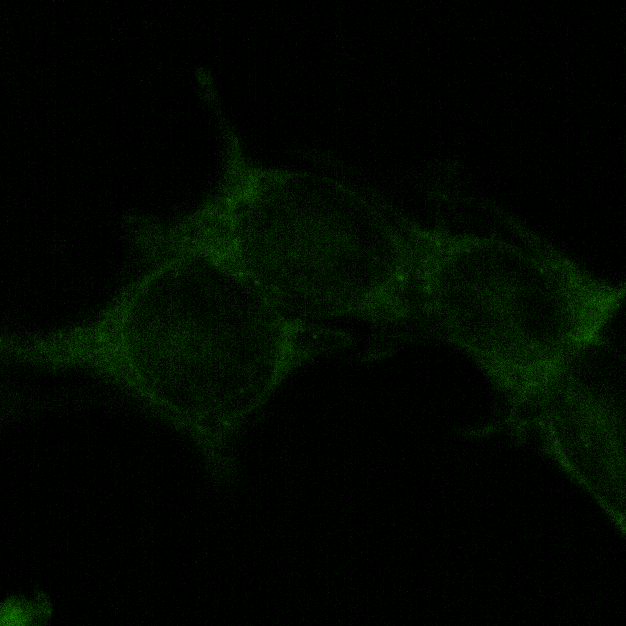

Supplement: Supplementary file 14 — Figure EV3 Source Data [file 44318_2026_791_MOESM14_ESM.zip › Figure EV3/EV3C/FigureEV3C_EGFPGNPAT_sgPEX5_EGFP.tif]

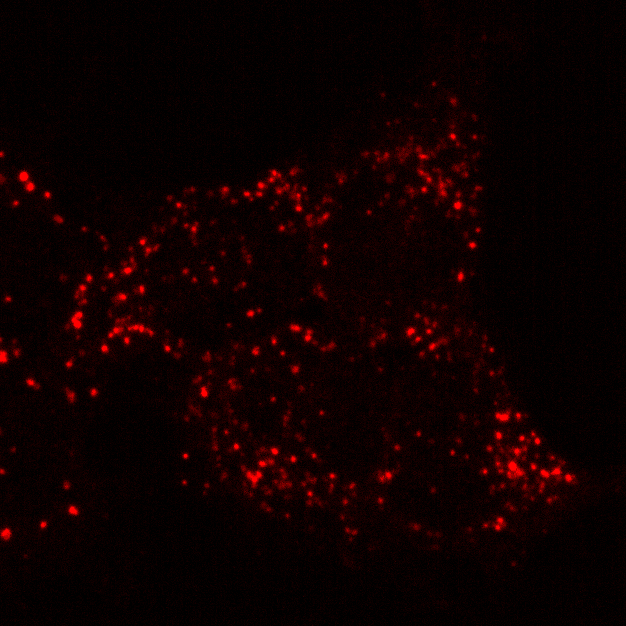

Supplement: Supplementary file 14 — Figure EV3 Source Data [file 44318_2026_791_MOESM14_ESM.zip › Figure EV3/EV3C/FigureEV3C_EGFPGNPAT_PMP70.tif]

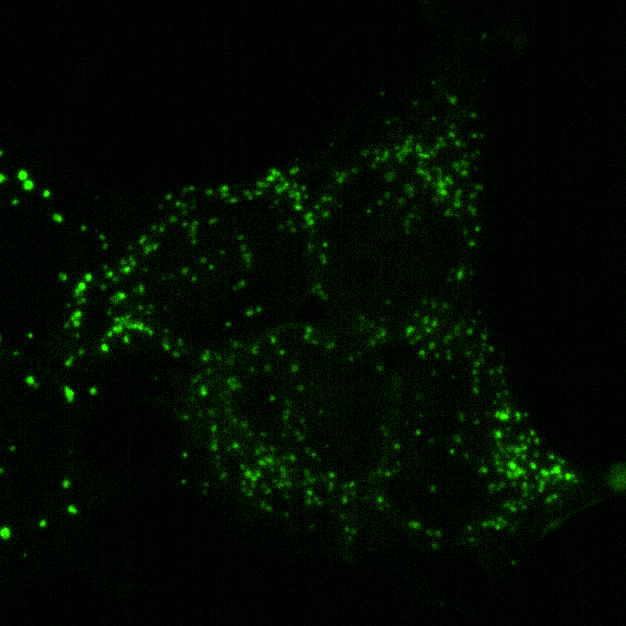

Supplement: Supplementary file 14 — Figure EV3 Source Data [file 44318_2026_791_MOESM14_ESM.zip › Figure EV3/EV3C/FigureEV3C_EGFPGNPAT_EGFP.tif]

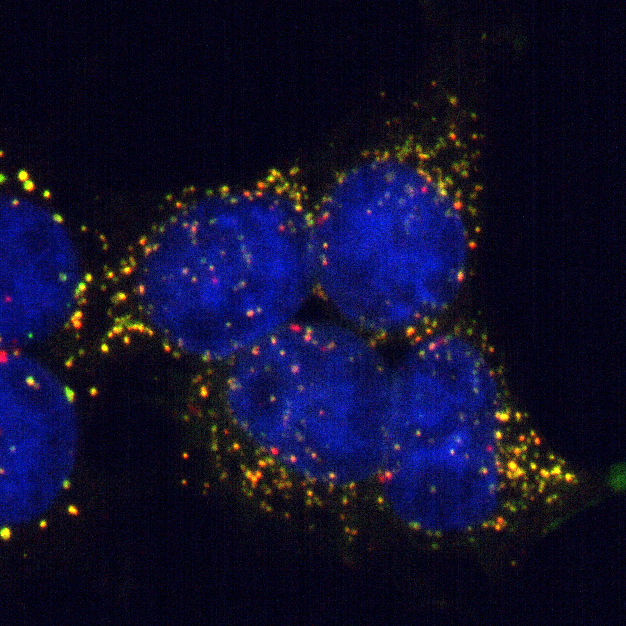

Supplement: Supplementary file 14 — Figure EV3 Source Data [file 44318_2026_791_MOESM14_ESM.zip › Figure EV3/EV3C/FigureEV3C_EGFPGNPAT_Merge.tif]

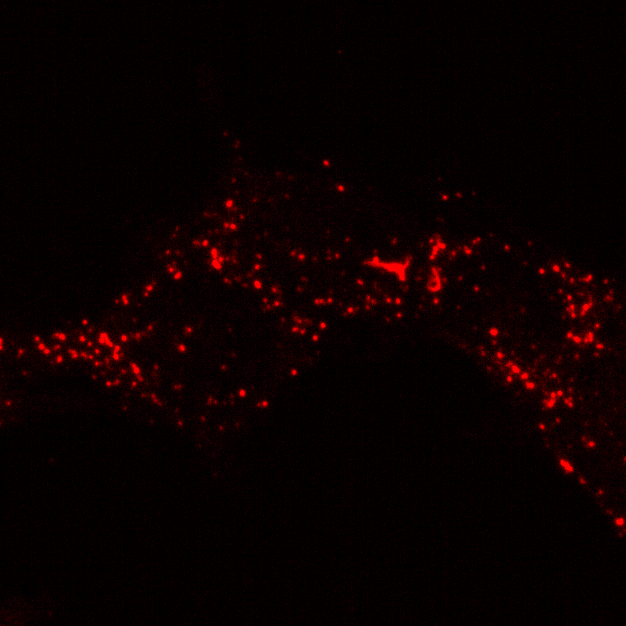

Supplement: Supplementary file 14 — Figure EV3 Source Data [file 44318_2026_791_MOESM14_ESM.zip › Figure EV3/EV3C/FigureEV3C_EGFPGNPAT_sgPEX5_PMP70.tif]

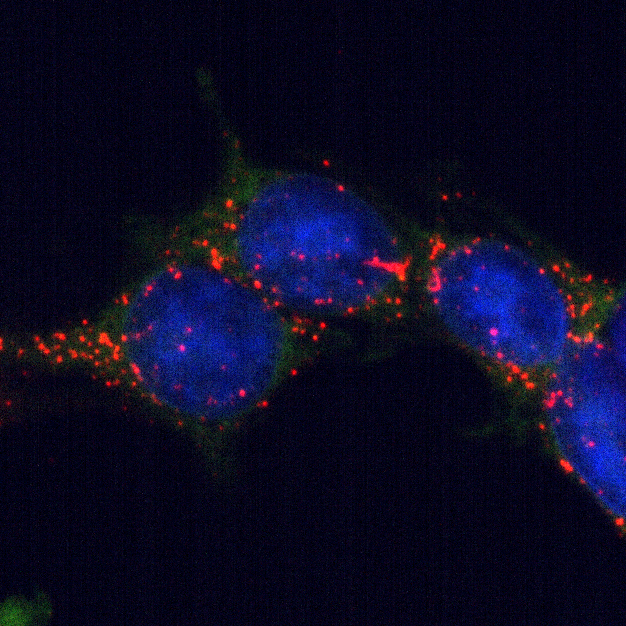

Supplement: Supplementary file 14 — Figure EV3 Source Data [file 44318_2026_791_MOESM14_ESM.zip › Figure EV3/EV3C/FigureEV3C_EGFPGNPAT_sgPEX5_Merge.tif]

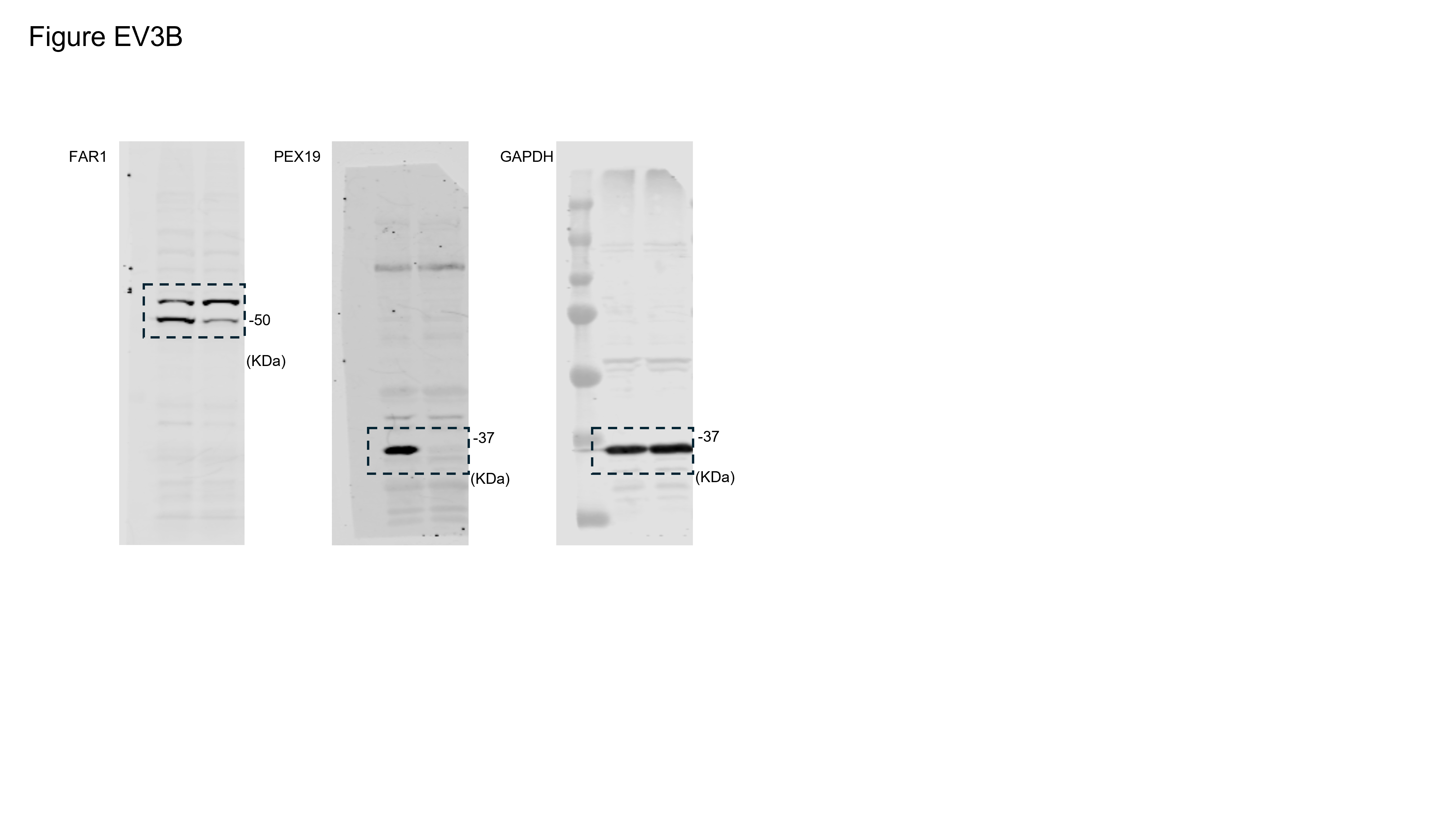

Supplement: Supplementary file 14 — Figure EV3 Source Data [file 44318_2026_791_MOESM14_ESM.zip › Figure EV3/EV3G/FigureEV3G_western.tif]

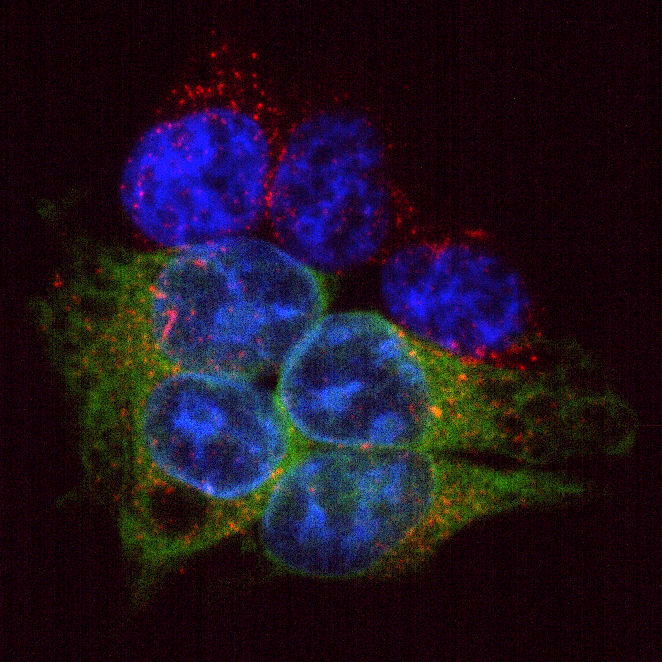

Supplement: Supplementary file 14 — Figure EV3 Source Data [file 44318_2026_791_MOESM14_ESM.zip › Figure EV3/EV3D/FigureEV3D_AGPSEGFP_sgPEX7_merge.tif]

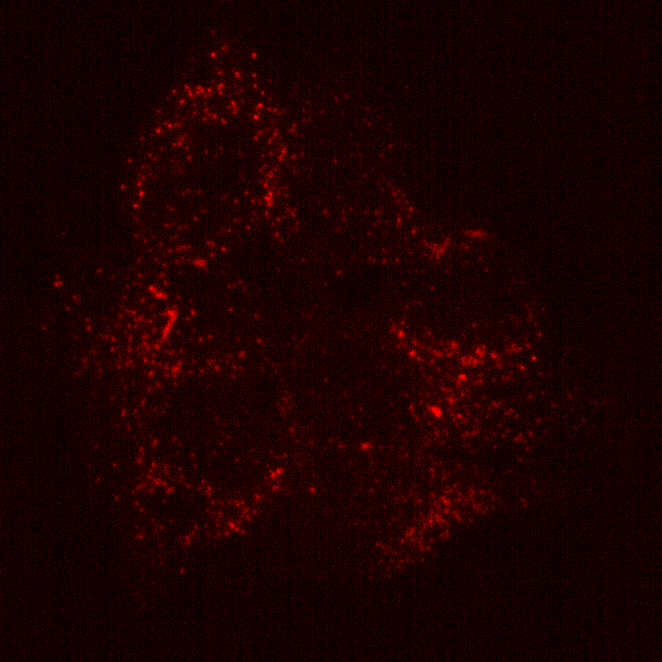

Supplement: Supplementary file 14 — Figure EV3 Source Data [file 44318_2026_791_MOESM14_ESM.zip › Figure EV3/EV3D/FigureEV3D_AGPSEGFP_sgPEX7_PMP70.tif]

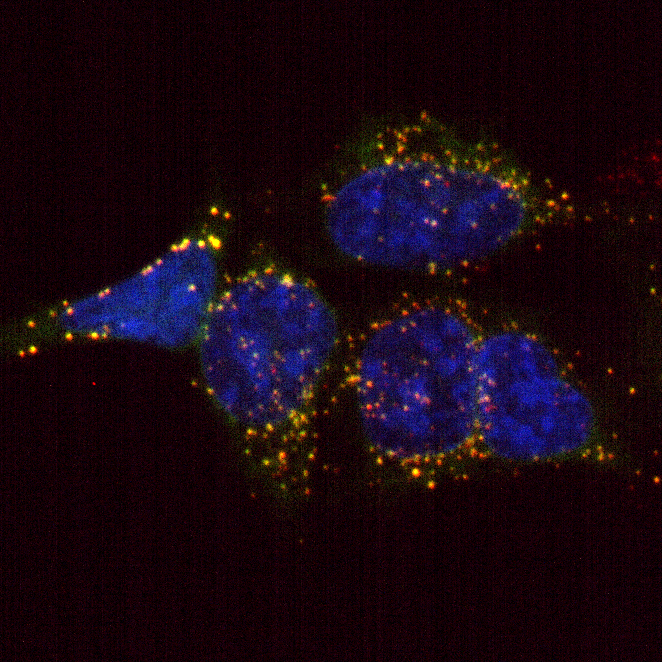

Supplement: Supplementary file 14 — Figure EV3 Source Data [file 44318_2026_791_MOESM14_ESM.zip › Figure EV3/EV3D/FigureEV3D_AGPSEGFP_merge.tif]

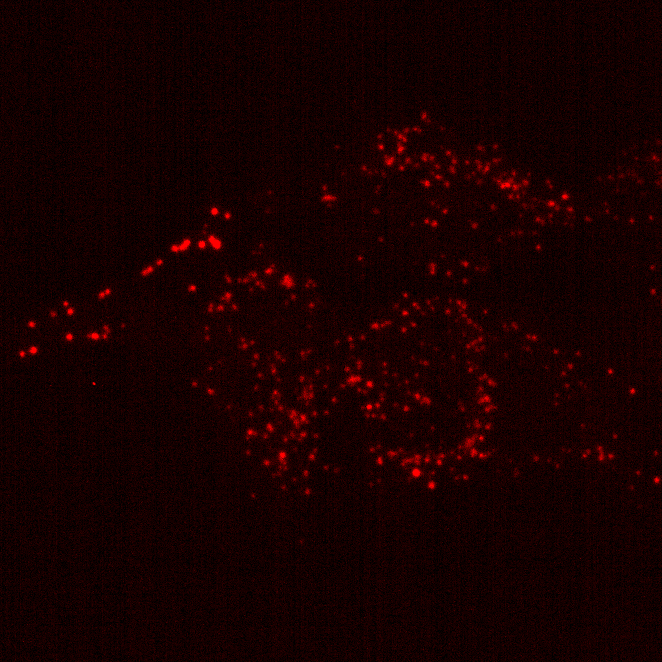

Supplement: Supplementary file 14 — Figure EV3 Source Data [file 44318_2026_791_MOESM14_ESM.zip › Figure EV3/EV3D/FigureEV3D_AGPSEGFP_PMP70.tif]

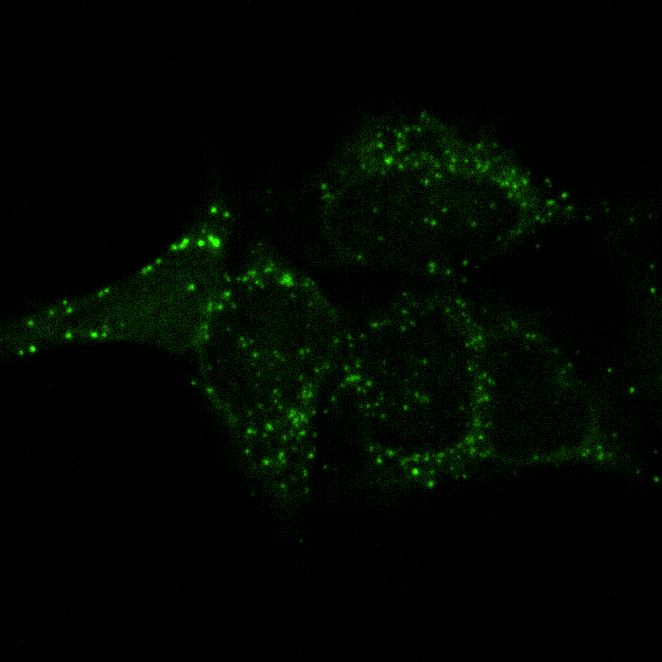

Supplement: Supplementary file 14 — Figure EV3 Source Data [file 44318_2026_791_MOESM14_ESM.zip › Figure EV3/EV3D/FigureEV3D_AGPSEGFP_EGFP.tif]

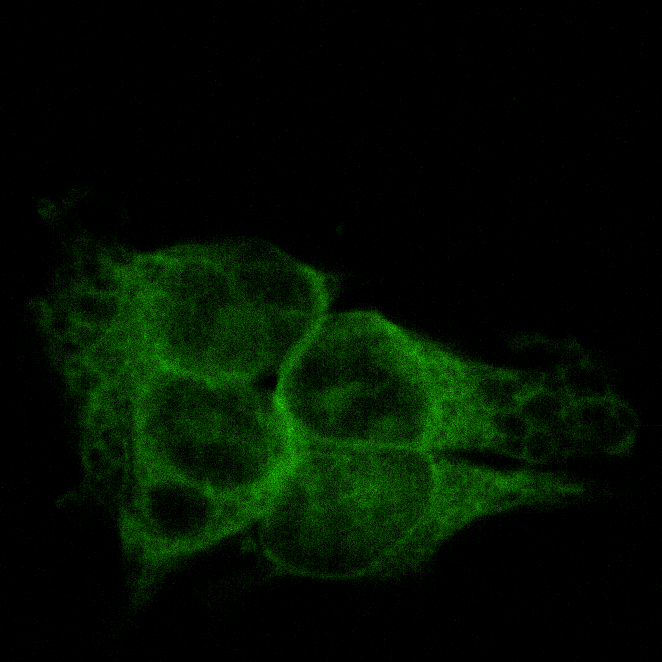

Supplement: Supplementary file 14 — Figure EV3 Source Data [file 44318_2026_791_MOESM14_ESM.zip › Figure EV3/EV3D/FigureEV3D_AGPSEGFP_sgPEX7_EGFP.tif]

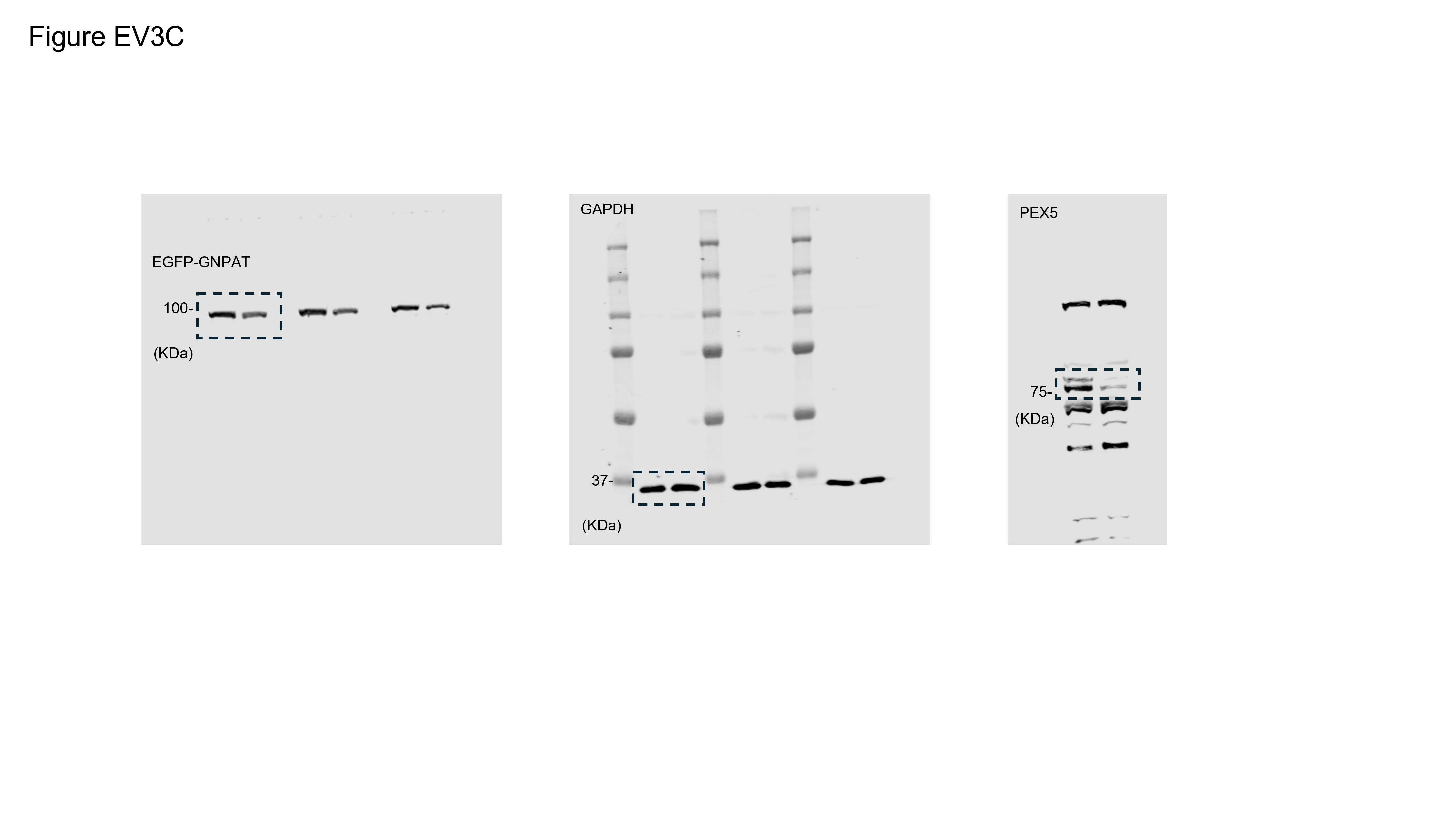

Supplement: Supplementary file 14 — Figure EV3 Source Data [file 44318_2026_791_MOESM14_ESM.zip › Figure EV3/EV3H/FigureEV3H_western.tif]

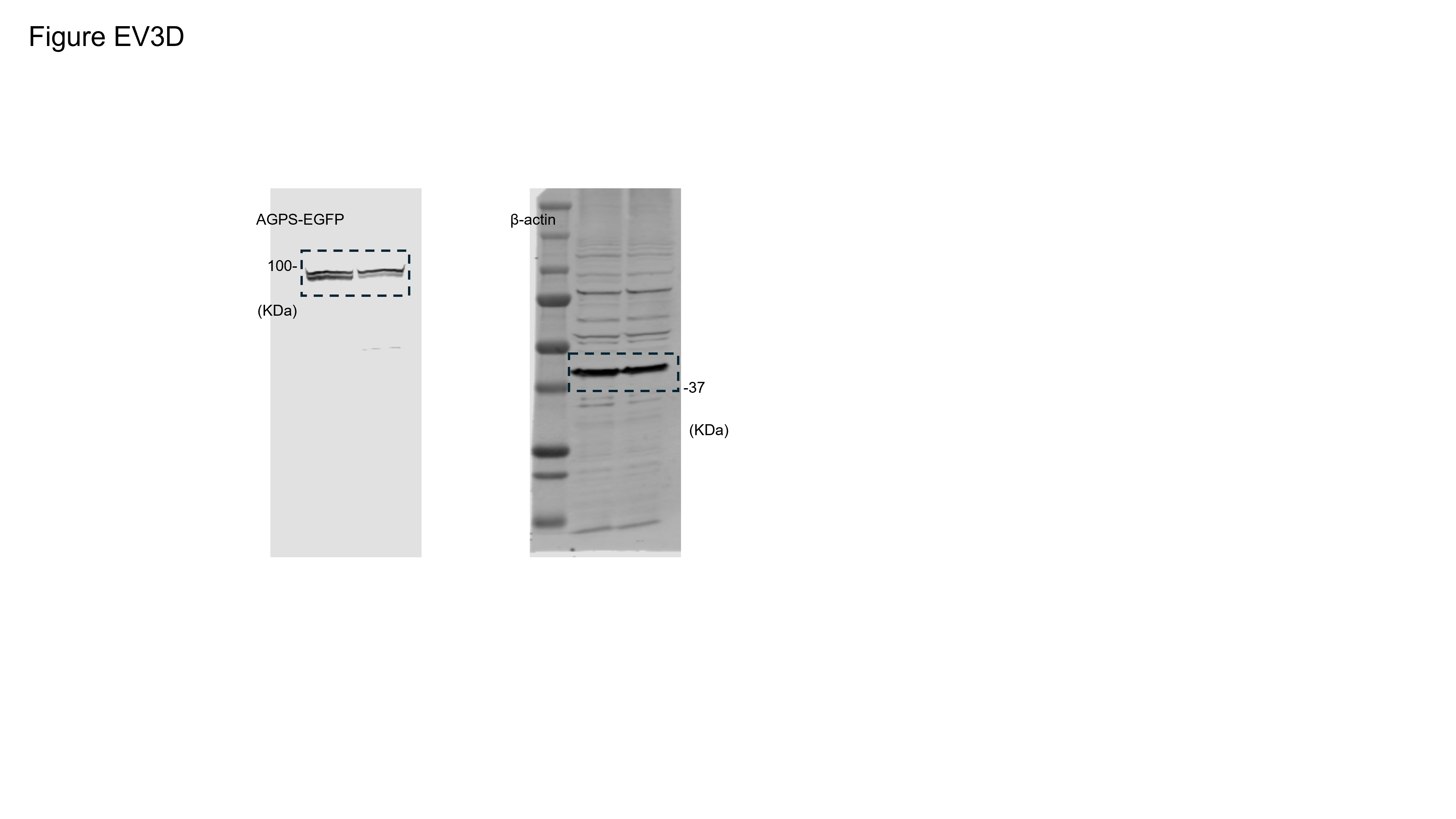

Supplement: Supplementary file 14 — Figure EV3 Source Data [file 44318_2026_791_MOESM14_ESM.zip › Figure EV3/EV3I/FigureEV3I_western.tif]

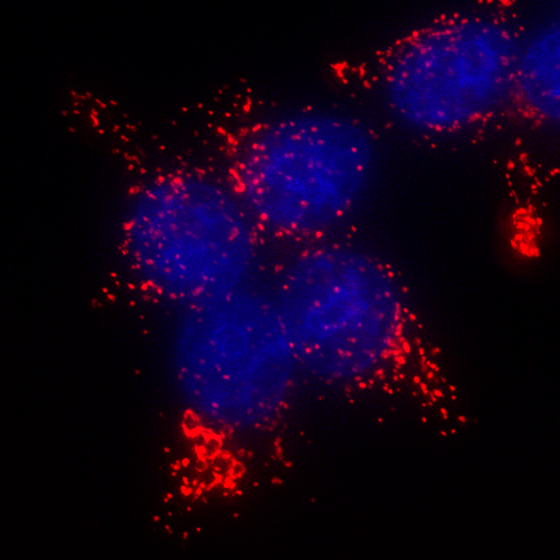

Supplement: Supplementary file 15 — Figure EV4 Source Data [file 44318_2026_791_MOESM15_ESM.zip › Figure EV4/EV4B-C/FigureEV4B_FAR1KO.tif]

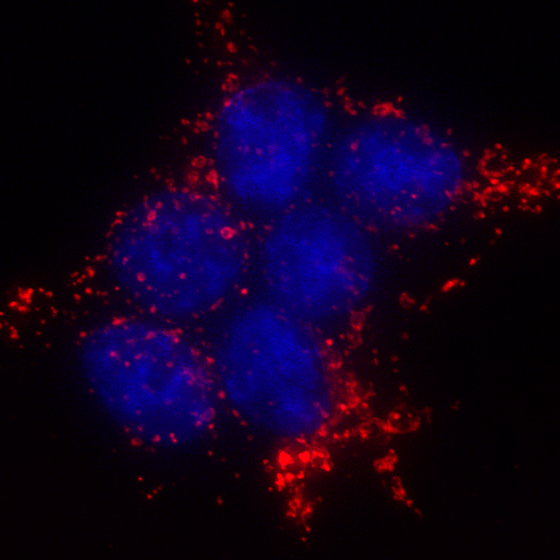

Supplement: Supplementary file 15 — Figure EV4 Source Data [file 44318_2026_791_MOESM15_ESM.zip › Figure EV4/EV4B-C/FigureEV4B_WT.tif]

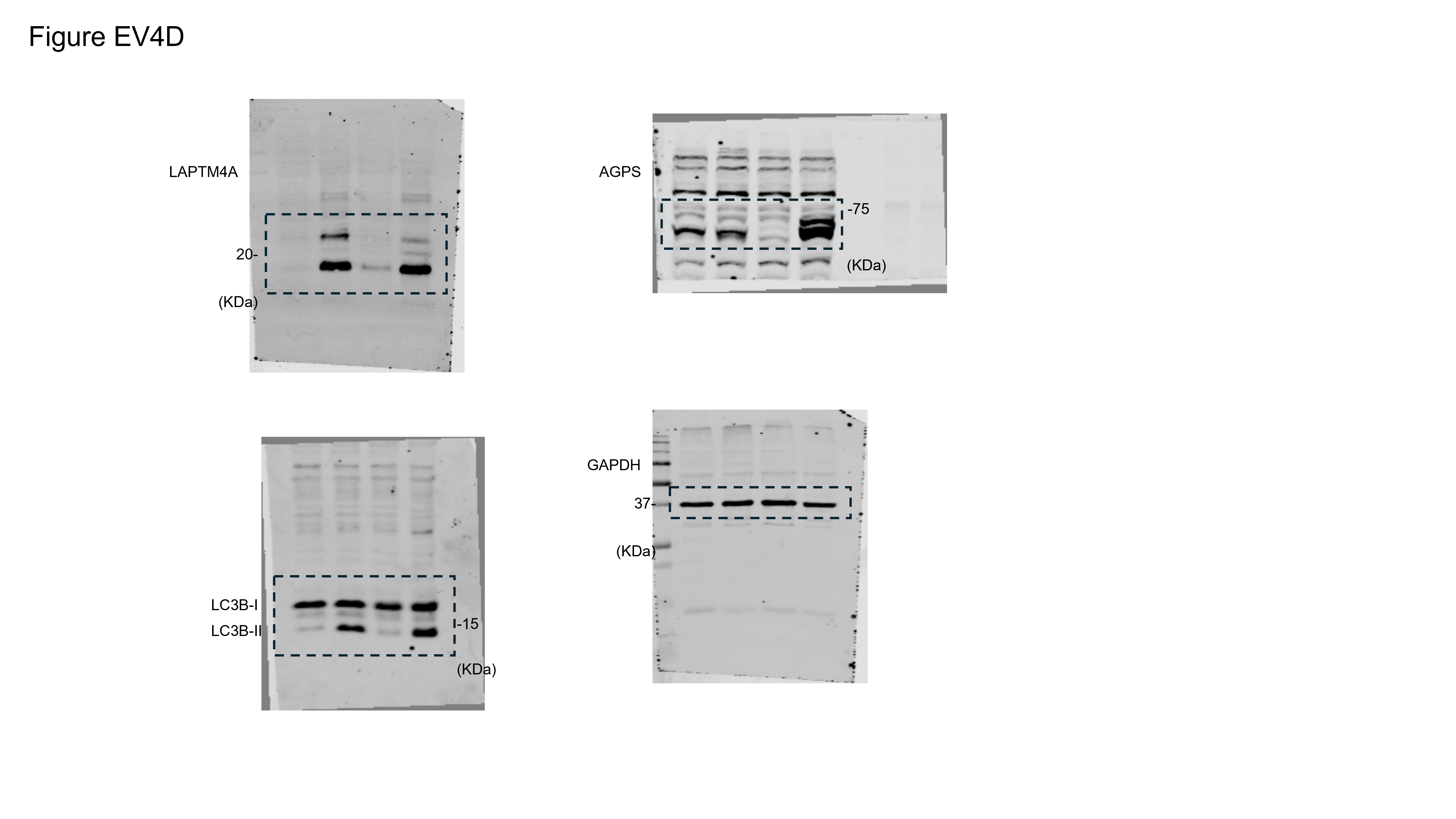

Supplement: Supplementary file 15 — Figure EV4 Source Data [file 44318_2026_791_MOESM15_ESM.zip › Figure EV4/EV4D-F/FigureEV4D_western.tif]

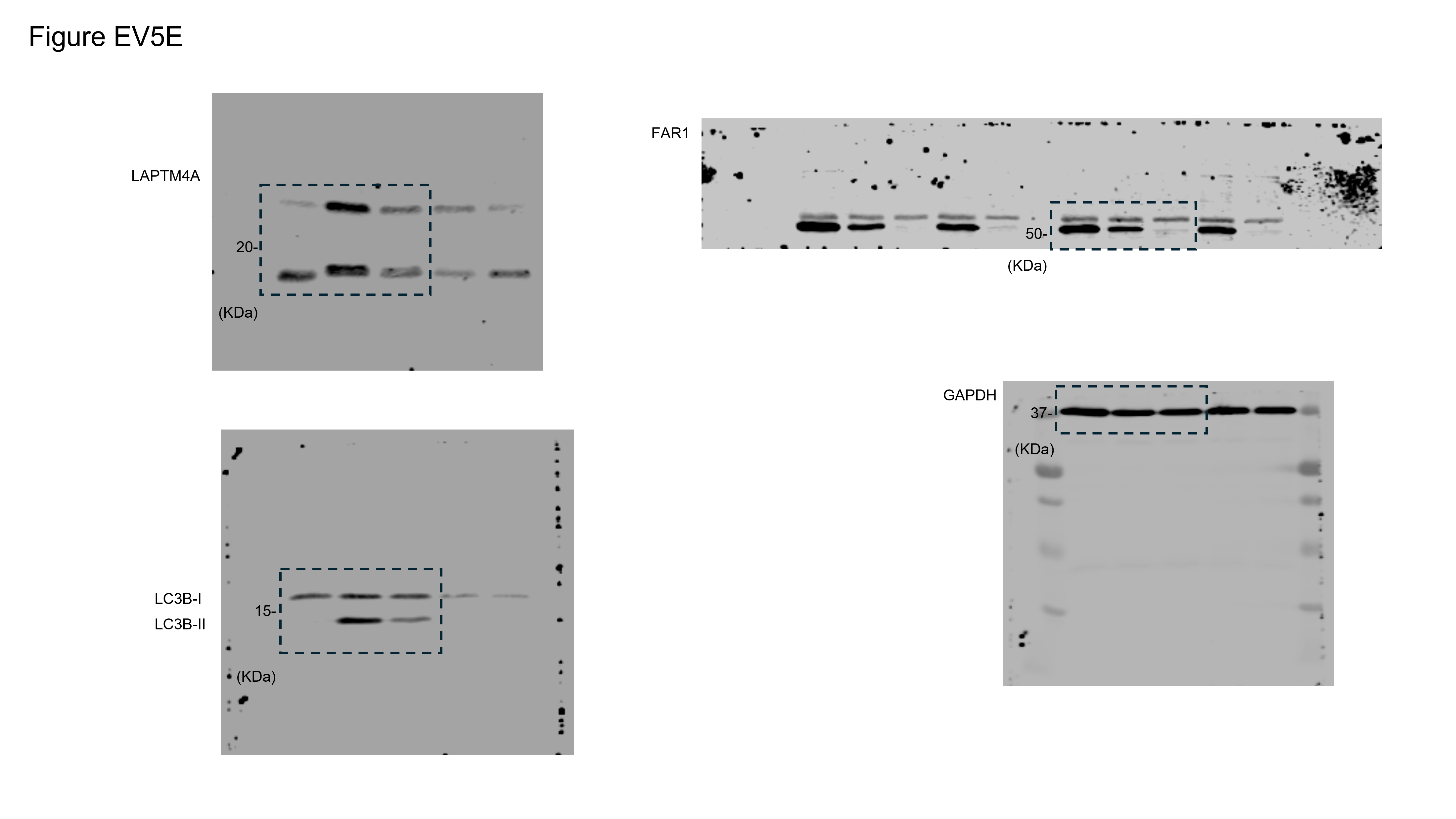

Supplement: Supplementary file 16 — Figure EV5 Source Data [file 44318_2026_791_MOESM16_ESM.zip › Figure EV5/EV5E/FigureEV5E_western.tif]

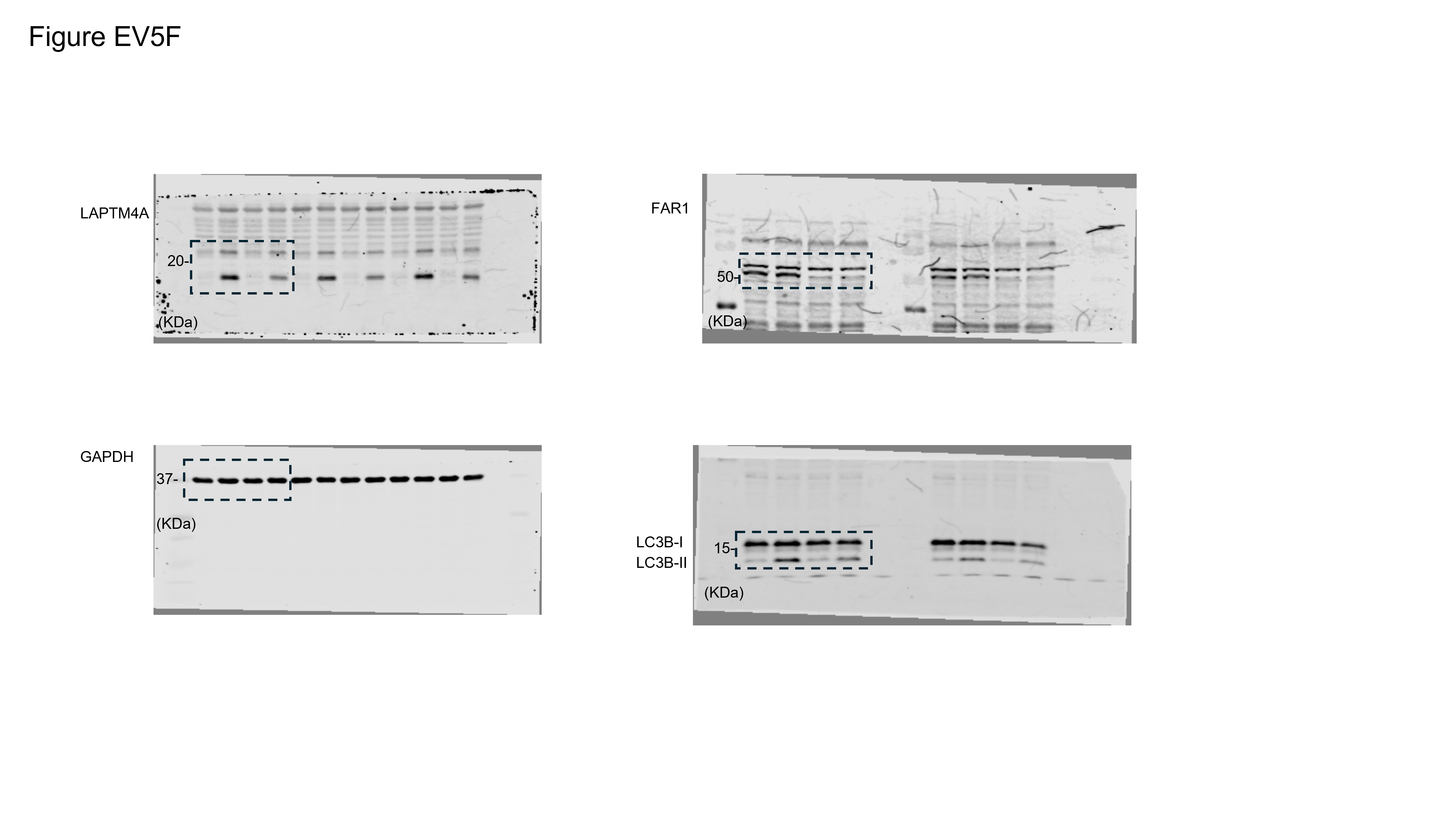

Supplement: Supplementary file 16 — Figure EV5 Source Data [file 44318_2026_791_MOESM16_ESM.zip › Figure EV5/EV5F-H/FigureEV5F_western.tif]

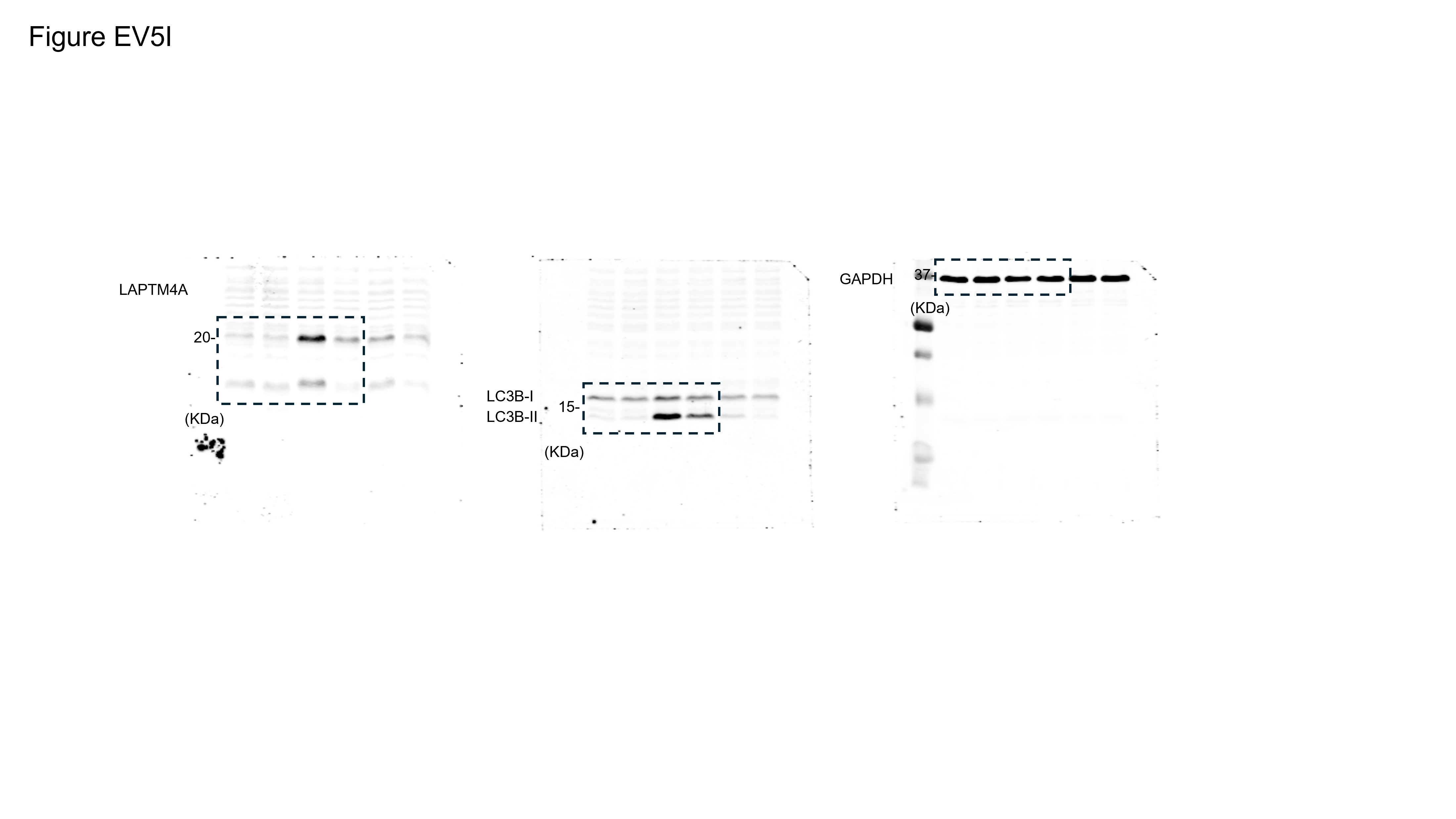

Supplement: Supplementary file 16 — Figure EV5 Source Data [file 44318_2026_791_MOESM16_ESM.zip › Figure EV5/EV5I-K/FigureEV5I_western.tif]

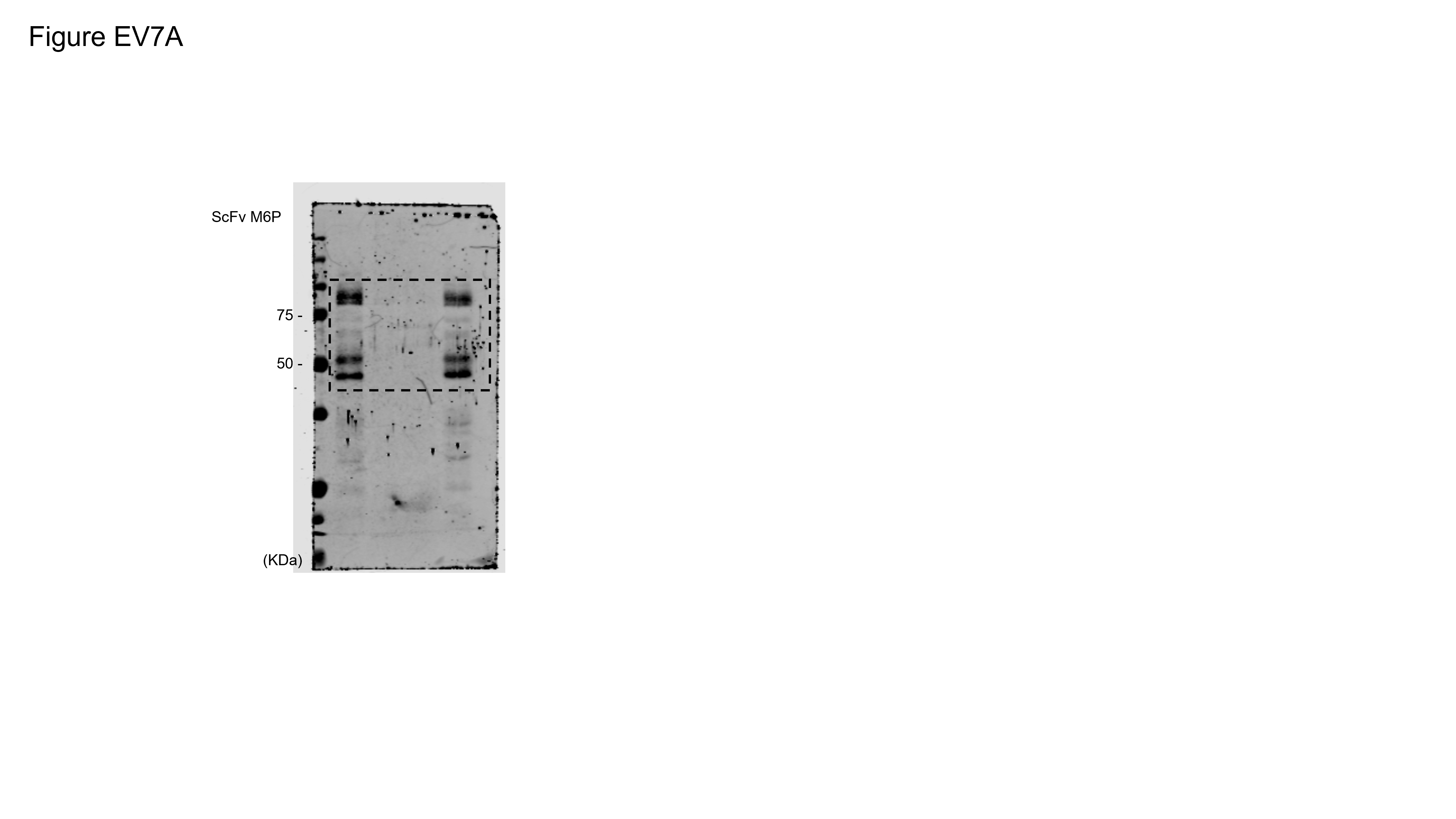

Supplement: Supplementary file 18 — Figure EV7 Source Data [file 44318_2026_791_MOESM18_ESM.zip › Figure EV7/EV7A/FigureEV7A_western.tif]

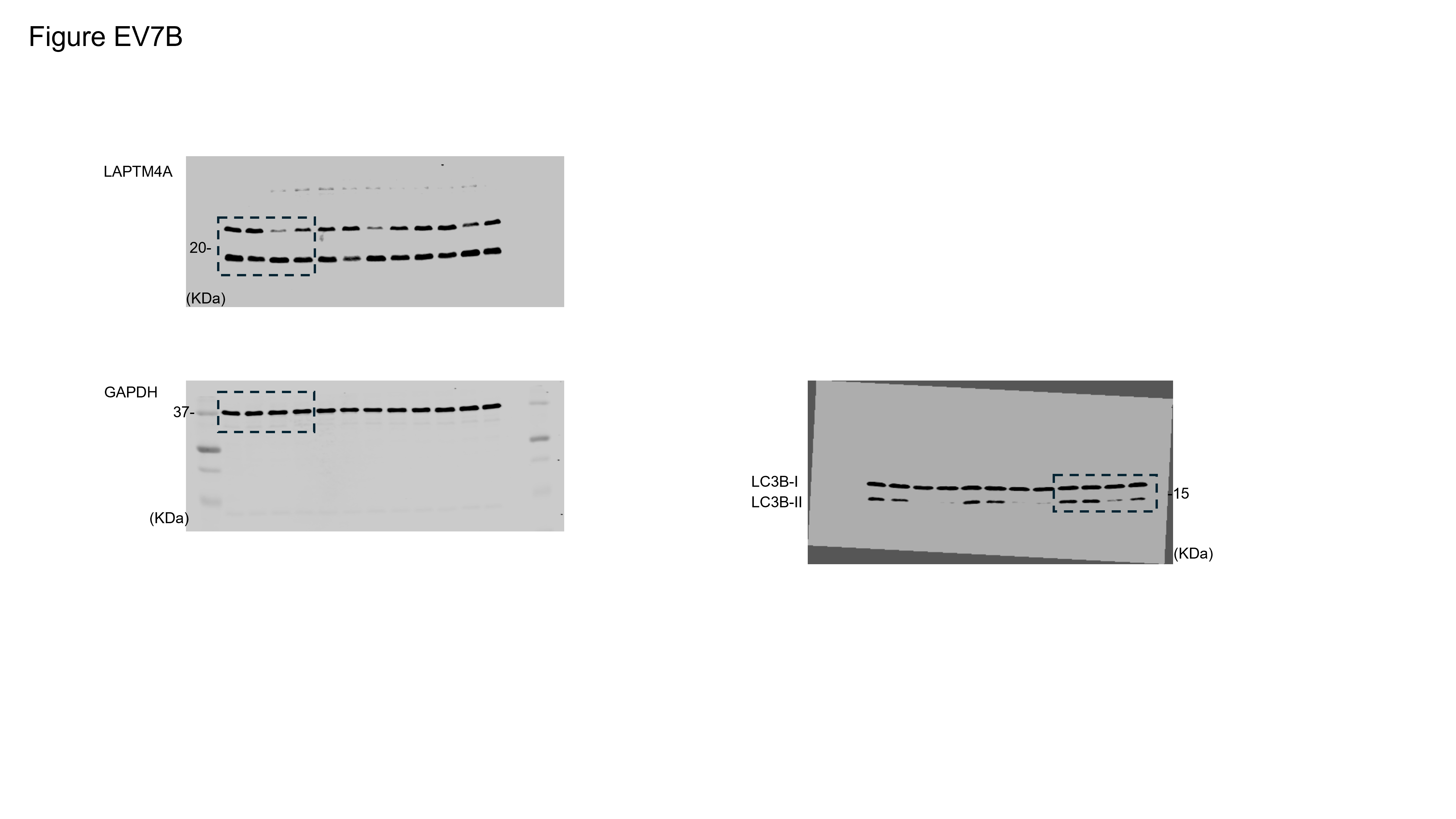

Supplement: Supplementary file 18 — Figure EV7 Source Data [file 44318_2026_791_MOESM18_ESM.zip › Figure EV7/EV7B-D/FigureEV7B_western.tif]

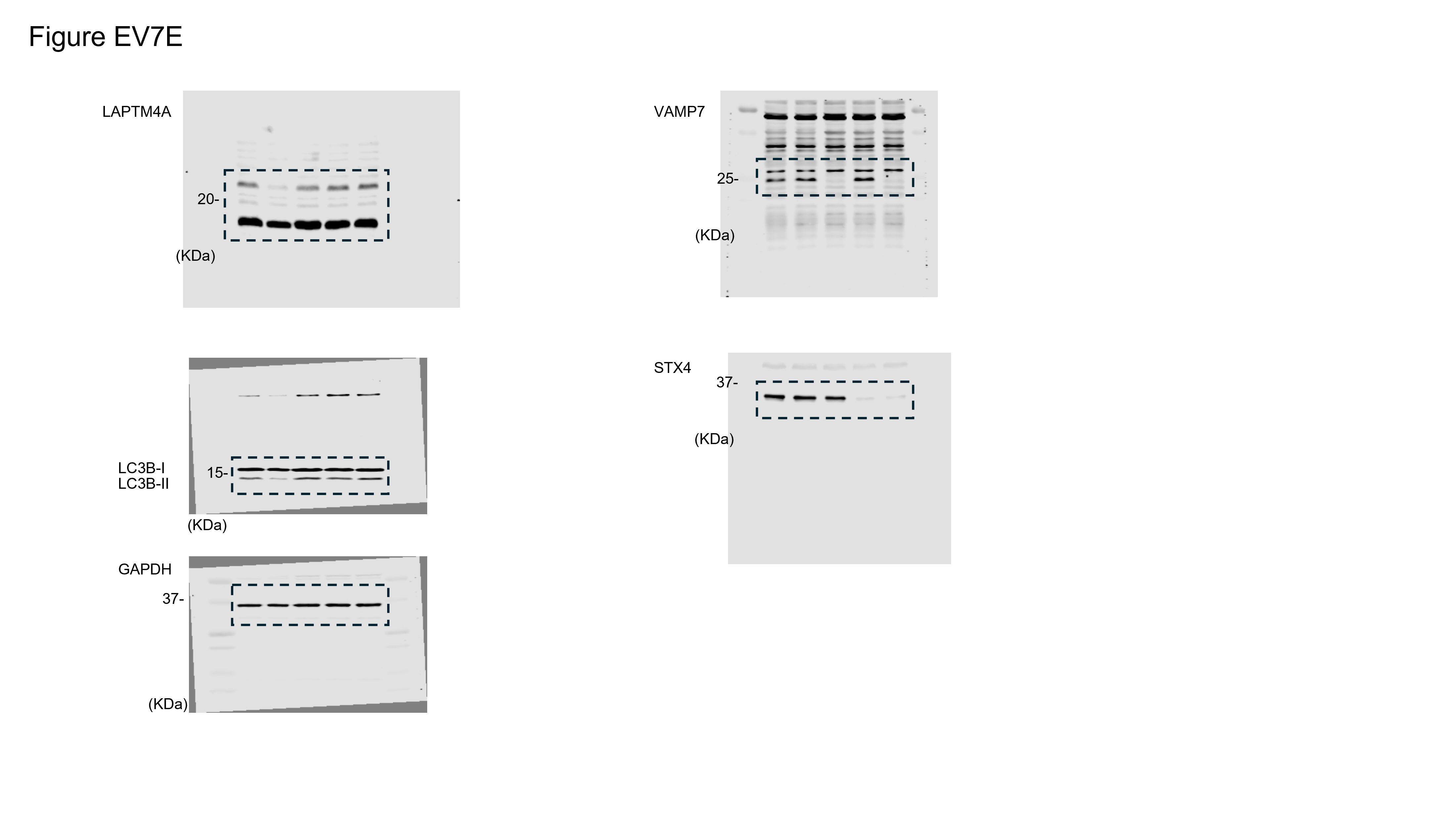

Supplement: Supplementary file 18 — Figure EV7 Source Data [file 44318_2026_791_MOESM18_ESM.zip › Figure EV7/EV7E-G/FigureEV7E_western.tif]
