# Supplementary material for: Peroxisome-derived ether lipids regulate lysosomal exocytosis
Source: EMBO J. 2026 May 2;45(11):3699–730. doi: 10.1038/s44318-026-00791-3 (PMC13226661; doi:10.1038/s44318-026-00791-3)
Supplement: Supplementary file 19 — Expanded View Figures [file 44318_2026_791_MOESM19_ESM.pdf]

Expanded View Figures

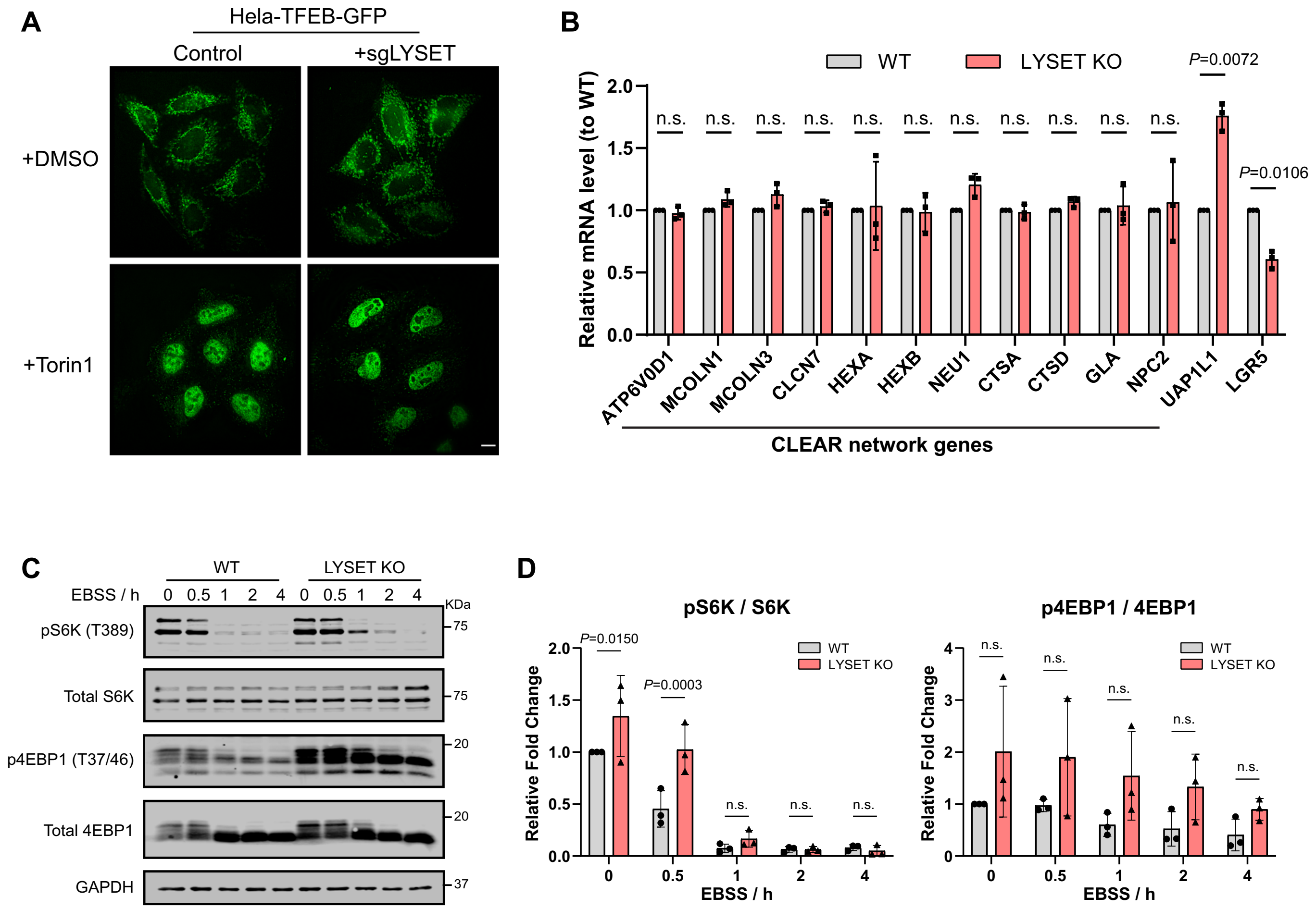

Figure EV1. Additional evidence for mTORC1- and TFEB/TFE3-independent lysosome accumulation in *LYSET* KO cells. Related to Figure 1.

(**A**) Fluorescence images of WT and *LYSET* KO HeLa cells stably expressing TFEB-GFP. Cells were treated with either dimethyl sulfoxide (DMSO) or 200 nM Torin1 (an mTORC1 inhibitor) for 1 hr. Scale bar: 10 µm. (**B**) Comparison of CLEAR network gene transcription levels in *LYSET* KO and WT HEK293T cells. Data were presented as mean ± s.d.; $n = 3$ biological replicates. *P* values were calculated using multiple unpaired t-tests with Welch's correction. (**C**) Western blot analysis of S6K and 4EBP1 phosphorylation in WT and *LYSET* KO HEK293T cells under EBSS treatment. The experiments were repeated three times with similar results. (**D**) Quantification of (**C**). Data were presented as mean ± s.d.; $n = 3$ biological replicates; *P* values were calculated by two-way ANOVA with multiple comparisons. Source data are available online for this figure.

 © The Author(s)

A

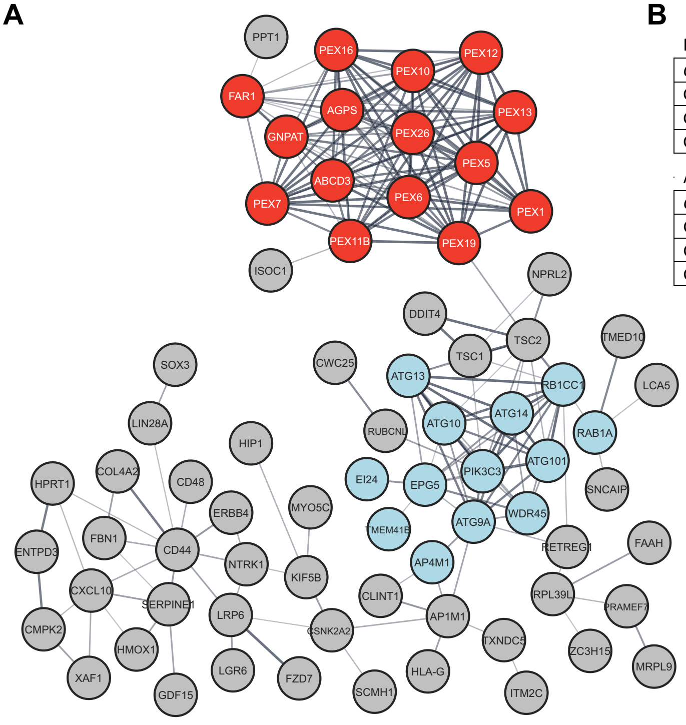

B

Peroxisome:

GO-term	Description	False Discovery Rate
GO:0005778	Peroxisomal membrane	8.85×10^{-13}
GO:0015919	Peroxisomal membrane transport	3.30×10^{-11}
GO:0007031	Peroxisome organization	3.36×10^{-11}

Autophagy:

GO-term	Description	False Discovery Rate
GO:0000045	Autophagosome assembly	4.04×10^{-6}
GO:0016236	Macroautophagy	7.83×10^{-8}
GO:0000407	Phagophore assembly site	3.45×10^{-5}

D

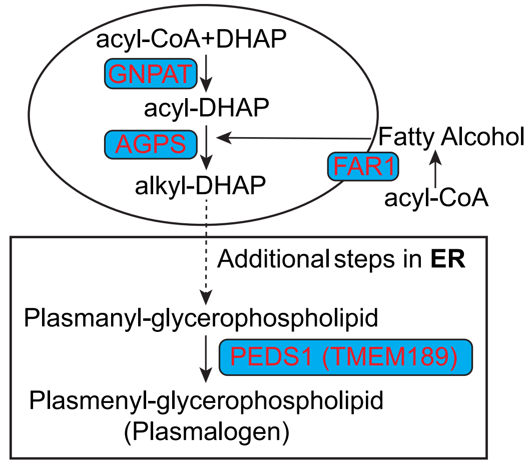

C

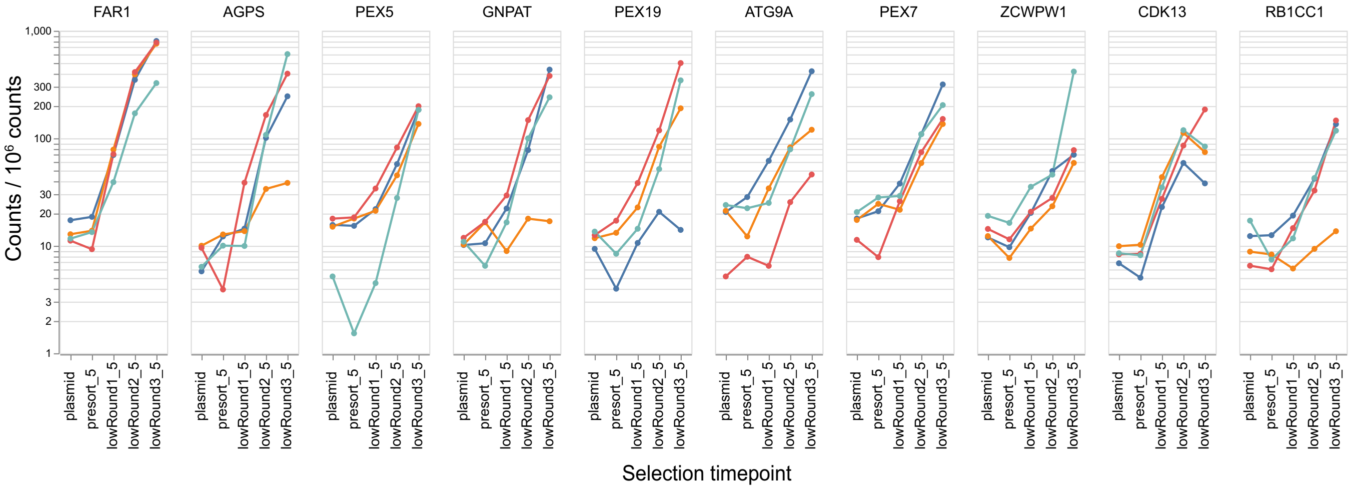

Figure EV2. Further analysis of CRISPR-Cas9 KO screen results. Related to Figure 2.

(**A**) STRING network analysis of enriched genes (log2(fold change) > 1) from the screen. Peroxisome genes are highlighted in red, and autophagy genes in blue. (**B**) GO terms, descriptions, and FDR values of enriched pathways identified by STRING analysis in (**A**). (**C**) Consistent enrichment across individual sgRNAs. For each top-hit gene, per-sgRNA frequencies (counts per million) are plotted across selection time points. "Plasmid" denotes the starting plasmid library, and "presort" denotes the transduced cell population prior to sorting. Each color represents an independent guide targeting a given gene. (**D**) The peroxisomal steps of the plasmalogen synthesis pathway. The final ER step, in which PEDS1 converts plasmanyl to plasmenyl phospholipids, is also highlighted.

© The Author(s)

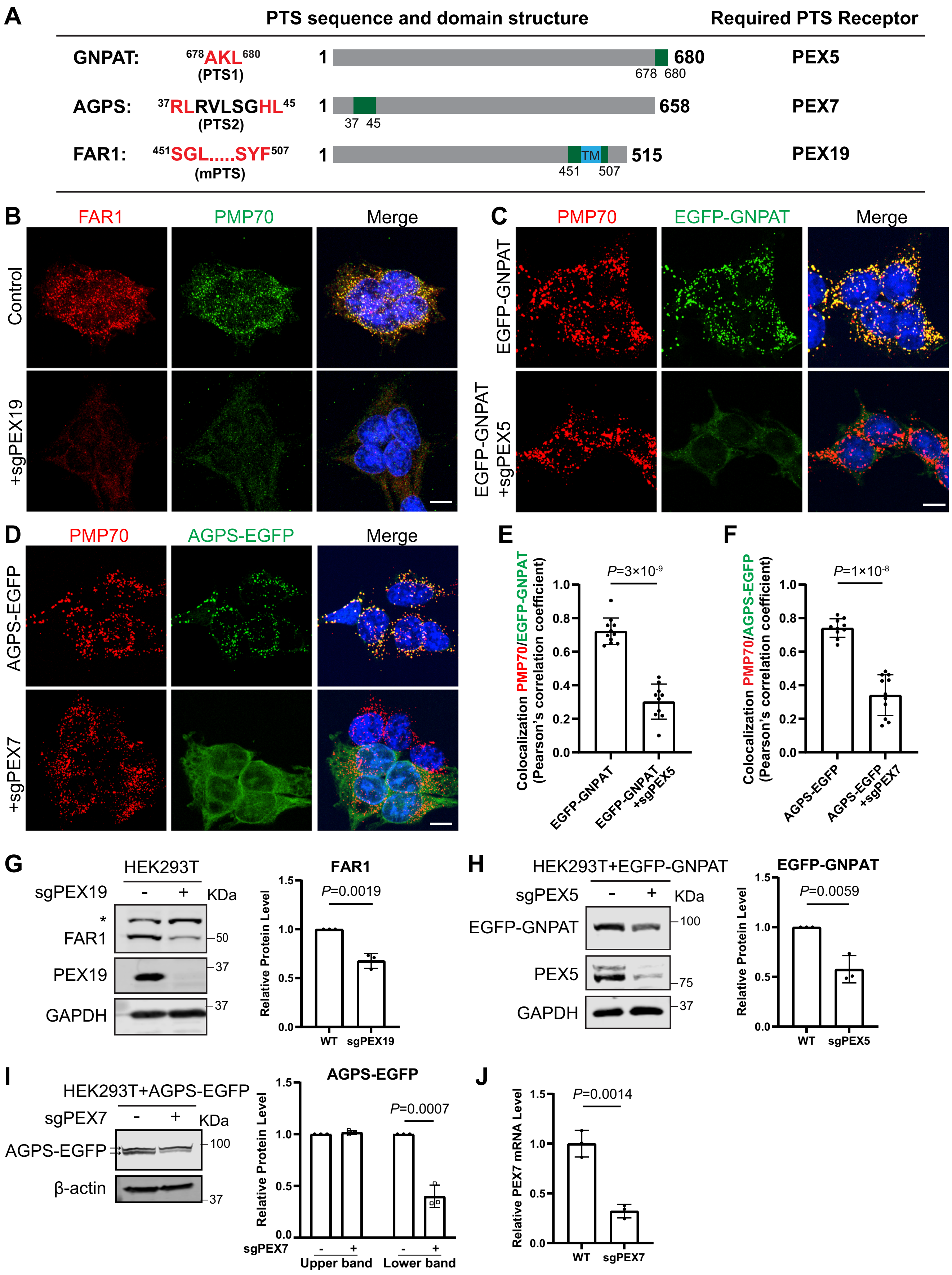
A
PTS sequence and domain structure
Required PTS Receptor
GNPAT: 678AKL680 (PTS1) 1 680 678 680 PEX5
AGPS: 37RLRVLSGHL45 (PTS2) 1 658 37 45 PEX7
FAR1: 451SGL.....SYF507 (mPTS) 1 TM 515 451 507 PEX19
B
FAR1
PMP70
Merge
Control
+sgPEX19
C
PMP70
EGFP-GNPAT
Merge
EGFP-GNPAT
EGFP-GNPAT +sgPEX5
D
PMP70
AGPS-EGFP
Merge
AGPS-EGFP
AGPS-EGFP +sgPEX7
E
Colocalization PMP70/EGFP-GNPAT (Pearson's correlation coefficient)
P=3×10-9
EGFP-GNPAT
EGFP-GNPAT +sgPEX5
F
Colocalization PMP70/AGPS-EGFP (Pearson's correlation coefficient)
P=1×10-8
AGPS-EGFP
AGPS-EGFP +sgPEX7
G
HEK293T
sgPEX19 - + KDa
FAR1
PEX19
GAPDH
50
37
37
FAR1
P=0.0019
Relative Protein Level
WT sgPEX19
H
HEK293T+EGFP-GNPAT
sgPEX5 - + KDa
EGFP-GNPAT
PEX5
GAPDH
100
75
37
EGFP-GNPAT
P=0.0059
Relative Protein Level
WT sgPEX5
I
HEK293T+AGPS-EGFP
sgPEX7 - + KDa
AGPS-EGFP
β-actin
100
37
AGPS-EGFP
P=0.0007
Relative Protein Level
sgPEX7 - + - +
Upper band Lower band
J
P=0.0014
Relative PEX7 mRNA Level
WT sgPEX7

 © The Author(s)

◀ **Figure EV3. Peroxisomal targeting of GNPAT, AGPS, and FAR1 depends on PEX5, PEX7, and PEX19, respectively. Related to Figure 2.**

(**A**) Schematic representations of GNPAT, AGPS, and FAR1 proteins. Peroxisomal targeting sequences and their positions within each protein are highlighted in red and green. The corresponding peroxisomal-targeting receptors are also indicated. TM: transmembrane domain. (**B**) Immunofluorescence images of endogenous FAR1 and PMP70 in WT and *PEX19* KO HEK293T cells. Scale bar: 10 µm. (**C**) Localization of EGFP-GNPAT and endogenous PMP70 in WT and *PEX5* KO HEK293T cells. Scale bar: 10 µm. (**D**) Localization of AGPS-EGFP and endogenous PMP70 in WT and *PEX7* KO HEK293T cells. Scale bar: 10 µm. (**E**, **F**) Pearson's coefficient analysis of co-localization between EGFP-GNPAT and PMP70 in (**C**), and between AGPS-EGFP and PMP70 in (**D**). Data were presented as mean ± s.d.; $n = 10$ ROIs from three biological replicates. *P* values were calculated by a two-tailed unpaired t-test. (**G**) Western blot analysis for endogenous FAR1 in WT and *PEX19* KO HEK293T cells, with quantification. * Indicates a non-specific band. Data were presented as mean ± s.d.; $n = 3$ biological replicates. *P* values were calculated by a two-tailed unpaired t-test. (**H**) Western blot analysis of EGFP-GNPAT in WT and *PEX5* KO HEK293T cells, probed with a GFP antibody. Data were presented as mean ± s.d., $n = 3$ biological replicates. *P* values were calculated using a two-tailed unpaired t-test. (**I**) Western blot analysis of AGPS-EGFP in WT and *PEX7* KO HEK293T cells, probed with a GFP antibody. The upper and lower bands of AGPS-EGFP are indicated by arrows. Data were presented as mean ± s.d., $n = 3$ biological replicates. *P* values were calculated using a two-tailed unpaired t-test. (**J**) RT-qPCR analysis confirms the reduction of *PEX7* mRNA in *PEX7* KO cells. Data were presented as mean ± s.d.; $n = 3$ biological replicates; *P* values were calculated by a two-tailed unpaired t-test. For (**B–D**), these experiments were repeated three times with similar results. Source data are available online for this figure.

© The Author(s)

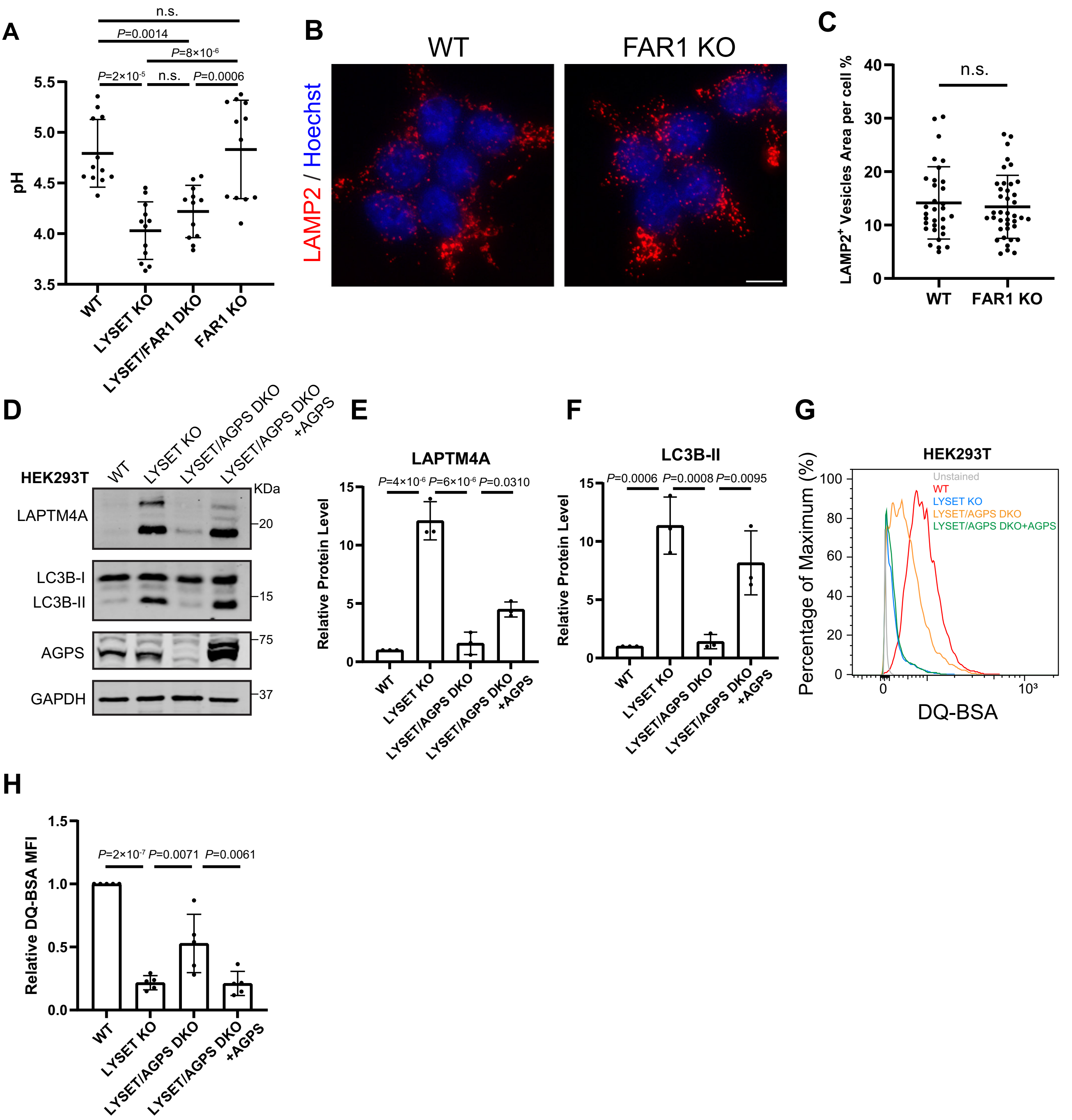

Figure EV4. Effects of ether lipid deficiency on lysosomal pH, number, and protein degradation. Related to Figure 3.

(**A**) Lysosomal pH in WT, *LYSET* KO, *LYSET/FAR1* DKO, and *FAR1* KO HEK293T cells. Cells are stably transfected with the FIRE-pHly (a ratiometric pH biosensor), and the signal intensities of mTFP and mCherry were measured by flow cytometry. Lysosomal pH was calculated from the ratio of mTFP to mCherry signals. Data were presented as mean ± s.d.; $n = 12$ from three biological replicates; *P* values were calculated by one-way ANOVA with multiple comparisons. (**B**, **C**) LAMP2 immunostaining (**B**) and quantification (**C**) in WT and *FAR1* KO HEK293T cells. Data were presented as mean ± s.d.; $n = 32$ cells from three biological replicates; *P* values were calculated by two-tailed unpaired t-test. Scale bar: 10 μm. (**D**) Steady-state levels of LAPTM4A and LC3B-II in WT, *LYSET* KO, *LYSET/AGPS* DKO, and *LYSET/AGPS* DKO cells complemented with AGPS overexpression. (**E**, **F**) Quantification of full-length LAPTM4A and LC3B-II levels in (**D**). Data were presented as mean ± s.d.; $n = 3$ biological replicates; *P* values were calculated by one-way ANOVA with multiple comparisons. (**G**) DQ-BSA assay in WT, *LYSET* KO, *LYSET/AGPS* DKO cells, and *LYSET/AGPS* DKO cells complemented with AGPS overexpression. (**H**) Normalized MFI in (**G**). Data were presented as mean ± s.d.; $n = 5$ biological replicates; *P* values were calculated by one-way ANOVA with multiple comparisons. Source data are available online for this figure.

 © The Author(s)

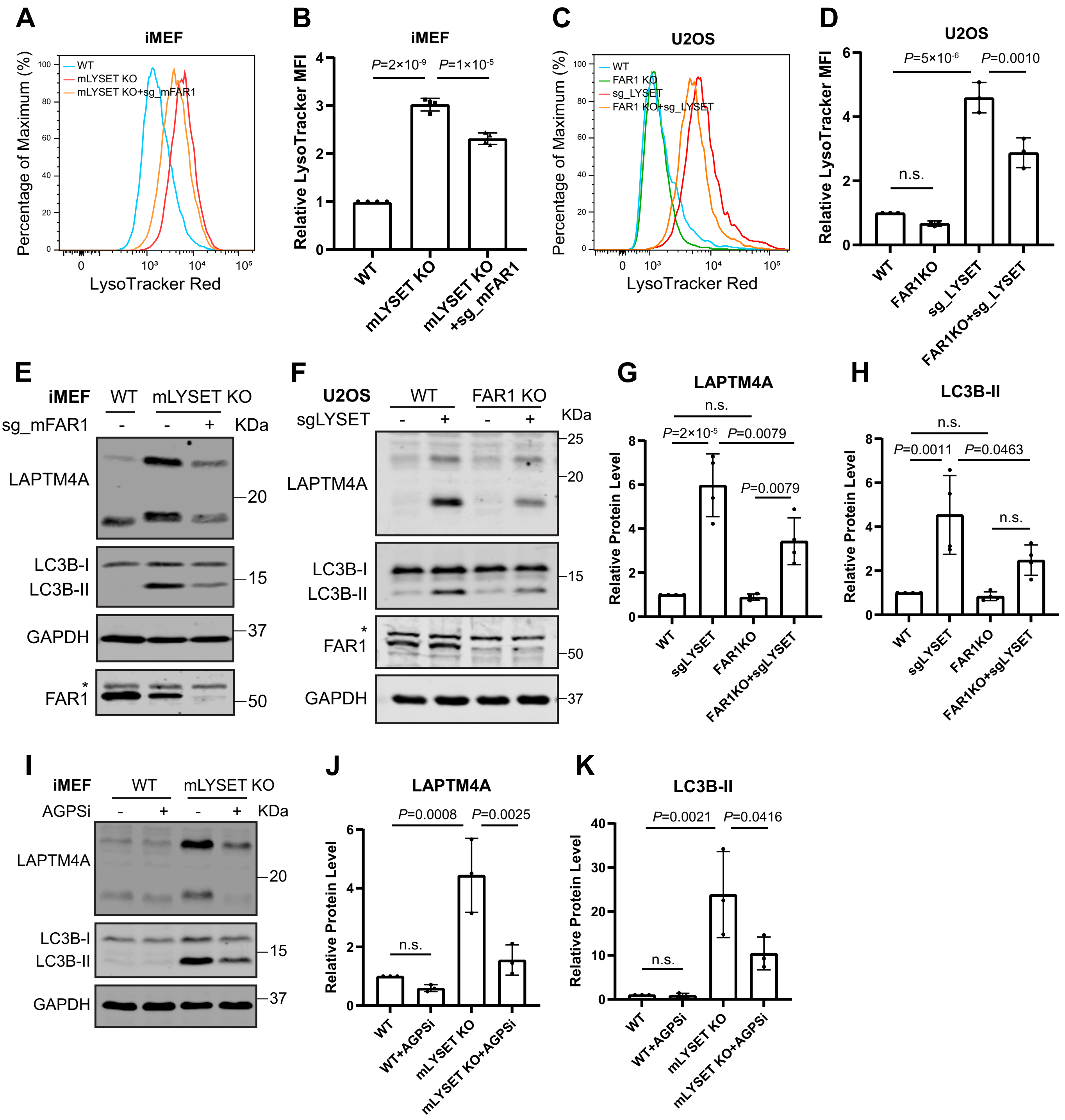

Figure EV5. Conserved effects of the ether lipid synthesis pathway across species and cell lines. Related to Figure 3.

(**A**) LysoTracker Red intensity in WT, *LYSET* KO, and *LYSET/FAR1* DKO iMEF cells. (**B**) Normalized MFI in (**A**). Data were presented as mean ± s.d.; $n = 4$ biological replicates; *P* values were calculated by one-way ANOVA with multiple comparisons. (**C**) LysoTracker Red intensity in WT, *FAR1* KO, *LYSET* KO, and *LYSET/FAR1* DKO U2OS cells. (**D**) Normalized MFI in (**C**). Data were presented as mean ± s.d.; $n = 3$ biological replicates; *P* values were calculated by one-way ANOVA with multiple comparisons. (**E**) Steady-state levels of LAPTM4A and LC3B-II in WT, *LYSET* KO, and *LYSET* KO iMEF cells transfected with FAR1 guide RNA. * Indicates a non-specific band. The experiments were repeated three times with similar results. (**F**) Steady-state levels of LAPTM4A and LC3B-II in WT and *FAR1* KO U2OS cells transfected with or without LYSET guide RNA. * Indicates a non-specific band. (**G, H**) Quantification of total LAPTM4A (**G**) and LC3B-II (**H**) levels in (**F**). Data were presented as mean ± s.d.; $n = 4$ biological replicates; *P* values were calculated by one-way ANOVA with multiple comparisons. (**I**) Steady-state levels of LAPTM4A and LC3B-II in WT, *LYSET* KO iMEF cells treated with or without 250 µM AGPS inhibitor for 48 h. (**J, K**) Quantification of total LAPTM4A (**J**) and LC3B-II (**K**) levels in (**I**), Data were presented as mean ± s.d.; $n = 3$ biological replicates; *P* values were calculated by one-way ANOVA with multiple comparisons. Source data are available online for this figure.

© The Author(s)

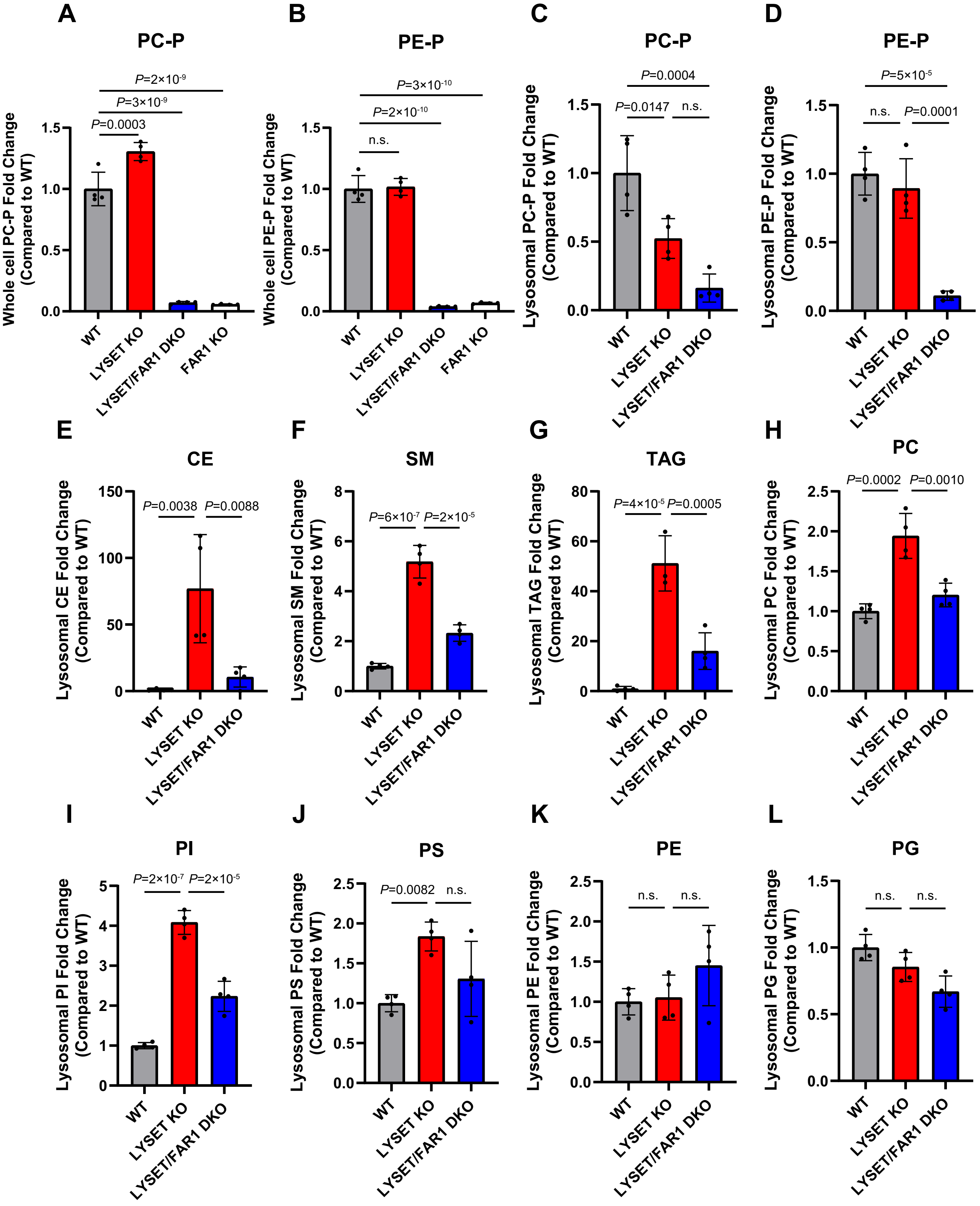
A
PC-P
P=2×10-9
P=3×10-9
P=0.0003
Whole cell PC-P Fold Change (Compared to WT)
WT
LYSET KO
LYSET/FAR1 DKO
FAR1 KO
B
PE-P
P=3×10-10
P=2×10-10
n.s.
Whole cell PE-P Fold Change (Compared to WT)
C
PC-P
P=0.0004
P=0.0147
n.s.
Lysosomal PC-P Fold Change (Compared to WT)
D
PE-P
P=5×10-5
n.s.
P=0.0001
Lysosomal PE-P Fold Change (Compared to WT)
E
CE
P=0.0038
P=0.0088
Lysosomal CE Fold Change (Compared to WT)
F
SM
P=6×10-7
P=2×10-5
Lysosomal SM Fold Change (Compared to WT)
G
TAG
P=4×10-5
P=0.0005
Lysosomal TAG Fold Change (Compared to WT)
H
PC
P=0.0002
P=0.0010
Lysosomal PC Fold Change (Compared to WT)
I
PI
P=2×10-7
P=2×10-5
Lysosomal PI Fold Change (Compared to WT)
J
PS
P=0.0082
n.s.
Lysosomal PS Fold Change (Compared to WT)
K
PE
n.s.
n.s.
Lysosomal PE Fold Change (Compared to WT)
L
PG
n.s.
n.s.
Lysosomal PG Fold Change (Compared to WT)

 © The Author(s)

Figure EV6. Lysosomal lipid accumulation is reduced in *LYSET/FAR1* DKO cells. Related to Figure 5.

(**A**, **B**) Whole-cell plasmenyl-phosphatidylcholine (PC-P) and plasmenyl- phosphatidylethanolamine (PE-P) levels in WT, *LYSET* KO, *LYSET/FAR1* DKO, and *FAR1* KO HEK293T cells. Fold changes were normalized to WT levels. Data were presented as mean ± s.d.; $n = 4$ biological replicates; *P* values were calculated by one-way ANOVA with multiple comparisons. (**C**–**L**) Relative levels of plasmenyl-phosphatidylcholine (PC-P), plasmenyl-phosphatidylethanolamine (PE-P), cholesteryl esters (CE), sphingomyelin (SM), triacylglycerols (TAG), phosphatidylcholine (PC), phosphatidylinositol (PI), phosphatidylserine (PS), phosphatidylethanolamine (PE), and phosphatidylglycerol (PG) in purified lysosomes from WT, *LYSET* KO, and *LYSET/FAR1* DKO HEK293T cells. Fold changes were normalized to WT levels. Data were presented as mean ± s.d.; $n = 4$ biological replicates; *P* values were calculated by one-way ANOVA with multiple comparisons. Source data are available online for this figure.

© The Author(s)

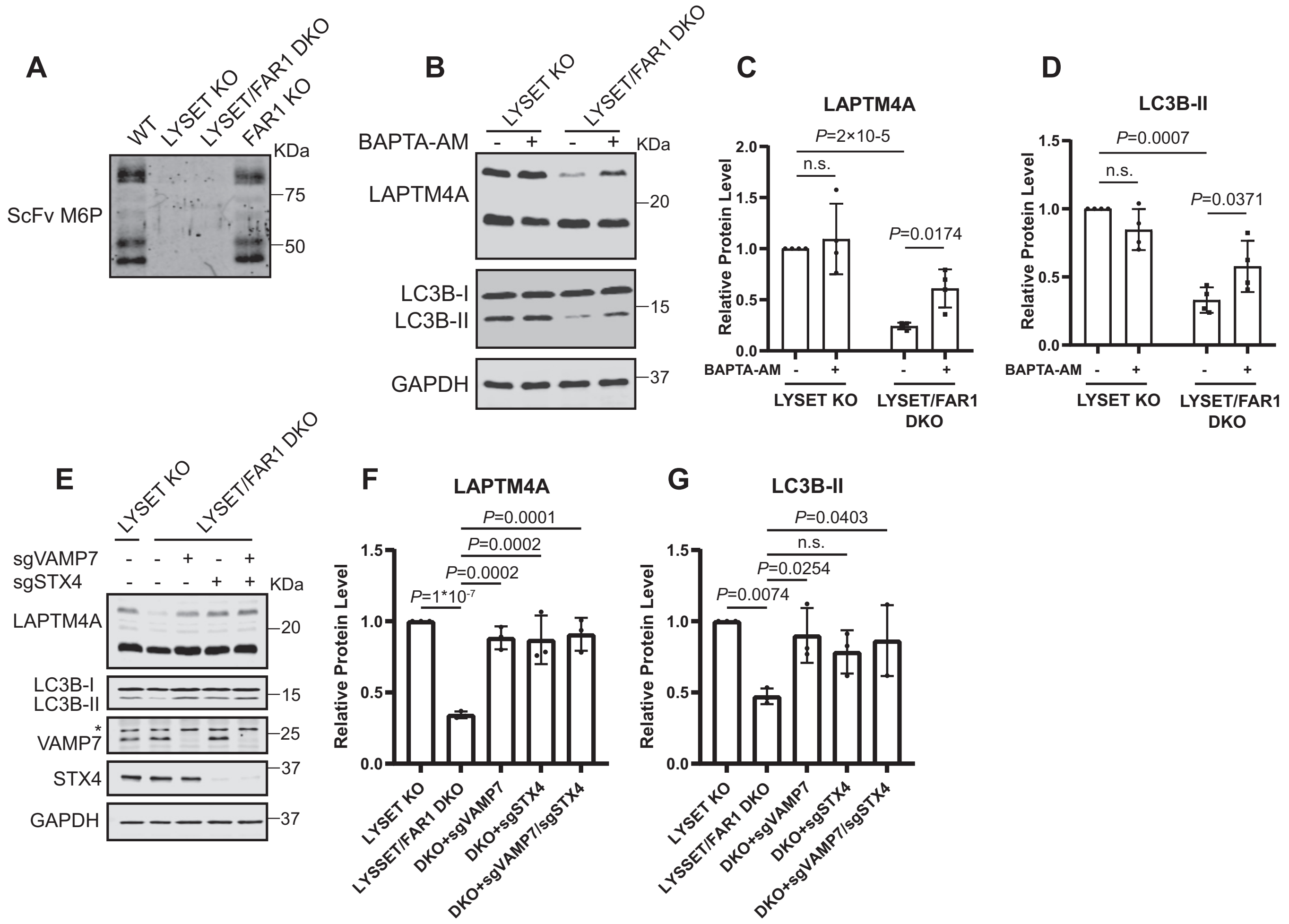

Figure EV7. Inhibition of lysosomal exocytosis restores LAPTM4A and LC3B-II levels in *LYSET/FAR1* DKO cells. Related to Figure 7.

(**A**) Western blot analysis of the M6P modifications in purified lysosomes from the indicated cell lines, detected by a single-chain antibody against M6P (scFv M6P). (**B**) Western blot analysis of LAPTM4A and LC3B-II in *LYSET* KO and *LYSET/FAR1* DKO HEK293T cells treated with or without 10 μM BAPTA-AM for 48 h. (**C**, **D**) Normalized protein levels of full-length LAPTM4A and LC3B-II in (**B**). Data were presented as mean ± s.d.; $n = 4$ biological replicates; *P* values were calculated by two-tailed paired t-test. (**E**) Western blot analysis of LAPTM4A and LC3B-II in *LYSET* KO, *LYSET/FAR1* DKO, *LYSET/FAR1/VAMP7* TKO, *LYSET/FAR1/STX4* TKO, and *LYSET/FAR1/VAMP7/STX4* QKO HEK293T cells. * Indicates a non-specific band. (**F**, **G**) Normalized protein levels of full-length LAPTM4A and LC3B-II in (**E**), Data were presented as mean ± s.d.; $n = 3$ biological replicates; *P* values were calculated by one-way ANOVA with multiple comparisons. Source data are available online for this figure.

 © The Author(s)
